# Comprehensive genomic profiling of urothelial carcinoma cell lines reveals hidden research bias and caveats

**DOI:** 10.1002/ctm2.36

**Published:** 2020-04-30

**Authors:** Yongwen Luo, Lingao Ju, Gang Wang, Chen Chen, Yejinpeng Wang, Liang Chen, Yi Zhang, Yu Xiao, Xinghuan Wang

**Affiliations:** ^1^ Department of Urology Zhongnan Hospital of Wuhan University Wuhan China; ^2^ Human Genetic Resources Preservation Center of Hubei Province Wuhan China; ^3^ Cancer Precision Diagnosis and Treatment and Translational Medicine Hubei Enginnering Research Center Wuhan China; ^4^ Center of Life Sciences Peking University Beijing China

Dear Editor,

Preexisting genetic mutations in cells may influence phenotypes together with other molecular biology manipulations. For instance, the bladder cancer cell RT4 harbors a *TACC3‐FGFR3* fusion rather than *TP53* mutation (Figure 1F), whereas the other bladder cancer cell T24 have *TP53* mutation but not *FGFR3* mutation.^1^ We questioned whether those preexisting genotypes could influence research conclusions and systematically overviewed urothelial carcinoma‐related 1589 articles either used or not used RT4 (Table S1).

**FIGURE 1:**
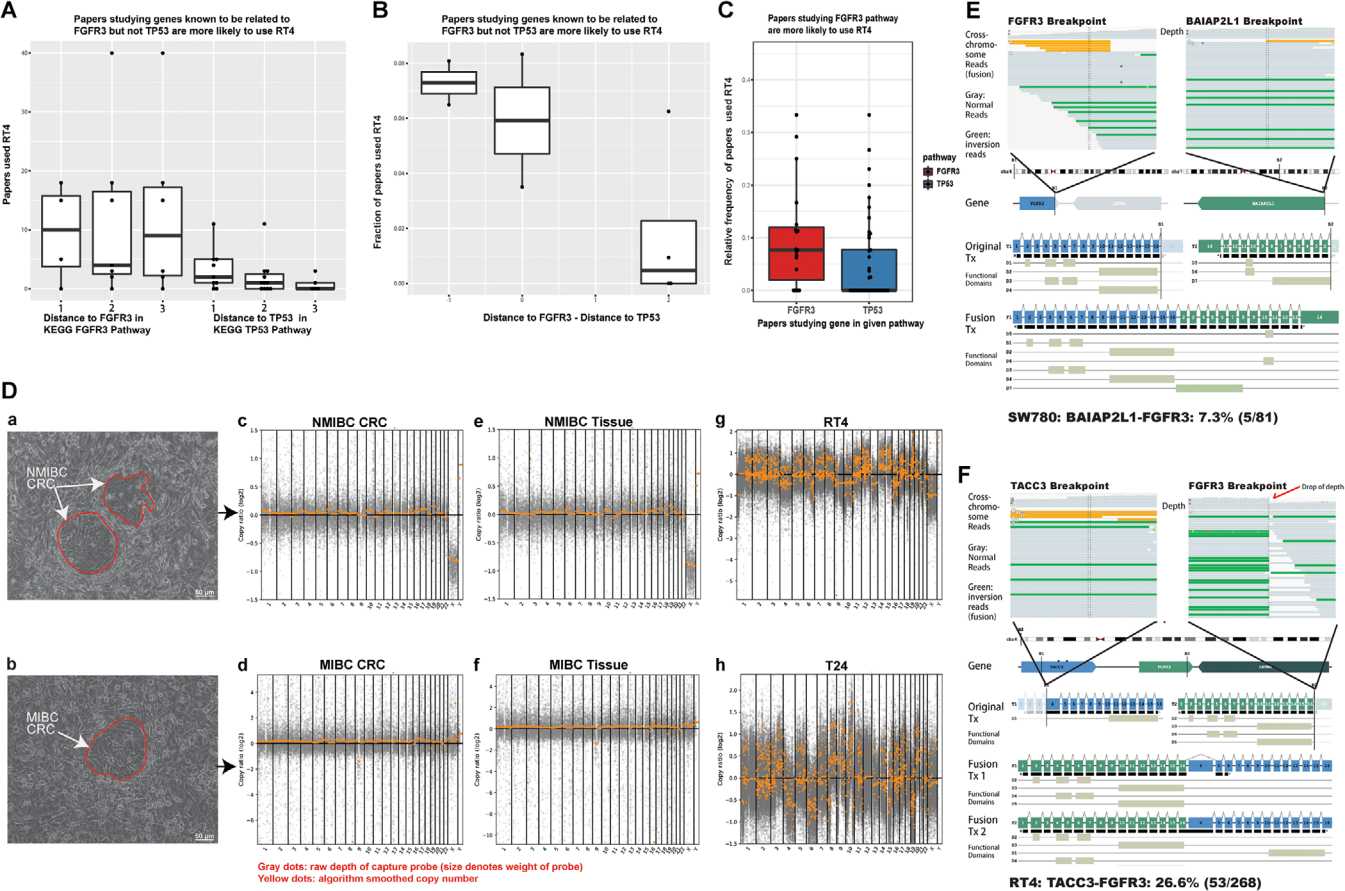
Comprehensive genomic profiling of urothelial carcinoma cell lines reveals hidden research bias and caveats. A, Distance from given target gene to TP53 on KEGG pathway is negatively correlated with RT4 usage. *X*‐axis: distance to TP53 and FGFR3 (1, 2, and 3 denote direct interacting with TP53, one‐step away from TP53, and two‐step away from TP53, etc.). *Y*‐axis: PubMed search result number of target gene name with RT4. B, The relative distance from given target gene to TP53 and FGFR3 based on protein‐protein interaction network is correlated with RT4 usage. *X*‐axis: The difference between target‐gene‐to‐TP53 distance and target‐gene‐to‐FGFR3 distance. A minus number denotes the gene is closer to FGFR3 compared to TP53, and a positive number denotes the gene is closer to TP53 compared to FGFR3. *Y*‐axis: Frequency of RT4‐containing search result in all PubMed search results with target gene and “bladder cancer cell line.” C, Papers studying FGFR3 pathway are more likely to use RT4 density distribution of the RT4‐containing search result frequency on PubMed for target genes in the KEGG FGFR3‐related signaling pathway or TP53‐related signaling pathway. D, Morphology of 2 BCa patient‐derived CRCs (a‐b). Scale bar is 50 μm. Visually pan‐genome copy number profile (yellow) with sequencing depth of probe region (gray) generated by CNVkit from 2 CRCs (c‐d) and 2 BCa tissue (e‐f) together with BCa cell lines T24 and RT4 (g‐h) showing the significant deviation from genuine BCa cell lines. E, Sequencing evidence for heterogeneity of driver oncogene fusion in SW780 cell line. Top panel showing the raw sequencing reads (yellow, cross‐chromosomal DNA fragments; gray, normal DNA fragments; green, fragments in inverted direction) of FGFR3 (top‐left) and BAIAP2L1 (top‐right) loci. Middle panel showing the chromosomal bands and gene structure, denoting the breakpoint locus. Bottom panel showing the original transcript (Tx) and respective functional domains of each fusion partner gene, and the predicted fusion product transcript with a functional FGFR3 kinase domain and longer C‐termini from BAIAP2L1. DNA fragments in support of fusion consist 7.3% (five in 81) of all sequenced fragments, suggesting that the fusion driver oncogene is lost in a part of cells. F, Sequencing evidence for TACC3‐FGFR3 driver oncogene fusion in RT4 cell line. Panel layout is similar to (E). DNA fragments in support of fusion consist 26.6% (53 in 268) of all sequenced fragments, suggesting that the fusion driver oncogene likely to exist in homogeneous one‐in‐a‐tetraploid cell state or in a heterogeneous manner. Copy number profile (a in panel D), however, did suggest that significant level of mosaicism exists in the RT4 population.

We found a significant association between target gene corresponding pathways and RT4 usage frequency. For example, the research was less likely to include RT4 (Figures 1A and 1B and Table S2) if the target gene was associate with TP53 pathways. We calculated the frequency of using RT4 and/or T24 for research on particular target gene (Table S3). Using PPI networks,^2^ we calculated the “interaction distance” between these genes to TP53 and FGFR3. RT4 usage frequency was increased in research of genes with closer relationship to FGFR3 compared to TP53 (Figure 1C). Those results suggested the existence of latent bias in research, that is, researcher may implicitly select consistent results by selecting carcinoma cell lines. The preexisting carcinoma cell line mutation landscape could determine its response to particular molecular biology manipulation, which can be inconsistent, even though only consistent results would be reported.

Moreover, to define the genotype of carcinoma, cell line is critical to the research result. State‐of‐the‐art practices require genotyping cell lines using short tandem repeat (STR).^3^ However, STR only identifies whether cell lines are cross‐contaminated, but does not fully report the genomic changes of cell lines. We collected eight most widely used bladder cancer cell lines and confirmed the identity by STR. Whole‐exome sequencing (WES) of the eight cell lines together with two conditionally reprogrammed cells (CRCs)^4^ and two bladder cancer tissues (Table S4) showed the CRCs have good consistency with tumor tissues; however, the cell lines showed significant deviation from genuine bladder cancer by wide‐spread shattering copy number variation (Figures 1D and S1). Furthermore, comparing with the previous reports,^5^ we detected only a tiny fraction of the known driver FGFR3 fusion in SW780 (Figure 1E). Those results indicated that the carcinoma cell lines may undergone pervasive genetic drifts characterized by copy number variation and random loss of driver mutation.

In conclusion, carcinoma cell line genotypes could influence research results by modifying molecular manipulation outcomes. Such influences could be implicit or explicit, and represent researcher's selection bias in research. Furthermore, the genotypes undergone neutral genetic drift, which might lead to loss of important driver mutation and gain of novel genetic identity. Thus, it is important that WES, instead of STR, should be widely used for determining carcinoma cell line genotypes. The CRCs closely resemble original tumor tissue and represent a better alternative for in vitro research to benefit the development of future precise medicine.

## CONFLICT OF INTEREST

The authors declare no conflict of interest.

## Supporting information

Supplement Information
