# Supplementary material for: Comprehensive genomic profiling of urothelial carcinoma cell lines reveals hidden research bias and caveats
Source: Clin Transl Med. 2020 Apr 30;10(1):294–6. doi: 10.1002/ctm2.36 (PMC7240854; doi:10.1002/ctm2.36)
Supplement: Supplementary file 1 — Supplement Information [file CTM2-10-294-s001.pdf]

Supplementary information

Table of Contents

Supplementary material and methods…………………………………………..2

Ethics approval and consent to participate………………………………………...2

Literature search strategy and selection of studies…………………………………...2

Patient selection and pathological classification……………………………………....3

Conditional reprogrammed cells (CRC) technology…………………………………….3

Cell culture…………………………………………………………………………………4

DNA extraction and NGS library preparation……………………………………………..4

Bioinformatic pre-processing and determination of somatic mutation events……...5

Quality control of sequenced samples……………………………………………………6

Determine driver variants………………………………………………………………...6

Reference………………………………………………………………………………...8

Supplementary Figures…………………………………………………………9

Figure S1…………………………………………………………………………………...9

Supplementary Tables…………………………………………………………10

Table S1…………………………………………………………………………………...10

Table S2…………………………………………………………………………………106

Table S3…………………………………………………………………………………108

Table S4…………………………………………………………………………………110

Supplementary material and methods

Ethics approval and consent to participate

The study using clinical information and human samples (including surgical tissue specimens and primary cancer cells) was approved by the Ethics Review Committee at Zhongnan Hospital of Wuhan University (approval number: 2015029). Human sample preservation by the Zhongnan Hospital Biobank, the official member of the International Society for Biological and Environmental Repositories (https://irlocator.isber.org/details/60), was approved by the Ethics Review Committee at Zhongnan Hospital of Wuhan University (approval number: 2017038) and China Human Genetic Resources Management Office, Ministry of Science and Technology of China (approval number: 20171793).

Literature search strategy and selection of studies

We performed an electronic search of PubMed (1966 to January 2019, *https://www.ncbi.nlm.nih.gov/pubmed*), for evaluating the relationship of preexisting genotype of BCa cell and research results on other genes. The search keywords were used with different combinations with both medical subject headings terms and text words: "(RT4[TW] OR RT-4[TW] OR T24[TW]) AND ("urinary bladder neoplasms" [MeSH Terms] OR "bladder neoplasms" [TIAB] OR "bladder cancer" [TIAB] OR "bladder tumour" [TIAB] OR "bladder tumor" [TIAB])". Publication date was not restricted in our search. Reference lists of the included studies and supplemental materials were checked manually to further identify related studies. Three reviewers independently screened the title, abstract and keywords of each article retrieved. Full-text papers were screened for further assessment if the information given suggested that the study fulfilled the inclusion criteria and did not meet the exclusion criteria. Discrepancies were settled by discussion and consensus with all the authors.

Patient selection and pathological classification

The clinical, pathological, follow-up data records of two bladder cancer (BCa) patients were collected (Table S4), and two pathologists were invited to confirm the histology diagnosis independently. The study using clinical information and human samples (including surgical tissue specimens and primary cancer cells) was approved by the Ethics Committee at Zhongnan Hospital of Wuhan University (approval number: 2015029). The human sample preservation by Department of Biological Repositories at Zhongnan Hospital of Wuhan University, the official member of the International Society for Biological and Environmental Repositories-International Repository Locator (ISBER-IRL) [1], was approved by the Ethics Committee (approval number: 2017038) and China Human Genetic Resources Management Office, Ministry of Science and Technology of The People's Republic of China (approval number: 20171793). All BCa patients provided the written informed consent. The procedures in this study were done in accordance with the ethical standards of the institutional ethics review committee.

Conditional reprogrammed cells (CRC) technology

The method used to establish patient-derived conditional reprogramming (CR) bladder cancer cell model from fresh BCa tissues was performed according to Liu *et al.* with minor modifications [2]. Briefly, two fresh BCa tissues were rinsed with 95% ethanol (up to 3 seconds) and cold PBS. Chop the samples into small (< 10 mm) pieces with scissors. Then transfer the tissue fragments to 4 ml F medium, containing collagenase, hyaluronidase and dispase, and incubate for 2 hours at 37 °C on a rocking platform. After dissociation, the cell pellet was collected by centrifuging for 5 minutes at 500 g and resuspend in 10 ml complete DMEM. The cell suspension was filtered by 100 mm cell strainer, followed by centrifugation for 5 minutes at 300 g. Then plate the processed cell pellet in a T25 flask together with Swiss-3T3-J2 mouse

fibroblasts feeder cells in complete F medium. Replace feeder cells every three days and observe colonies of CR prostate cancer cells by phase contrast microscopy (Leica Ltd., Germany).

Cell culture

All urothelial cell lines were purchased from Cell Bank of the Chinese Academy of Sciences. The cell lines are authenticated via short tandem repeat (STR) analysis. The medium and culture conditions are as follows:

Cell line	Medium supplemented with 10% FBS	Culture conditions
RT4	McCoy's 5A	37°C, 5% CO2
T24	RPMI-1640	37°C, 5% CO2
UM-UC-3	MEM	37°C, 5% CO2
J82	MEM	37°C, 5% CO2
SCaBER	RPMI-1640	37°C, 5% CO2
5637	RPMI-1640	37°C, 5% CO2
SW780	L-15	37°C, free gas exchange with atmospheric air
SV-HUC-1	F-12K	37°C, 5% CO2

DNA extraction and NGS library preparation

Genomic DNA is extracted from cultured cells using Qiagen tissue DNA column. We processed the DNA according to a modified single-stranded DNA sequencing library prep protocol. Briefly, genomic DNA were sonicated, 3'-poly-A tailed with terminal transferase, ligated with a poly-dT-tailed P5 sequencing adaptor, and undergone several cycles of linear amplification using P5 sequencing primer. A P7 adaptor with random nucleotide 3' overhang was then ligated to the 3' end of linear amplification product. From there, sequencing library were amplified using P7+P5 primers to sufficient amount. Hybrid capture were performed

using standard DNA probe capture practice with IDT XGEN whole exome panel. The post-capture libraries were sequenced on Illumina Novaseq.

Bioinformatic pre-processing and determination of somatic mutation events

For SNV/indel and CNV calls, reads were trimmed using cutadapt to remove poly-T-tails, adaptors and the first 10bp from each end before being mapped to GRCh37+decoy reference genome using bwa-mem. For structural variation calls, reads were only trimmed to remove adaptors before mapping. Before calling any variants, the bam files were duplication-marked, realigned, and passed through a BQSR pipeline. SNV (including single nucleotide variation and small indel, hereinafter designated as SNV) calls are made with Sentieon TNscope with sequenced NA12878 cell line as control, or with Pisces with tumor sample only. The tumor cell and control samples additionally run through a Sentieon Haplotyper pipeline individually to call AF > 0.2 variants. The TNscope callset were filtered with AF(tumor) > 0.2, ALT_F1R2 > 5, ALT_F2R1 > 5, ALT >= 30, AF(tumor)/AF(control) > 5, AF(normal) < 0.01. The Pisces callset were used to calibrate oxo-G levels using VQR. After calibration, Pisces callset were intersected with TNscope callset to generate a "somatic callset". Finally, germline mutations from the normal sample Haplotyper callset were removed from the somatic callset. Annotations were done with Annovar and VEP. We further annotated the mutations with gnomAD (*http://gnomad-old.broadinstitute.org/*), MCAP, Spidex, SCADA, Clinvar (*https://www.ncbi.nlm.nih.gov/clinvar/*) and public HGMD databases (*http://www.hgmd.cf.ac.uk/ac/index.php*). Sequenza was used to process tumor sample to estimate cellularity (tumor fraction). To call somatic CNV, copy-number-homogeneous segments were generated using CNVkit run with tumor and paired normal samples, and filtered with cellularity. We passed BAF from the somatic callset as well as germline Haplotypecaller callset into sequenza and CNVkit, to estimate correct copy number information for each

segment. Structural variation was called and annotated using the iCallSV pipeline. Mavis (using Lumpy) was also used to search for possible low-frequency SV. MSI score is calculated with MSIsensor. To calculate mutation signature, the filtered, passed, main- and subclonal mutations were passed to deconstructSig.

Quality control of sequenced samples

Quality control of the files was done with in-house script. We checked the mapping quality, mapping rate, duplication rate and uniformity to control for molecular assay and sequencing. Tumor samples in this study were sequenced to a median of > 200x. Failed regions (< 5x) were < 0.2% in any sequenced samples. For each tumor sample, > 98% of captured regions achieved a minimum of 50x.

Determine driver variants

Mutations were classified using gnomAD prevalence, pathogenicity prediction, and known evidence from Clinvar and HGMD open. For LP and PAT mutations in HGMD, we used a population frequency cutoff of 0.002. For gnomAD alleles, we used a population frequency cutoff of 0.001, East Asian allele count (AC_EAS) < 10, and homozygous East Asian donor count (Hom_EAS) < 2. Alleles without gnomAD frequency were annotated against an in-house population frequency library consisting 5000 Chinese WES/WGS data. We filtered severe mutations (frameshift, inframe-del, inframe-ins, stop-gain, splicing defect with SCADA > 0.8 or abs(Spidex) > 3). For missense or indel mutations, we require the locus MCAP > 0.02. For common known oncogene and tumor suppressors, we further validated mutations using the list provided by Bailey et al 2018 and Clinvar [3, 4]. Identification of missense driver mutations is done by filtering against COSMIC database (*https://cancer.sanger.ac.uk/cosmic/signatures/ID*).

To calculate the cancer specificity of any missense driver event, we computed the relative prevalence of the mutation in gnomAD plus the in-house Chinese germline database and compared it to the prevalence of this mutation in TCGA (*https://portal.gdc.cancer.gov/*) and COSMIC database using Fisher's exact test. We found that a prevalence of 5 in COSMIC v.88 database, or 3 in TCGA MC3 MAF file, with a gnomAD global population frequency lower than 0.1%, is adequate to determine that the mutation is recurrent and somatic in nature for tumor tissues. For LOF-driver genes, we also included likely pathogenic splicing/start-lost/stop-gain/frameshift mutations, as well as CNV-loss. For GOF-driver genes, we included high level CNV-gain (normalized to >= 5x). Structural variation results were filtered to include only the kinase-domain-included, activating translocations, and a frequency of > 25%.

Reference

[1] ISBER. Zhongnan Hospital Biobank, China. https://irlocator.isber.org/details/60.

[2] Liu X, Krawczyk E, Suprynowicz FA, et al. Conditional reprogramming and long-term expansion of normal and tumor cells from human biospecimens. Nat Protoc. 2017;12:439-51.

[3] Bailey MH, Tokheim C, Porta-Pardo E, et al. Comprehensive Characterization of Cancer Driver Genes and Mutations. Cell. 2018;173:371-85.e18.

[4] Landrum MJ, Lee JM, Benson M, et al. ClinVar: public archive of interpretations of clinically relevant variants. Nucleic Acids Res. 2016;44:D862-8.

Supplementary Figures

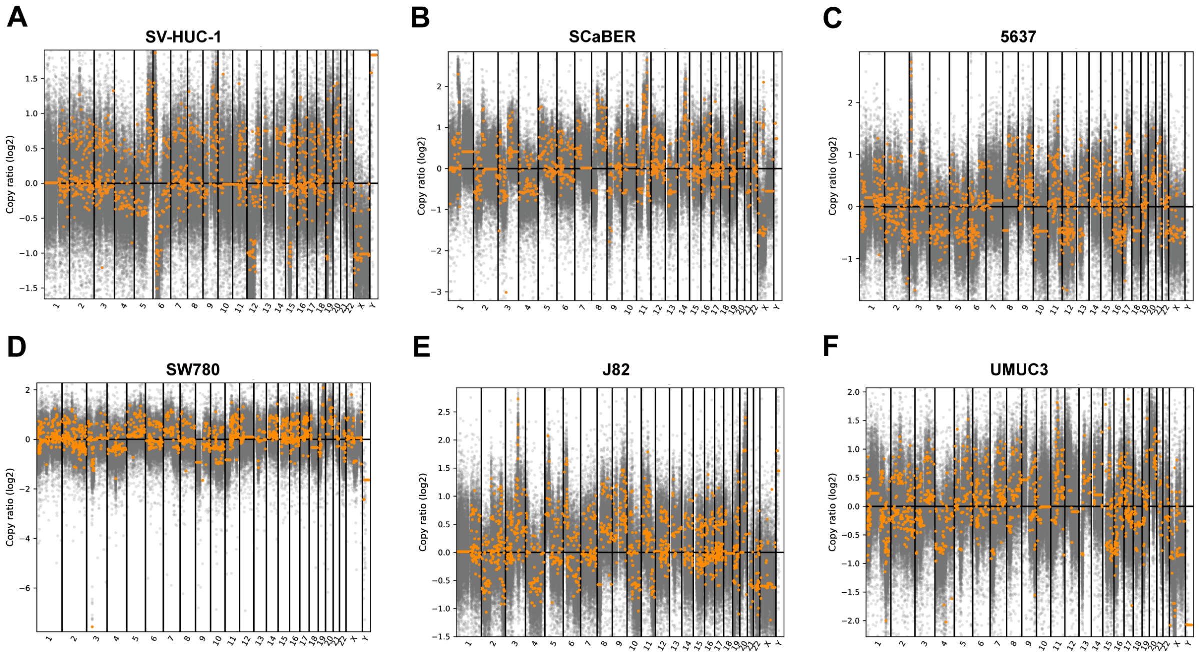

Figure S1. Pan-genome copy number variations plots of urothelial cells. Visually pan-genome copy number profile (yellow) with sequencing depth of probe region (gray) generated by CNVkit from SV-HUC-1, SCaBER, 5637, SW780, J82 and UMUC3.

Supplementary Tables

Table S1. The list of bladder cancer related articles either used or not used RT4.

Author	Year	PMID	using RT4 or not(0 represents No;1 represents Yes)	Title	Gene	Pathway
Chen Y et al.	2019	30527358	0	Knockdown of lncRNA SNHG7 inhibited cell proliferation and migration in bladder cancer through activating Wnt/β-catenin pathway.	SNHG7、β-catenin、Cyclin D1、E-cadherin、MYC	SNHG7-Wnt-β-catenin
Cao R et al.	2018	29175458	1	TM4SF1 regulates apoptosis, cell cycle and ROS metabolism via the PPARγ-SIRT1 feedback loop in human bladder cancer cells.	TM4SF1、Bax、Bcl-xl、Caspase3、Caspase6、Caspase7、Caspase9、SIRT1、PPARγ、FOXO3a、Catales、SOD2、CCNA1/2、CCND1、CCDK2、CDK4	PPARγ-SIRT1
Zhu, Y. D et al.	2018	30066919	0	Increased expression of TNFRSF14 indicates good prognosis and inhibits bladder cancer proliferation by promoting apoptosis	TNFRSF14	
Zhu, Y et al.	2018	29872024	0	Inhibition of miR-1247 on cell proliferation and invasion in bladder cancer through its downstream target of RAB36	miR-1247	RAB36
Zhu, J et al.	2018	29467870	0	HIF-1alpha promotes ZEB1 expression and EMT in a human bladder cancer lung metastasis animal model	HIF-1α、ZEB1	
Zhou, Q et al.	2018	30066004	0	HER2 and Src co-regulate proliferation, migration and transformation by downstream signaling pathways in arsenite-treated human uroepithelial cells	cyclin D1, COX2, PCNA, VEGF, and HIF-1α	Ras/Raf/MAPK, PI3K/AKT, and JAK2/STAT3
Zhen, S et al.	2018	30457360	0	Synergistic Antitumor Effect on Bladder Cancer by Rational Combination of Programmed Cell Death 1 Blockade and CRISPR-Cas9-Mediated Long Non-Coding RNA Urothelial Carcinoma Associated 1 Knockout	CRISPR-Cas9,UCA1 and PD-1	PD-1/PD-L1
Zhao, L et al.	2018	30197677	0	MAPK/AP-1 pathway regulates benzidine-induced cell proliferation through the control of cell cycle in human normal bladder epithelial cells	cyclinD1, PCNA,CDKs,p21,ERK1/2,p38 ,JNK	MAPK/AP-1,ERK1/2,JNK/MAPK
Zhao, J et al.	2018	29602637	1	Importin-11 overexpression promotes the migration, invasion, and progression of bladder cancer associated with the deregulation of CDKN1A and THBS1	Importin-11, CDKN1A, HBEGF, PTK2, THBS1, CCNG2, and EGR1	p53, ErbB signaling pathway,BCa pathway, PGs in cancer pathway
Zhang, T et al.	2018	30015971	0	Suppression of the SDF1/CXCR4/betacatenin axis contributes to bladder cancer cell growth inhibition in vitro and in vivo	SDF-1/CXCR4/β-catenin	
Zhang, T et al.	2018	30184315	0	Brazilin induces T24 cell death through c-Fos and GADD45beta independently regulated genes and pathways	c‑Fos, GADD45β	GADD45α‑p38‑NF‑κB,MKK4‑JNK, AP-1,
Zhang, S et al.	2018	30304549	0	Comprehensive analysis of microRNA-messenger RNA regulatory network in gemcitabine-resistant bladder cancer cells	miR‑182‑5p, miR‑590‑3p, miR‑320a ,SGK1,IL6, TNF, KIF11, BUB1B, and POLE2	ATM/NF‑kappa B
Zhang, M et al.	2018	29981725	0	Thymoquinone induces apoptosis in bladder cancer cell via endoplasmic reticulum stress-dependent mitochondrial pathway	Bcl-2, Bax, cytochrome c,GRP78, CHOP,caspase-12 ,caspase-3, caspase-8, caspase-7, PARP, ATF6, PERK, and IRE1	ER stress pathway,mitochondrion-mediated apoptotic pathway
Zhang, J et al.	2018	30458442	0	miR-1-3p Contributes to Cell Proliferation and Invasion by Targeting Glutaminase in Bladder Cancer Cells	miR-1-3p	
Zhang, J et al.	2018	30176759	1	MicroRNA-325 Is a Potential Biomarker and Tumor Regulator in Human Bladder Cancer	MicroRNA-325	

Table S1. The list of bladder cancer related articles either used or not used RT4.

Author	Year	PMID	using RT4 or not(0 represents No;1 represents Yes)	Title	Gene	Pathway
Zhai, X et al.	2018	29321082	0	Long Noncoding RNA ATB Promotes Proliferation, Migration, and Invasion in Bladder Cancer by Suppressing MicroRNA-126	lncRNA-ATB, miR-126, and KRAS	PI3K/AKT and mTOR signal pathways
Zeng, X et al.	2018	29154981	0	Epidermal Growth Factor Receptor Family Inhibition Identifies P38 Mitogen-activated Protein Kinase as a Potential Therapeutic Target in Bladder Cancer	EGFR，HER2	p38 MAPK，
Yun, B. H et al.	2018	30001485	1	Method for Biomonitoring DNA Adducts in Exfoliated Urinary Cells by Mass Spectrometry	4-ABP，AA-I	
Yang, Y et al.	2018	30119201	0	Synergistic antitumour effects of triptolide plus gemcitabine in bladder cancer	CDK4、CDK6和cyclins A1、A2,caspase 8和Bcl-xL	AKT / GSK3β
Yang, Y et al.	2018	30505198	0	Bladder cancer cell viability inhibition and apoptosis induction by baicalein through targeting the expression of anti-apoptotic genes	BCL2、Bcl-xL、XIAP	
Yang, X et al.	2018	29117631	0	Astrocyte elevated gene-1 promotes invasion and epithelial-mesenchymal transition in bladder cancer cells through activation of signal transducer and activator of transcription 3	AEG‑1	STAT3
Yang, J et al.	2018	29702481	1	Paeoniflorin inhibits the growth of bladder carcinoma via deactivation of STAT3.	Paeoniflorin (Pae)， caspase3, caspase8 and caspase9	STAT3
Yang, H. J et al.	2018	29349903	1	GP73 promotes invasion and metastasis of bladder cancer by regulating the epithelial-mesenchymal transition through the TGF-beta1/Smad2 signalling pathway	GP73	TGF-beta1/Smad2 signalling pathway
Yan, L et al.	2018	28799673	1	TPX2-p53-GLIPR1 regulatory circuitry in cell proliferation, invasion, and tumor growth of bladder cancer	TPX2, p53, GLIPR1, SP1, and c‑Myb	TPX2-p53-GLIPR
Yadav, D et al.	2018	30261824	0	3D-QSAR and Docking studies on Ursolic acid derivatives for Anticancer activity based on Bladder cell line T24 targeting NF-kB pathway inhibition	3D-QSAR	NF-kB pathway
Xue, Y et al.	2018	30104883	0	Combination chemotherapy with Zyflamend reduced the acquired resistance of bladder cancer cells to cisplatin through inhibiting NFkappaB signaling pathway	RelA，MMP9	NFkappaB signaling pathway
Xu, Z. L et al.	2018	30132557	0	B7H3 promotes malignant progression of muscleinvasive bladder cancer	B7H3	
Xu, T et al.	2018	29406097	0	Elevated 8-oxo-7,8-dihydro-2'-deoxyguanosine in genome of T24 bladder cancer cells induced by halobenzoquinones	TCBQ, TBBQ, 2,5-DCBQ and 2,6-DCBQ	
Xu, T et al.	2018	29467898	0	Anticancer effect of miR-96 inhibitor in bladder cancer cell lines	miR-96，HERG1	HERG1
Xie, H et al.	2018	29501702	0	Synthetic artificial "long non-coding RNAs" targeting oncogenic microRNAs and transcriptional factors inhibit malignant phenotypes of bladder cancer cells	miR-183, miR-182, miR96， miR-17-5p，Bcl-XL, TRAF1，c-myc, cyclin D1	NF-kB pathway
Xiao, J et al.	2018	29620229	0	miR223p enhances multichemoresistance by targeting NET1 in bladder cancer cells	miR223p， NET1	
Xiang, X et al.	2018	29435036	0	Long non-coding RNA cartilage injury-related promotes malignancy in bladder cancer	lncRNA-CIR	

Table S1. The list of bladder cancer related articles either used or not used RT4.

Author	Year	PMID	using RT4 or not(0 represents No;1 represents Yes)	Title	Gene	Pathway
Xia, Y et al.	2018	29945448	0	Apigenin Suppresses the IL-1beta-Induced Expression of the Urokinase-Type Plasminogen Activator Receptor by Inhibiting MAPK-Mediated AP-1 and NF-kappaB Signaling in Human Bladder Cancer T24 Cells	IL-1β，uPAR	AP-1 and NF-kappaB Signaling，IL-1β-Activated MAPK (ERK1/2 and JNK) Pathways
Wu, K et al.	2018	30272373	0	miRNA26a5p and miR26b5p inhibit the proliferation of bladder cancer cells by regulating PDCD10	miR-26a-5p/miR-26b-5pPDCD10	
Wu, H et al.	2018	29391086	0	B7-Homolog 4 Promotes Epithelial-Mesenchymal Transition and Invasion of Bladder Cancer Cells via Activation of Nuclear Factor-kappaB	B7-H4	NF-kappaB
Whongsiri, P et	2018	29496693	0	LINE-1 ORF1 Protein Is Up-regulated by Reactive Oxygen Species and Associated with Bladder Urothelial Carcinoma Progression	LINE-1，ORF1p and 4-HNE	ROS
Wasen, C et al.	2018	29508172	0	Epidermal growth factor receptor function in the human urothelium	CK17, CK20, EGFr, pEGFr, laminin, occludin，ZO-1	EGFr signaling
Wang, Y et al.	2018	30570877	0	MiR-194-5p inhibits cell migration and invasion in bladder cancer by targeting E2F3	MiR-194-5p，E2F3	
Wang, Y et al.	2018	30563080	0	Obatoclax and Paclitaxel Synergistically Induce Apoptosis and Overcome Paclitaxel Resistance in Urothelial Cancer Cells	BH3，Bcl-2，Mcl-1， LC3-B，p62，PARP and caspase-3	
Wang, Y et al.	2018	30443191	0	PKM2 Inhibitor Shikonin Overcomes the Cisplatin Resistance in Bladder Cancer by Inducing Necroptosis	Bcl-2, PUMA, and Bax	
Wang, X et al.	2018	29328441	0	Ubenimex, an APN inhibitor, could serve as an antitumor drug in RT112 and 5637 cells by operating in an Aktassociated manner	APN, LC-3, Beclin 1, Akt/P-Akt, P62, C-caspase-3/P-caspase-3	Akt signaling pathway
Wang, W et al.	2018	29412153	0	Increased expression of GGN promotes tumorigenesis in bladder cancer and is correlated with poor prognosis	GGN，c-Myc, cyclin D1, Bcl-2, NFκB, IKK, IκB, bax, caspase3, p38MAPK, ERK1/2	NFκB-mediated apoptosis signaling
Wang, S et al.	2018	30344738	1	miR-124 regulates STAT3-mediated cell proliferation, migration and apoptosis in bladder cancer	miR-124，GADPH, STAT3，VEGFR，Bcl-2，Bcl-xl, MCL1, Mcl-1，Cyclin D1， MYC proto-oncogene, BHLH，cMyc	STAT3
Wang, M et al.	2018	29445179	0	Long noncoding RNA GAS5 promotes bladder cancer cells apoptosis through inhibiting EZH2 transcription	Long noncoding RNA GAS5，caspase-3，EZH2，E2F4， GST，miR-101	
Wang, K et al.	2018	30149755	0	Curcumin Suppresses microRNA-7641-Mediated Regulation of p16 Expression in Bladder Cancer	microRNA-76410， p16	
Wang, F et al.	2018	29284644	0	COX-2/sEH Dual Inhibitor PTUPB Potentiates the Antitumor Efficacy of Cisplatin	Ki-67, cleaved caspase-3 and CD31	AKT and ERK signaling pathways，MAPK/ERK and PI3K/AKT/mTOR signaling pathways
Wang, F et al.	2018	29316551	0	Chloroquine Enhances the Radiosensitivity of Bladder Cancer Cells by Inhibiting Autophagy and Activating Apoptosis.	LC3、P62、Bcl-2	
Wang, C et al.	2018	29545183	1	CXCL5 promotes mitomycin C resistance in non-muscle invasive bladder cancer by activating EMT and NF-kappaB pathway	CXCL5，CXCR2	EMT and NF-kappaB pathway

Table S1. The list of bladder cancer related articles either used or not used RT4.

Author	Year	PMID	using RT4 or not(0 represents No;1 represents Yes)	Title	Gene	Pathway
Wahafu, W et al	2018	29928321	0	Increased H(2)S and its synthases in urothelial cell carcinoma of the bladder, and enhanced cisplatin-induced apoptosis following H(2)S inhibition in EJ cells	CBS, CSE，MPST，p-Erk1/2, Bcl-2，Bcl-xL，Bax, Bad and cleaved PARP	Erk1/2 signalling pathway
Tsui, K. H et al.	2018	29239139	1	BTG2 is a tumor suppressor gene upregulated by p53 and PTEN in human bladder carcinoma cells	BTG2，p53，PTEN，pAKTs473, pAKTt308, pGSK3b, pmTOR, and pP70S6K	Akt signal pathway
Tsai, T. F et al.	2018	29379304	0	miR-99a-5p acts as tumor suppressor via targeting to mTOR and enhances RAD001-induced apoptosis in human urinary bladder urothelial carcinoma cells	miR-99a-5p，mTORC1 and mTORC2	AKT，mTOR
Tsai, K. W et al.	2018	30061227	0	Tight Junction Protein 1 Dysfunction Contributes to Cell Motility in Bladder Cancer	TJP1 and miR-455-5p	
Tong, H et al.	2018	30320898	1	Starvation-induced autophagy promotes the invasion and migration of human bladder cancer cells via TGF-beta1/Smad3-mediated epithelial-mesenchymal transition activation	LC3B－II and p62	TGF－β/Smad signaling pathway
Tian, D. W et al	2018	30535713	0	RAB38 promotes bladder cancer growth by promoting cell proliferation and motility	RAB38	
Tang, Z et al.	2018	30003752	0	Effects of human enhancer of filamentation 1 (HEF1) gene on the proliferation, invasion and metastasis of bladder cancer cells	HEF1	
Tang, Y et al.	2018	30143259	0	GnT-V promotes chemosensitivity to gemcitabine in bladder cancer cells through beta1,6 GlcNAc branch modification of human equilibrative nucleoside transporter 1	GnT-V， MGAT5，hENT1，caspase 3, 8 and 9	
Syring, I et al.	2018	30127891	0	The knockdown of the Mediator complex subunit MED15 restrains urothelial bladder cancer cells' malignancy	MED15	TGFβ，PI3K/mTOR signalling pathways
Suzuki, S et al.	2018	29501575	1	Orally administered nicotine effects on rat urinary bladder proliferation and carcinogenesis	Ki67 and pSTAT3	nAChR and STAT3 signaling
Sun, X et al.	2018	29463068	0	Benzidine Induces Epithelial-Mesenchymal Transition of Human Bladder Cancer Cells through Activation of ERK5 Pathway	ERK5，ERK1/2, JNK, p38	ERK1/2, JNK, p38，ERK5，MAPK/AP-1 Pathway
Sugita, S et al.	2018	29901113	0	HRAS as a potential therapeutic target of salirasib RAS inhibitor in bladder cancer	HRAS	
Shindo, T et al.	2018	29849953	0	Epigenetic silencing of miR-200b is associated with cisplatin resistance in bladder cancer	miR-200b，HAS2, ZEB1 and ZEB2	
Rosenkranz, A.	2018	30510514	1	Antitumor Activity of Auger Electron Emitter (111)In Delivered by Modular Nanotransporter for Treatment of Bladder Cancer With EGFR Overexpression	EGFR	EGFR
Roschger, C et a	2018	29245122	0	Reduction of cancer cell viability by synergistic combination of photodynamic treatment with the inhibition of the Id protein family	p27Kip1	
Rexin, P et al.	2018	29715095	0	The Immune Checkpoint Molecule CD200 Is Associated with Tumor Grading and Metastasis in Bladder Cancer	CD200	

Table S1. The list of bladder cancer related articles either used or not used RT4.

Author	Year	PMID	using RT4 or not(0 represents No;1 represents Yes)	Title	Gene	Pathway
Ramakrishnan, !	2018	30038946	0	Transcriptional changes associated with in vivo growth of muscle-invasive bladder cancer cell lines in nude mice	ABCB1，ABCG2，ADAMTS12，BMF，FLNC，MGST1，MAR2，CGB5，CGB8，EPAS1，PAPPA，DOCK4，FEZ1，FAT2，HCAR2，IFI44L，KRT17，KRT5，MMP10，TNC	
Pandey, S et al.	2018	29983877	1	Mutations of p53 decrease sensitivity to the anthracycline treatments in bladder cancer cells	PRIMA-1, AD 198, AD 312，TP53	p53
Nie, G et al.	2018	29351801	0	High expression of TMEM40 is associated with the malignant behavior and tumorigenesis in bladder cancer	TMEM40,p53,p21,CCND1,Caspase-3,Caspase-9 ,PARP ,c-MYC ,pEZ-M98	p53 signaling pathway
Naiki, T et al.	2018	29662611	1	GPX2 promotes development of bladder cancer with squamous cell differentiation through the control of apoptosis	GPX2,Ki67,caspase 3 or 7	oxidative stress
Morita, K et al.	2018	30248959	0	NACC1, as a Target of MicroRNA-331-3p, Regulates Cell Proliferation in Urothelial Carcinoma Cells	NACC1, miR-331-3p	
Miki, M et al.	2018	30139660	1	Predicting the tumorigenic phenotype of human bladder cancer cells by combining with fetal rat mesenchyme	Ki-67 and p63	
Michalska, M et	2018	29491061	0	Impact of Methadone on Cisplatin Treatment of Bladder Cancer Cells	PARP,caspase-3,BCL-2,MCL-1,XIAP	
Matsunaga, W e	2018	29599318	0	Lentiviral Vector-mediated Gene Transfer in Human Bladder Cancer Cell Lines	p53, p16, and PTEN	
Mao, M. H et al	2018	30257321	0	Additive antitumor effect of arsenic trioxide combined with intravesical bacillus Calmette-Guerin immunotherapy against bladder cancer through blockade of the IER3/Nrf2 pathway	IER3, Nrf2, NQO1, IL-6 and IL-8	IER3/Nrf2 pathway
Mao, G et al.	2018	30365074	0	Long term exposure to gammarays induces radioresistance and enhances the migration ability of bladder cancer cells	STAT3	STAT3
Maj, M et al.	2018	30310111	0	The interplay between adipose-derived stem cells and bladder cancer cells	p-AKT,p-ERK1/2 ,AKT/ERK/p70 S6K	AKT/ERK/p70 S6K
Ma, W et al.	2018	30253078	0	Targeting immunotherapy for bladder cancer using anti-CD3x B7-H3 bispecific antibody	B7-H3	
Ma, J et al.	2018	29328392	0	Targeting bladder cancer using activated T cells armed with bispecific antibodies	EGFR or HER2	EGFR
Lv, X. Y et al.	2018	29115412	1	Knockdown of DUXAP10 inhibits proliferation and promotes apoptosis in bladder cancer cells via PI3K/Akt/mTOR signaling pathway	DUXAP10，Bcl-xL, cyclin D，CDK4，Bad, cleaved caspase-3, cleaved caspase-9, and p27	PI3K/Akt/mTOR signaling pathway
Luo, K. W et al.	2018	29552308	0	EGCG inhibited bladder cancer T24 and 5637 cell proliferation and migration via PI3K/AKT pathway	EGCG，Caspase-3, PARP, NF-κB p65, p-NF-κB p65, PI3K, p-PI3K, AKT, p-AKT	PI3K/AKT pathway
Luo, J et al.	2018	30475758	0	Targeted regulation by ROCK2 on bladder carcinoma via Wnt signaling under hypoxia.	ROCK2、HIF-1α、E-cadherin、Wnt4、β-catenin	ROCK2-Wnt4
Luan, T et al.	2018	30463687	1	MicroRNA-98 promotes drug resistance and regulates mitochondrial dynamics by targeting LASS2 in bladder cancer cells.	LASS2、cyclin D1、p-Drp1、Drp1、Fis1	MicroRNA-98—LASS2

Table S1. The list of bladder cancer related articles either used or not used RT4.

Author	Year	PMID	using RT4 or not(0 represents No;1 represents Yes)	Title	Gene	Pathway
Liu, Z et al.	2018	30310053	0	Long noncoding RNA neuroblastoma-associated transcript 1 gene inhibits malignant cellular phenotypes of bladder cancer through miR-21/SOCS6 axis	NBAT1、miR-21、SOCS6、cyclin D1	NBAT1-miR-21-SOCS6
Li Y et al.	2018	30249892	0	Overexpression of Tat-interacting protein 30 inhibits the proliferation, migration, invasion and promotes apoptosis in bladder cancer cells	TIP30、EGFR、Akt、Bcl-2、cyclin D、cyclin E、MMP2、MMP6、MMP9、bax、caspase3	
Liu Y et al	2018	29964053	0	High expression of enhancer RNA MARC1 or its activation by DHT is associated with the malignant behavior in bladder cancer.	eMARC1	
Liu X et al.	2018	29422113	0	Puerarin Inhibits Proliferation and Induces Apoptosis by Upregulation of miR-16 in Bladder Cancer Cell Line T24.	miR-16、p65、IkBa、COX-2、cyclin D1、Bcl-2、Bax、caspase 3、caspase 9	miR-16-NF-κB-COX-2
Liu S et al.	2018	29512774	0	Knockdown of tyrosine 3-monooxygenase/tryptophan 5-monooxygenase activation protein zeta (YWHAZ) enhances tumorigenesis both in vivo and in vitro in bladder cancer.	YWHAZ	
Liu J et al.	2018	29328435	0	miR-214 reduces cisplatin resistance by targeting netrin-1 in bladder cancer cells.	miR-214、netrin-1、 caspase-3、PARP、AKT	miR-214-netrin-1
Liu GM et al.	2018	30327690	0	Nesfatin-1/Nucleobindin-2 Is a Potent Prognostic Marker and Enhances Cell Proliferation, Migration, and Invasion in Bladder Cancer.	NUCB2、Ki67、PCNA、MMP2、MMP9	
Zhu F et al.	2018	29231261	1	LncRNA AWPPH inhibits SMAD4 via EZH2 to regulate bladder cancer progression.	AWPPH、EZH2、SMAD4、Bax、Bcl－2、LC3、Beclin-1、H3K27me3	AWPPH- EZH2- H3K27me3-SMAD4
Lin W et al.	2018	29725263	0	Glaucocalyxin A induces G2/M cell cycle arrest and apoptosis through the PI3K/Akt pathway in human bladder cancer cells.	P21、Cdc25C、p85、Akt、Bcl-2、CDK1、Cyclin B1、PTEN、Bax、Caspase-3、Caspase-9、PARP	
Liao X et al.	2018	29226855	0	Knockdown of long noncoding RNA FGFR3- AS1 induces cell proliferation inhibition, apoptosis and motility reduction in bladder cancer.	FGFR3- AS1、	
Bourn J et al.	2018	29942116	0	Cyclooxygenase inhibitors potentiate receptor tyrosine kinase therapies in bladder cancer cells in vitro.	Kit、PDGFRα、ERK1/2、Akt (Ser473)、Akt1/2/3、COX-1、COX-2、NF-κB、caspase-3、caspase-7、PARP	
Li Z et al.	2018	29434982	0	Genipin inhibits the growth of human bladder cancer cells via inactivation of PI3K/Akt signaling.	PI3K、Akt	PI3K-Akt
Li Z et al.	2018	29883941	1	Oncogene miR-187-5p is associated with cellular proliferation, migration, invasion, apoptosis and an increased risk of recurrence in bladder cancer.	miR-187-5p	
Li T et al.	2018	30127910	0	Anticancer effect of salidroside reduces viability through autophagy/PI3K/Akt and MMP-9 signaling pathways in human bladder cancer cells.	PI3K、Akt、MMP9、Bax、Bcl-2	autophagy-PI3K-Akt-Bcl-2

Table S1. The list of bladder cancer related articles either used or not used RT4.

Author	Year	PMID	using RT4 or not(0 represents No;1 represents Yes)	Title	Gene	Pathway
Li J et al.	2018	30042171	0	Knockdown of long non-coding RNA linc00511 suppresses proliferation and promotes apoptosis of bladder cancer cells via suppressing Wnt/β-catenin signaling pathway.	Caspase-3、Caspase-9、PARP、Rb、CDK4、Cyclin D1、P21、P18、myc、β-catenin、linc00511、miR-15a-3p	linc00511-miR-15a-3p；linc00511-Wnt-β-catenin
Li CF et al.	2018	29671787	0	Proteomic Identification of the Galectin-1-Involved Molecular Pathways in Urinary Bladder Urothelial Carcinoma.	TOLLIP、FABP 4、GS、SNX9、TRXR1、UBE2K、STMN1、AKR1A1	
Li C et al.	2018	29749526	1	Human HLA-F adjacent transcript 10 promotes the formation of cancer initiating cells and cisplatin resistance in bladder cancer.	FAT10、Vimentin、E-cadherin、N-cadherin	
Li C et al.	2018	29620261	0	Upregulation of E-cadherin expression mediated by a novel dsRNA suppresses	E-cadherin、Cyclin D1、MMP2、MYC、miR-373、dsEcad-346	dsEcad-346&miR-373- E-cadherin-β -catenin-TCF
Li B et al.	2018	29552157	0	Vasculogenic mimicry in bladder cancer and its association with the aberrant expression of ZEB1.	CD34、PAS、 ZEB1	
Kim WT et al.	2018	29595070	0	The Anticancer Effects of Garlic Extracts on Bladder Cancer Compared to Cisplatin: A Common Mechanism of Action via Centromere Protein M.	CENPM	
Kim D et al.	2018	29717739	0	Suppression of T24 human bladder cancer cells by ROS from locally delivered hematoporphyrin-containing polyurethane films.	Caspase-3、Caspase-9、Caspase-7、Bcl2、Bax	
Kim CJ et al.	2018	29115414	0	Anti-oncogenic activities of cyclin D1b siRNA on human bladder cancer cells via induction of apoptosis and suppression of cancer cell stemness and invasiveness.	Cyclin D1、Rb、CD44、Vimentin、E-cadherin、N-cadherin	
Khandelwal M (	2018	29368096	0	Decitabine augments cytotoxicity of cisplatin and doxorubicin to bladder cancer cells by activating hippo pathway through RASSF1A.	RASSF1A、MST、YAP、AREG、CTGF、CYR61	RASSF1A-hippo pathway
Jiang X et al.	2018	29665744	0	Expression and Functions of Formyl Peptide Receptor 1 in Drug-Resistant Bladder Cancer.	FPR1、MRP1、LRP、P-gp、Bcl 2、GST、JAK2、STAT3、PI3K、AKT、ERK-1/2、PI3K	FPR1- JAK2-STAT3；FPR1-ERK1/2
Jiang L et al.	2018	30257376	0	Baicalein inhibits proliferation and migration of bladder cancer cell line T24 by down-regulation of microRNA-106.	P16、P21、Cyclin D、Caspase-3、Caspase-9、Bcl2、Bax、MMP2、MMP9、miR-106、JNK、ERK、MEK	miR-106-JNK&MEK/ERK
Jiang K et al.	2018	29375709	0	Puerarin inhibits bladder cancer cell proliferation through the mTOR/p70S6K signaling pathway.	mTOR、 p70S6K	mTOR-p70S6K
Huang X et al.	2018	29549123	0	Wnt7a activates canonical Wnt signaling, promotes bladder cancer cell invasion, and is suppressed by miR-370-3p.	miR-370-3p、Wnt7a、MMP1、MMP9、MMP10、 β-catenin、Vimentin、E-cadherin、N-cadherin、Zeb1、Twist	miR-370-3p-Wnt7a-Wnt
Huang L et al.	2018	29581781	0	LASS2 regulates invasion and chemoresistance via ERK/Drp1 modulated mitochondrial dynamics in bladder cancer cells.	LASS2、ERK、Drp1、Fis1、ERK	LASS2-ERK-Drp1
Huang H et al.	2018	30127995	0	Bufalin induced apoptosis of bladder carcinoma cells through the inactivation of Na+K+-ATPase.	Caspase-3、Bcl 2、ATPα1、ATPα3	

Table S1. The list of bladder cancer related articles either used or not used RT4.

Author	Year	PMID	using RT4 or not(0 represents No;1 represents Yes)	Title	Gene	Pathway
Hori S et al.	2018	29731962	0	Gamma-Klotho exhibits multiple roles in tumor growth of human bladder cancer.	KLγ、Ki67、TUNEL、Vimentin、E-cadherin、N-cadherin、AKT、ERK	
Ho JN et al.	2018	29285203	0	Anticancer effect of S-allyl-L-cysteine via induction of apoptosis in human bladder cancer cells.	Caspase-3、Caspase-8、Caspase-9、RAPR、Cytochorme c、Bcl 2、Bax、AKT、Cyclin A、Cyclin B1、Cyclin D1、Cyclin E1	
Hänze J et al.	2018	29966976	0	Effects of multi and selective targeted tyrosine kinase inhibitors on function and signaling of different bladder cancer cells.	ACTB 、BAX、BCL2、CCND1、CDH1、CDH2、CDKN1A 、CDKN1B、CJUN、E2F3、EGFR1、FGF1、FGF2、FGFR1、FGFR2、FGFR3、FGFR4、FLT1 、FLT3 、FRS2 、HBEGF、HER2 、HER3、KIT、MET 、MKI67 、MYC、PDGFRA、PLAU、PTGS2、RAC1、TBP、VEGFA、VIM	
Guo Y et al.	2018	30349370	0	Non-coding RNA NEAT1/miR-214-3p contribute to doxorubicin resistance of urothelial bladder cancer preliminary through the Wnt/β-catenin pathway.	NEAT1、miR-214-3p、Wnt、β-catenin 、P-gp、Axin2、GSK3B	NEAT1-miR-214-3p-Wnt-β-catenin
Guo X et al.	2018	30195772	0	ISO, via Upregulating MiR-137 Transcription, Inhibits GSK3β-HSP70-MMP-2 Axis, Resulting in Attenuating Urothelial Cancer Invasion.	ISO、MiR-137、GSK3β、WAVE3、RhoGDIβ、MMP-2、mTOR	ISO- MiR-137-GSK3β-HSP70-MMP-2
Guo P et al.	2018	29321088	0	Upregulation of Long Noncoding RNA TUG1 Promotes Bladder Cancer Cell Proliferation, Migration, and Invasion by Inhibiting miR-29c.	TUG1、 miR-29c	
Guo L et al.	2018	30127997	0	CREB1, a direct target of miR-122, promotes cell proliferation and invasion in bladder cancer.	CREB1、miR-122	miR-122-CREB1
Guo J et al.	2018	29659560	0	Androgen Receptor Is Inactivated and Degraded in Bladder Cancer Cells by Phenyl Glucosamine via miR-449a Restoration.	Cyclin D1、AR、 miR-449a	miR-449a-AR
Gong X et al.	2018	30032159	1	Rab11 Functions as an Oncoprotein via Nuclear Factor kappa B (NF-κB) Signaling Pathway in Human Bladder Carcinoma.	Rab11、Cyclin D1、Cyclin E、MMP9、IκB	Rab11-NF-κB-MMP9
Gao ZG et al.	2018	29225130	0	On the G protein-coupling selectivity of the native A2B adenosine receptor.	AR、ERK1/2	AR-ERK1/2
Gao Q et al.	2018	30275714	0	Lentivirus-mediated shRNA targeting MUTYH inhibits malignant phenotypes of bladder cancer SW780 cells.	MUTYH、caspase-3	
Gao L et al.	2018	30556853	0	High-glucose promotes proliferation of human bladder cancer T24 cells by activating Wnt/β-catenin signaling pathway.	Wnt、β-catenin	Wnt-β-catenin
Feng Z et al.	2018	29541223	0	Long non-coding RNA HNF1A-AS1 promotes cell viability and migration in human bladder cancer.	HNF1A-AS1	
Feng SQ et al.	2018	29940769	0	Up-regulation of LncRNA MEG3 inhibits cell migration and invasion and enhances cisplatin chemosensitivity in bladder cancer cells	MEG3、MMP2、MMP9、Bcl 2、Bax、Caspase-3、P53	

Table S1. The list of bladder cancer related articles either used or not used RT4.

Author	Year	PMID	using RT4 or not(0 represents No;1 represents Yes)	Title	Gene	Pathway
Feng F et al.	2018	30132983	1	Long noncoding RNA SNHG16 contributes to the development of bladder cancer via regulating miR-98/STAT3/Wnt/β-catenin pathway axis.	SNHG16、miR-98、STAT3、Bcl 2、Bax、Caspase-3、Caspase-9、Vimentin、E-cadherin、N-cadherin、Snail、p27Kip1、 Wnt1、 c‑Myc、cyclin‑D1	SNHG16-miR-98-STAT3-Wnt-β-catenin
Fang T et al.	2018	29769742	0	Modified citrus pectin inhibited bladder tumor growth through downregulation of galectin-3.	Cyclin B1、Cdc 2、Caspase-3、PARP 、AKT、Bad、S6RP、ERK	
Dudek AM et al	2018	29856124	0	LINC00857 expression predicts and mediates the response to platinum-based chemotherapy in muscle-invasive bladder cancer.	LINC00857	
Dong ZC et al.	2018	29863246	1	Target inhibition on GSK-3β by miR-9 to modulate proliferation and apoptosis of bladder cancer cells.	miR-9、GSK-3β、β-catenin	miR-9-GSK-3β
Ding M et al.	2018	30252203	0	Oestrogen promotes tumorigenesis of bladder cancer by inducing the enhancer RNA-eGREB1.	eGREB1	
Dietrich F et al.	2018	29305710	0	Ecto-5'-nucleotidase/CD73 contributes to the radiosensitivity of T24 human bladder cancer cell line.	ecto-5′ NT/CD73	
D'Aloia A et al.	2018	29208460	0	RalGPS2 is involved in tunneling nanotubes formation in 5637 bladder cancer cells.	RalA、RalGPS2、AKT、 LST1	
da Silva GN et a	2018	28577130	1	Gemcitabine/Cisplatin Treatment Induces Concomitant SERTAD1, CDKN2B and GADD45A Modulation and Cellular Changes in Bladder Cancer Cells Regardless of the Site of TP53 Mutation.	RB1、BIRC5、SERTAD1、CDKN1A 、CDKN2B、GADD45A、RAD51、CCNF、NBN、TP53、CCNT1、MCM5、CCNE1、MAD2L2、CCNT2、CHEK 1、BRCA2、MCM2 、MCM4、DIRAS3、GTF2H1、MCM3	
Chu M et al.	2018	29367676	1	Inhibition of angiogenesis by leflunomide via targeting the soluble ephrin-A1/EphA2 system in bladder cancer.	sEphrin-A1、EphA2	sEphrin-A1-EphA2
Yuwen Chen	2018	29891980	1	(-)-Gochnatiolide B, synthesized from dehydrocostuslactone, exhibits potent anti-bladder cancer activity in vitro and in vivo	PARP and caspase-3、cytochrome c 、Bim、 Bcl-xl, Mcl-1, XIAP and survivin, p21 and p27 、cyclin D1、SKP2	
Chen X	2018	30032142	0	MicroRNA-374a Inhibits Aggressive Tumor Biological Behavior in Bladder Carcinoma by Suppressing Wnt/beta-Catenin Signaling	miR-374a、PTEN, WIF1, and WNT5A. PTEN 、SOX2, CD133, and ABCG2,	Wnt/β-catenin
Jun-Feng Chen	2018	29725403	0	RON is overexpressed in bladder cancer and contributes to tumorigenic phenotypes in 5637 cells	RON 、cyclin D1, cyclin D3 and CDK4 、p27 and p21	the phosphoinositide 3-kinase (PI3K)-Akt and MAPK pathways
Jin-Bo Chen	2018	30588263	0	Glucocorticoid-Inducible Kinase 2 Promotes Bladder Cancer Cell Proliferation, Migration and Invasion by Enhancing beta-catenin/c-Myc Signaling Pathway	SGK2、β-catenin/c-Myc	β-catenin/c-Myc
Dongmei Chen	2018	29658613	0	Detection of survivin expression in bladder cancer and renal cell carcinoma using specific monoclonal antibodies	survivin	
Dong Chen	2018	29725433	0	Cinobufacini promotes apoptosis of bladder cancer cells by influencing the expression of autophagy-related genes	Bax、Bcl-2、p62、LC3、Cleaved caspase-3	

Table S1. The list of bladder cancer related articles either used or not used RT4.

Author	Year	PMID	using RT4 or not(0 represents No;1 represents Yes)	Title	Gene	Pathway
Ching-Ping Cha	2018	29702555	0	Deciphering the Molecular Mechanism Underlying the Inhibitory Efficacy of Taiwanese Local Pomegranate Peels against Urinary Bladder Urothelial Carcinoma	pro-/cleaved caspase-3、 pro-/cleaved caspase-8, DR4 and DR5、 pro-/cleaved caspase-9, Bax and Bcl-2 、Bip, VCP and pro- caspase-12	
J. Bosschieter	2018	30142219	0	A protocol for urine collection and storage prior to DNA methylation analysis	RASSF1A、ACTB	
Christian Bolenz	2018	29430508	0	Decreased Invasion of Urothelial Carcinoma of the Bladder by Inhibition of Matrix-Metalloproteinase 7	MMP1、MMP2、MMP9、MMP7	
Simone Bersini	2018	30546831	0	A combined microfluidic-transcriptomic approach to characterize the extravasation potential of cancer cells	LOX、MMP2、PTX3、NID2、SPOCK1、ADAM、ADAMTS	
Simon C. Baker	2018	29323757	0	Differentiation-associated urothelial cytochrome P450 oxidoreductase predicates the xenobiotic-metabolizing activity of "luminal" muscle-invasive bladder cancers	AHR、CYP1A1、CYP1B1、EROD 、POR	
Fahmideh Bagr	2018	29480996	0	Expression of Inhibitor of Apoptosis Gene Family Members in Bladder Cancer Tissues and the 5637 Tumor Cell Line	BIRC1 (NAIP (, BIRC2 (IAP1 (, BIRC3) IAP2 (, BIRC5) Survivian (, BIRC6 (Apollon/Bruce (, BIRC7 (ML-IAP (and BIRC8) ILP-2 (were up-regulated and expression rate of BIRC4) XIAP	
Seiji Asai	2018	29391886	0	Silencing of ECHDC1 inhibits growth of gemcitabine-resistant bladder cancer cells	ECHDC1、p27	
Akihiro Asai	2018	28952025	1	Pathological significance and prognostic significance of FES expression in bladder cancer vary according to tumor grade	FES	
C. Andolino	2018	29802537	0	Drug-induced keratin 9 interaction with Hsp70 in bladder cancer cells	Keratin 9 Hsp70	
QingAn	2018	29674277	0	Long noncoding RNA FOXD2-AS1 accelerates the gemcitabine-resistance of bladder cancer by sponging miR-143	LncRNA FOXD2-AS1 ABCC3 miR-143	
Cao R et al.	2018	29175458	1	TM4SF1 regulates apoptosis, cell cycle and ROS metabolism via the PPARγ-SIRT1 feedback loop in human bladder cancer cells.	TM4SF1、Bax、Bcl-xl、Caspase3、Caspase6、Caspase7、Caspase9、SIRT1、PPARγ、FOXO3a、Catales、SOD2、CCNA1/2、CCND1、CCDK2 、CDK4	PPARγ-SIRT1
Cao R et al.	2018	29175458	1	TM4SF1 regulates apoptosis, cell cycle and ROS metabolism via the PPARγ-SIRT1 feedback loop in human bladder cancer cells.	TM4SF1、Bax、Bcl-xl、Caspase3、Caspase6、Caspase7、Caspase9、SIRT1、PPARγ、FOXO3a、Catales、SOD2、CCNA1/2、CCND1、CCDK2 、CDK4	PPARγ-SIRT1
Zhuang ChL et	2017	29084575	0	Synthetic regulatory RNAs selectively suppress the progression of bladder cancer	GAL4、 UAS、hTERT、MYC、EGFP、 CMV、	GAL4-VP64

Table S1. The list of bladder cancer related articles either used or not used RT4.

Author	Year	PMID	using RT4 or not(0 represents No;1 represents Yes)	Title	Gene	Pathway
Qiang Zhou et a	2017	28435431	0	DIOSCIN INDUCES DEMETHYLATION OF DAPK-1 AND RASSF-1 GENES VIA THE ANTIOXIDANT CAPACITY,RESULTING IN APOPTOSIS OF BLADDER CANCER T24 CELLS	DAPK-1 、RASSF-1α	
Yi Zhi et al.	2017	28222669	1	Downregulated XPA promotes carcinogenesis of bladder cancer via impairment of DNA repair	XPA、 CDK1、CyclinB、CDK2、CyclinE、	CDK1/Cyclin B and CDK2/Cyclin E
Shuai Zhen et al	2017	28038452	0	Inhibition of long non-coding RNA UCA1 by CRISPR/Cas9 attenuated malignant phenotypes of bladder cancer	Cas9、UCA1-1、UCA1-2、UCA1-3、UCA1-4、UCA1-5、UCA1-6、UCA1-7、UCA1-8、、MMP-2、MMP-9、Bax,Bcl-2	
	2017	29048677	0	MicroRNA-154 as a prognostic factor in bladder cancer inhibits cellular malignancy by targeting RSF1 and RUNX2	has-miR-154、RSF1、RUNX2、U6、	miR-154-RSF1/RUNX2，NF- κ B
Zhe Zhang et al	2017	27878946	0	Targeted inhibition of Polo-like kinase 1 by a novel small-molecule inhibitor induces mitotic catastrophe and apoptosis in human bladder cancer cells	PLK1、 PARP、caspase-3、 BubR1 、MPM-2、CDC2、Wee1、	PLK1/Wee1/CDC2
	2017	29246203	0	Comprehensive analysis of differentially expressed genes associated with PLK1 in bladder cancer	PLK1、BUB1B, CCNB1,CDC25A, FBXO5, NDC80、PI3K、PVDF	PLK1-BUB1B, CCNB1, CDC25A, FBXO5, NDC80
	2017	29187903	0	miR34a/GOLPH3 Axis abrogates Urothelial Bladder Cancer Chemoresistance via Reduced Cancer Stemness	CD44,ALDH1A1, Sox2, KLF4、GOLPH3、CK5, CK14, CD44,	miR34a-GOLPH3
ZHANG Kai et a	2017	28502307	0	Knock-down of DAB2 interacting protein (DAB2IP) promotes proliferation and inhibits apoptosis of bladder cancer cells	DAB2IP、PARP、 c-caspase-3、 Bcl-2 、Mcl-1	
HUI-HUI ZHAN	2017	29142609	0	Role of 5-Aza-CdR in mitomycin-C chemosensitivity of T24 bladder cancer cells	5-Aza-CdR、Beclin1, p62 、ATG5	
Hui Zhang et al.	2017	27878359	0	Long noncoding RNA GAS5 inhibits malignant proliferation and chemotherapy resistance to doxorubicin in bladder transitional cell carcinoma	SUMO1P3、LOC572558、GAS5、Bcl-2	AKT-MDM2-p53
Shuxiong Zeng	2017	28600503	0	Transcriptome sequencing identifies ANLN as a promising prognostic biomarker in bladder urothelial carcinoma	ANLN、CyclinB1、CyclinA1、CyclinD1、CyclinE1、RhoA、	PI3K/Akt
SHEAu-YuN Y	2017	28498480	0	Miconazole induces apoptosis via the death receptor 5-dependent and mitochondrial-mediated pathways in human bladder cancer cells	p21、p27, cyclin E1,CDK2、CDK4、p53、CyclinD1、CyclinE1、CDK2、PARP、Caspase3、Caspase8、Caspase9	
Hejia Yuan et a	2017	28631286	0	Knockdown of mediator subunit Med19 suppresses bladder cancer cell proliferation and migration by downregulating Wnt/b-catenin signalling pathway	Med19、Wnt2 、E-cadherin 、Gsk3b 、Cyclin-D1、MMP-9 、β-catenin	Wnt/b-catenin
GUO-QIANG Y	2017	27922685	0	5-bromo-3-(3-hydroxyprop-1-ynyl)-2H-pyran-2-one induces apoptosis in T24 human bladder cancer cells through mitochondria-dependent signaling pathways	AIF、BAX、Bcl-2 、PARP、Caspase3、Caspase8、Caspase9、CyclinE、p21、p53、Cdc25c、Endo-g	
YU Gan et al.	2017	29180330	0	Role of lncRNA PTENP1 in tumorigenesis and progression of bladder cancer and the molecular mechanism	PTENP1	

Table S1. The list of bladder cancer related articles either used or not used RT4.

Author	Year	PMID	using RT4 or not(0 represents No;1 represents Yes)	Title	Gene	Pathway
Hubin Yin et al.	2017	29069735	0	HMGB1-mediated autophagy attenuates gemcitabine-induced apoptosis in bladder cancer cells involving JNK and ERK activation	HMGB1、p62、LC3、PARP、Caspase3、JNK、AKT、ERK、Bcl-2	HMGB1-JNK and ERK
H. Yin et al.	2017	28258492	0	SOX10 is over-expressed in bladder cancer and contributes to the malignant bladder cancer cell behaviors	SOX10、β-catenin、Met	Wnt/b-catenin
Yang Yang et a	2017	28178690	0	Differential sensitivities of bladder cancer cell lines to resveratol are unrelated to its metabolic profile	SULT1A1	
Yu Yang et al.	2017	28647190	0	Matrine inhibits bladder cancer cell growth and invasion in vitro through PI3K/AKT signaling pathway: An experimental study	p-PI3K, p-AKT, MMP2 ， MMP9， p16, p21 ， p27	PI3K/AKT
Xiaoping Yang	2017	28824948	0	The Antineoplastic Activity of Photothermal Ablative Therapy with Targeted Gold Nanorods in an Orthotopic Urinary Bladder Cancer Model	EGFR，cleaved-caspase3	
Zhonghua Yi et	2017	28427129	0	The role of FOXO3a-Bim signaling in triptolide induced bladder cancer T24 cells apoptosis	p-AKT，FOXO3a，cleaved-caspase3，BAX,Bim	FOXO3a-Bim
L. YANG et al.	2017	28895409	0	Long noncoding RNA ASAP1-IT1 promotes cancer stemness and predicts a poor prognosis in patients with bladder cancer	ASAP1-IT1,CD44	
Jiangeng Yang	2017	28978151	0	Hsa-miR-429 promotes bladder cancer cell proliferation via inhibiting CDKN2B	Hsa-miR-429 ，CDKN2B	Hsa-miR-429-CDKN2B
Lei Yan et al.	2017	28521108	0	MiR-301b promotes the proliferation, mobility, and epithelial-to-mesenchymal transition of bladder cancer cells by targeting EGR1	MiR-301b，EGR1，β-catenin，Met，N-cadherin, MMP2, MMP9，vimentin	MiR-301b-EGR1
Mei Xue et al.	2017	28841829	0	Hypoxic exosomes facilitate bladder tumor growth and development through transferring long non-coding RNA-UCA1	lncRNA-UCA1，E-cadherin，MMP9，vimentin，Ki67	
Xue-lian Xu et	2017	29151930	0	Overexpression of PTK6 predicts poor prognosis in bladder cancer patients	PTK6	
Xin Xu et al.	2017	29254226	0	CRISPR-ON-Mediated KLF4 overexpression inhibits the proliferation, migration and invasion of urothelial bladder cancer in vitro and in vivo	KLF4，AKT,p21, CCND1, RB, p-RB,E-cadherin,Fibronectin,Snail,Slug	AKT/p21
YANG XIONG	2017	28534984	0	CCL21/CCR7 interaction promotes cellular migration and invasion via modulation of the MEK/ERK1/2 signaling pathway and correlates with lymphatic metastatic spread and poor prognosis in urinary bladder cancer	CCL21/CCR7，AKT，ERK，	MEK/ERK1/2
Dalong Xie et a	2017	29179467	0	Knockdown of long non-coding RNA Taurine Up-Regulated 1 inhibited doxorubicin resistance of bladder urothelial carcinoma via Wnt/β-catenin pathway	TUG1,β-catenin,	Wnt/β-catenin
Yiying Wu et al	2017	28139790	0	Formononetin inhibits human bladder cancer cell proliferation and invasiveness via regulation of miR-21 and PTEN	miR-21，PTEN，Akt，Bcl-2, Bcl-x，MMP-2，MMP-9	PI3K/Akt
Xiaoqiang Wu	2017	28992472	0	LncRNA ZEB2-AS1 promotes bladder cancer cell proliferation and inhibits apoptosis by regulating miR-27b	ZEB2-AS1 ， miR-27b，	ZEB2-AS1-miR-27b
Joana Liberal et	2017	28631098	0	Urolithins impair cell proliferation, arrest the cell cycle and induce apoptosis in UMUC3 bladder cancer cells	AKT，ERK，SAPK，p38	PI3K/Akt and MAPK

Table S1. The list of bladder cancer related articles either used or not used RT4.

Author	Year	PMID	using RT4 or not(0 represents No;1 represents Yes)	Title	Gene	Pathway
DEYAO WU et	2017	28498468	0	MicroRNA-379-5p plays a tumor-suppressive role in human bladder cancer growth and metastasis by directly targeting MDM2	miR-379-5p，MDM2，	miR-379-5p/MDM2
ZHIYONG WEI	2017	28656286	0	MicroRNA-497 upregulation inhibits cell invasion and metastasis in T24 and BIU-87 bladder cancer cells	MicroRNA-497，E-cadherin，Vimentin，α -SMA ，U6，	MicroRNA-497- α -SMA
SHENG WANG	2017	28677729	0	Centromere protein U is a potential target for gene therapy of human bladder cancer	CENPU， ILB, CXCL8,RAC1 ，IL1A，CCND3，IL6 ，TNFRSF11B ，PTGS2 ，FN1，MAD2L1，	CENPU- HMGB1
Jintao Wang et a	2017	28946549	0	Long non-coding RNA HULC promotes bladder cancer cells proliferation but inhibits apoptosis via regulation of ZIC2 and PI3K/AKT signaling pathway	HULC，Cyclin A, Cyclin D1, Cyclin E, Bcl-2, ZIC2 ,AKT , (P)-AKT , PI3K and P-PI3K	PI3K/AKT
HAIFENG WAN	2017	28356943	0	LASS2 inhibits growth and invasion of bladder cancer by regulating ATPase activity	LASS2，ATP6L	LASS2-ATP6L
HENGBING W.	2017	28123579	0	miR-143 inhibits bladder cancer cell proliferation and enhances their sensitivity to gemcitabine by repressing IGF-1R signaling	miR-143,IGF-1R,U6,IGF-1R, p-Akt , Akt, p-ERK ,ERK	IGF-1R
Fei Wang et al.	2017	29190896	1	Cofilin 1 promotes bladder cancer and is regulated by TCF7L2	TCF7L2,ADF,Cofilin 1,CCK-8,MMP-9,PDK1	TCF7L2-Cofilin 1
Dengdian Wang	2017	28870814	0	Curcumin inhibits bladder cancer stem cells by suppressing Sonic Hedgehog pathway	CD44,CD133, ALDH1-A1, OCT-4 ,Nanog, Bax, Cleaved-Caspase 3, Cleaved-Caspase 8, and Cleaved-Caspase 9	Sonic Hedgehog(Shh)
Chongshan Wan	2017	28042037	0	Lycorine induces apoptosis of bladder cancer T24 cells by inhibiting phospho-Akt and activating the intrinsic apoptotic cascade	Akt, p-Akt, caspase-3, cleaved caspase-3, and PTEN,Bcl-2,	PI3K/AKT/mTOR
Nisha Verma et	2017	28851999	1	Benzo[a]pyrene-induced metabolic shift from glycolysis to pentose phosphate pathway in the human bladder cancer cell line RT4	Nrf2,HIF-1α,NFκB,ARE	Nrf2-ARE
Venkatrao Vant	2017	29221154	0	Expression of ganglioside GD2, reprogram the lipid metabolism and EMT phenotype in bladder cancer	E-cadherin，Vimentin,GD2	PI3-kinase
STEFAN VALL	2017	29113179	0	Blocking integrin β 1 decreases adhesion in chemoresistant urothelial cancer cell lines	integrin β 1	
STEFAN VALL	2017	28599410	0	Resistance to nanoparticle albumin-bound paclitaxel is mediated by ABCB1 in urothelial cancer cells	ABCB1，pAkt，ERK1/2 ，MET，	ABCB1-pAkt
YUJIRO TSUJI	2017	28849140	0	STAT3 inhibition by WP1066 suppresses the growth and invasiveness of bladder cancer cells	STAT3，ERK，Bcl-2，Cleaved-Caspase 3，PARP，MMP-9,MMP-2	STAT3-ERK
Josiane Weber T	2017	28750358	0	Antitumor potential of 1-thiocarbamoyl-3,5-diaryl-4,5-dihydro-1H-pyrazoles in human bladder cancer cells	STAT, PI3K-AKT,ERK and PKC	FGFR3/CNND1,E2F3/RB1
SHUIXIANG T.	2017	28138703	0	ATM participates in the regulation of viability and cell cycle via ellipticine in bladder cancer	ATM,Chk1,Cdc25C,Cdk1,	ATM-Chk1-Cdc25C-Cdk1
Min Tang et al.	2017	28229220	0	Overexpression of HepaCAM inhibits bladder cancer cell proliferation and viability through the AKT/FoxO pathway	HepaCAM,AKT/FoxO. CDK2，SC79 ，PCNA	AKT/FoxO

Table S1. The list of bladder cancer related articles either used or not used RT4.

Author	Year	PMID	using RT4 or not(0 represents No;1 represents Yes)	Title	Gene	Pathway
Ping Wu et al.	2017	28948276	0	Apoptosis triggered by isoquercitrin in bladder cancer cells by activating the AMPK-activated protein kinase pathway	Fas， FADD，Caspase-3, Caspase-8, Caspase-9， p-AMPK, p53, p27, p38，p21	PI3K/AKT/mTOR
Tomoaki Takai	2017	28106737	0	A Novel Combination RNAi toward Warburg Effect by Replacement with miR-145 and Silencing of PTBP1 Induces Apoptotic Cell Death in Bladder Cancer Cells	miR-145，PTBP1，FSCN1,PARP,PKM2	c-Myc/PTBP1/PKMs
Xin Sun et al.	2017	28012925	0	Cigarette smoke extract induces epithelial–mesenchymal transition of human bladder cancer T24 cells through activation of ERK1/2 pathway	E-cadherin,ZO-1,Vimentin,N-cadherin,Snail, p38 or JNK,	ERK1/2 pathway
Ming sun et al.	2017	28860802	0	Fibrous sheath interacting protein 1 overexpression is associated with unfavorable prognosis in bladder cancer: a potential therapeutic target	FSIP1,	
Marcus stapf et	2017	28435259	0	Methotrexate-coupled nanoparticles and magnetic nanochemothermia for the relapse-free treatment of T24 bladder tumors	p-ERK1/2, BCL2, and HSP70	p-ERK1/2
Yarong Song et	2017	28978086	0	AMPK activation-dependent autophagy compromises oleanolic acid-induced cytotoxicity in human bladder cancer cells	Bcl-2,Bax,Caspase-3,AMPKα, P-AMPKα (Thr 172), mTOR, P-mTOR (Ser 2448), ULK-1, P-ULK1 (Ser 317),LC3B,	AMPK-mTOR-ULK1
LINGLING SI ε	2017	28693160	0	Isoliquiritigenin induces apoptosis of human bladder cancer T24 cells via a cyclin-dependent kinase-independent mechanism	Bax, Bim, Apaf-1, caspase-9 ,caspase-3, Bcl-2, CDK2,	CDK2
Terumichi Shin	2017	28013036	0	The Involvement of Hepatocyte Growth Factor-MET-Matrix Metalloproteinase 1 Signaling in Bladder Cancer Invasiveness and Proliferation. Effect of the MET Inhibitor, Cabozantinib (XL184), on Bladder Cancer Cells	MET,XL184,MMP1,	HGF-MET ,HGF-MET-MMP1
YOSHINORI SI	2017	28693265	0	Overexpression of the transmembrane protein BST-2 induces Akt and Erk phosphorylation in bladder cancer	BST-2	BST-2 -Akt and Erk
Jing Shi et al.	2017	28705118	0	Curcumin inhibits bladder cancer progression via regulation of β-catenin expression	β-catenin，Vimentin,N-cadherin，E-cadherin	Wnt/β-catenin
Jie Shi et al.	2017	27622605	0	Lentivirus-mediated p21/Waf-1 short hairpin RNA enhances the cytotoxic effects and replicative potential of a bladder cancer-specific oncolytic adenovirus in vitro	Fas, caspase-3, cleaved caspase-3, Bax, and Bcl-2,p21/Waf-1	p21/Waf-1
MO SHEN et al	2017	28586003	0	Lymphotoxin β receptor activation promotes mRNA expression of RelA and pro-inflammatory cytokines TNF α and IL-1 β in bladder cancer cells	LT β R,LT α ,LT β ,LIGHT,(TNF) α,IL-6,(IL)-1 β,CyclinD1,,Survivin	LT β R-(NF)- κ B
Angela Patricia	2017	28870684	0	Isoliquiritigenin pretreatment attenuates cisplatin induced proximal tubular cells (LLC-PK1) death and enhances the toxicity induced by this drug in bladder cancer T24 cell line	IsoLQ,Cisp,cleaved caspase-3，HQ-1,NF k B,	,NF k B,
Andrew M. Hau	2017	27777073	1	mTORC2 activation is regulated by the urokinase receptor (uPAR) in bladder cancer	mTORC2,uPAR,PAI-1,	mTORC2 -Akt S473

Table S1. The list of bladder cancer related articles either used or not used RT4.

Author	Year	PMID	using RT4 or not(0 represents No;1 represents Yes)	Title	Gene	Pathway
Choo-Aun Neol	2017	29280977	1	Flaccidoxide-13-Acetate Extracted from the Soft Coral Cladiella kashmani Reduces Human Bladder Cancer Cell Migration and Invasion through Reducing Activation of the FAK/PI3K/AKT/mTOR Signaling Pathway	FAK/PI3K/AKT/mTOR,f RhoA, Ras, GRB2, MKK7 and MEKK3,MMP-2 and MMP-9, (TIMP-1) and TIMP-2	FAK/PI3K/AKT/mTOR
Makito Miyake	2017	28415608	0	Collagen type IV alpha 1 (COL4A1) and collagen type XIII alpha 1 (COL13A1) produced in cancer cells promote tumor budding at the invasion front in human urothelial carcinoma of the bladder	COL4A1 and COL13A1,N-cadherin，E-cadherin,Vimentin,MAPK,AKT,Dynamin	TGF-β and Wnt/β-catenin
Mohammad Re:	2017	28235715	0	Down-regulation of anti-apoptotic genes in tumor cell lines is facilitated by suppression of OCT4B1	OCT4B1, CFLAR,CASP2, IGF1R andTNF	BNIPs-OCT4B1
Li Mengdan et a	2017	28444938	0	Low concentration arsenite activated JAK2/STAT3 signal and increased proliferative factor expressions in SV-HUC-1cells after short and long time treatment	JAK2，STAT3，cyclin D1, COX-2，PCNA, BCL，MMP1，CCND	JAK2/STAT3
Xia-Wa Mao et	2017	29100384	0	CUL4B promotes bladder cancer metastasis and induces epithelial-to-mesenchymal transition by activating the Wnt/β-catenin signaling pathway	Wnt/β-catenin，CUL4B，N-cadherin，E-cadherin,Vimentin,cyclin D1,,MMP7,WIF-1,ZEB1, ZEB2,,	Wnt/β-catenin
Eglal Mahgoub	2017	28738532	0	Genipin attenuates cisplatin-induced nephrotoxicity by counteracting oxidative stress, inflammation, and apoptosis	WST-1,NF- k B,MAPK,(JNK,	NF- k B
Yingyu Ma et a	2017	28947955	0	1,25D 3 differentially suppresses bladder cancer cell migration and invasion through the induction of miR-101-3p	miR-101-3p,VDR,E-cadherin, MMP-2 ,MMP-9 ,LncRNA SPRY4-IT1,EZH2	miR-101-3p-EZH2
Ke-Wang Luo	2017	28040581	0	EGCG inhibited bladder cancer SW780 cell proliferation and migration both in vitro and in vivo via down-regulation of NF-κB and MMP-9	NF-κB and MMP-9，caspases-8,-9and-3,Bax,Bcl-2， PARP	NF-κB
Jinjin Lu et al.	2017	28107184	1	Tunneling nanotubes promote intercellular mitochondria transfer followed by increased invasiveness in bladder cancer cells	Akt/mTOR，4EBP1， p70S6K，	Akt/mTOR
ZHI-HUA LIU	2017	28599483	0	Effects of programmed death-ligand 1 expression on OK-432 immunotherapy following transurethral resection in non-muscle invasive bladder cancer	PD-L1，IL-2，INF- α ,，INF- γ，	JAK/STATl/IRF-1 and MEK/ERK/STAT1
Zhongyuan Liu	2017	28969069	0	LncRNA plasmacytoma variant translocation 1 is an oncogene in bladder urothelial carcinoma	PVT1， MDR1 and MRP1，β-catenin and CyclinD1	Wnt/β-catenin
ZHIqI LIU et a	2017	28259934	0	Curcumin reverses benzidine-induced epithelial-mesenchymal transition via suppression of ERK5/AP-1 in SV-40 immortalized human urothelial cells	MEK/ERK5，JNK1/2/3，p38 α / β / γ / δ，BMK1， ZO-1，N-cadherin，E-cadherin,Vimentin， MMP-2 andMMP-9，	ERK5/AP-1
Qian liu et al.	2017	28503069	0	Downregulation of long noncoding rna TUg1 inhibits proliferation and induces apoptosis through the TUg1/mir-142/ZeB2 axis in bladdercancer cells	TUg1,ZEB2，miR-142，β-catenin，c-Myc	TUg1/mir-142/ZeB2
Jia Liu et al.	2017	29088864	0	Rab27A overexpression promotes bladder cancer proliferation and chemoresistance through regulation of NF-κB signaling	Rab27A，cyclin D1, cyclin E, p-IκB, p-p65, Bcl-2, cIAP1, cIAP2	NF-κB
Chun-Ping Liu	2017	29155869	0	NF-κB pathways are involved in M1 polarization of RAW 264.7 macrophage by polyporus polysaccharide in the tumor microenvironment	(IL)-6，INOS，(TNF)-α，CD40, CD284, P65-NF-κB, IKB,INOS and COX-2	NF-κB,

Table S1. The list of bladder cancer related articles either used or not used RT4.

Author	Year	PMID	using RT4 or not(0 represents No;1 represents Yes)	Title	Gene	Pathway
Yi-Chia Lin et a	2017	29074848	0	Acridine orange exhibits photodamage in human bladder cancer cells under blue light exposure	LC3，caspase-3/7，	
Ji-Fan lin et al.	2017	28553083	0	cisplatin induces protective autophagy through activation of Becn1 in human bladder cancer cells	Becn1，LC3, p62,c-Casp3), PARP, Casp9, c-Casp8, ATG7, ATG12,	NFκB
Junhao Lin et al	2017	28445936	0	Increased expression of ZEB1-AS1 correlates with higher histopathological grade and promotes tumorigenesis in bladder cancer	ZEB1-AS1	
Xuan Liang et a	2017	28081738	0	Silencing of Armadillo Repeat-Containing Protein 8 (ARMc8) Inhibits TGF-β-Induced EMT in Bladder Carcinoma UMUC3 Cells	ARMc8，TGF-b1，N-cadherin，E-cadherin,Vimentin，cyclin D1,β-catenin，c-Myc	Wnt/b-catenin
Yuchao Li et al.	2017	28382144	0	B7-H3 Promotes the Migration and Invasion of Human Bladder Cancer Cells via the PI3K/Akt/STAT3 Signaling Pathway	B7-H3,PI3K/Akt/STAT3,MMP-9,CTLA-4,PD-1	PI3K/Akt/STAT3
Xueqiang Li et a	2017	28521156	0	2-Oxo-3, 4-dihydropyrimido[4, 5-d]pyrimidinyl derivatives as new irreversible pan fibroblast growth factor receptor (FGFR) inhibitors	FGFR，ERK1/2，pERK，	ERK1/2
Tian Li et al.	2017	28641488	0	UCA1 involved in the metforminregulated bladder cancer cell proliferation and glycolysis	UCA1，p-mTOR/mTOR, p-STAT3/STAT3, and p-HK2/HK2，	mTOR–STAT3
SHUN LI et al.	2017	28588714	0	Oxymatrine inhibits proliferation of human bladder cancer T24 cells by inducing apoptosis and cell cycle arrest	p53，Bcl-2，Bax，caspase-3	p53-Bax
SHUWEN LI et	2017	28781650	0	Synergistic effect of bladder cancer-specific oncolytic adenovirus in combination with chemotherapy	E1A，	Ad5-UPII-E1A
Jiangfeng Li et a	2017	28837140	0	MET/SMAD3/SNAIL circuit mediated by miR-323a-3p is involved in regulating epithelial–mesenchymal transition progression in bladder cancer	MET/SMAD3/SNAIL，miR-323a-3p，N-cadherin，E-cadherin,Vimentin，	AKT/GSK-3β/SNAIL
Hui-Jin Li et al.	2017	29130995	0	LncRNA UCA1 Promotes Mitochondrial Function of Bladder Cancer via the MiR-195/ARL2 Signaling Pathway	UCA1,MiR-195,ARL2,GLUD1,SDHA,HSP60,PDH,PHB1,VDAC,SOD1,	MiR-195/ARL2
Chuanchang Li	2017	28631573	0	Effects of miR-1236-3p and miR-370-5p on activation of p21 in various tumors and its inhibition on the growth of lung cancer cells	miR-1236-3p and miR-370-5p,cyclin D1-CDK4/CDK6,	cyclin D1-CDK4/CDK6
Bo LI et al.	2017	29270748	0	Overexpression of CircRNA BCRC4 Regulates Cell Apoptosis and MicroRNA-101/EZH2 Signaling in Bladder Cancer	CircRNA BCRC4,MicroRNA-101/EZH2	MicroRNA-101/EZH2
BO KOU et al.	2017	29048631	0	Autophagy induction enhances tetrandrine-induced apoptosis via the AMPK/mTOR pathway in human bladder cancer cells	AMPK/mTOR , LC3-II ,LC3-Ⅰ,p-AMPK (Thr172), AMPK, p-ACC (Ser79), ACC,p-mTOR (Ser2448), mTOR, p70S6K (Thr389), p70S6K (Ser371), p-4EBP1	AMPK/mTOR
WON TAE KIM	2017	28498422	0	Garlic extract in bladder cancer prevention: Evidence from T24 bladder cancer cell xenograft model, tissue microarray, and gene network analysis	AKAP12 and RDX,RAB13,RAB13, PLA2G2D, OPA3, POLR1B, SSTR2, SPG7,RBM14, BMP8B, RAD51, and CDK10	

Table S1. The list of bladder cancer related articles either used or not used RT4.

Author	Year	PMID	using RT4 or not(0 represents No;1 represents Yes)	Title	Gene	Pathway
Kunbin Ke et al	2017	27664399	0	In silico prediction and in vitro and in vivo validation of acaricide fluazuron as a potential inhibitor of FGFR3 and a candidate anticancer drug for bladder carcinoma	FGFR3，FRS2- α,AKT, and ERK	FGFR3-AKT,
Minyong Kang	2017	28165387	0	Concurrent Autophagy Inhibition Overcomes the Resistance of Epidermal Growth Factor Receptor Tyrosine Kinase Inhibitors in HumanBladder Cancer Cells	ATG12，BFA1, CQ or 3-MA，ATG12，	LC3-GFP
KYUNG-HWA	2017	28693242	0	Bufalin sensitizes human bladder carcinoma cells to TRAIL-mediated apoptosis	TRAIL,DR4 and DR5 Bax, BCL2,XIAP,cFLIP,DR,PARP	TRAIL-DR
Koji KAMeyAM	2017	27909718	0	Enzalutamide inhibits proliferation of gemcitabine-resistant bladder cancer cells with increased androgen receptor expression	AR,CyclinB1、CyclinD1,	AR-CyclinD1/CyclinB1
Andre R. Jordar	2017	27419371	0	Antitumor activity of sulfated hyaluronic acid fragments in prelinical models of bladder cancer	HYAL-1,Caspase-3, Caspase-8, Caspase-9,PARP,DR4,PI-3K , AKT	PI-3K-AKT
Subin Jin et al.	2017	28665070	0	Identification of Downstream Genes of the mTOR Pathway that Predict Recurrence and Progression in Non-Muscle Invasive High-Grade Urothelial Carcinoma of the Bladder	FABP4, H19, ANXA10, and UPK3A,FOXD3, ATP7A, plexin D1, and ADAMTS5,	mTOR
Guosong Jiang	2017	28223425	0	Role of STAT3 and FOXO1 in the Divergent Therapeutic Responses of Non-metastatic and Metastatic Bladder Cancer Cells to miR-145	STAT3 and FOXO1， miR-145，， STAT1 and p-STAT1， P27， JAK2, p-JAK2，	miR-145-FOXO1
Mitsuho Imai-Sı	2017	29190895	0	Silibinin suppresses bladder cancer through down-regulation of actin cytoskeleton and PI3K/Akt signaling pathways	HOTAIR，ZFAS1，H3K4，H3K4me3，AcH3，SOS1,AKT,EGFR,RAS,DDR1,PAK1	PI3K/Akt
Eu Chang Hwar	2017	28266813	0	Generation of potent cytotoxic T lymphocytes against in male patients with non-muscle invasive bladder cancer by dendritic cells loaded with dying T24 bladder cancer cells	Ag85A,	
Ying Huang et	2017	28977858	0	Identification of hMex-3A and its effect on human bladder cancer cell proliferation	hMex-3A，IGF1，	hMex-3A-IGF1，
Xiao-Long Hua	2017	28430744	0	Activation of a c-Jun N-terminal kinase-mediated autophagy pathway attenuates the anticancer activity of gemcitabine in human bladder cancer cells	Caspase-3,PARP，LC3 and p62，JNK1， c-Jun，	JNK1-PARP
Xinbo Huang et	2017	28702647	0	An enhanced hTERT promoter-driven CRISPR/Cas9 system selectively inhibits the progression of bladder cancer cells	hTERT， GAL4-P65，5XUAS，Cas9， HRAS，	hTERT-GAL4-P65
Sung-Ying Hua	2017	28757590	0	Tanshinone IIA Inhibits Epithelial-Mesenchymal Transition in Bladder Cancer Cells via Modulation of STAT3-CCL2 Signaling	Tan-IIA， MMP-9/-2，E-cadherin，STAT3-CCL2，N-cadherin，Vimentin，SLUG,SNAIL	STAT3-CCL2
Chao Huang et a	2017	28057023	0	XIAP BIR domain suppresses miR-200a expression and subsequently promotes EGFR protein translation and anchorage-independent growth of bladder cancer cell	XIAP,EGFR,	XIAP-miR-200a-c-Jun
Viktorija Herceg	2017	28472723	0	Activity of phosphatase-sensitive 5-aminolevulinic acid prodrugs in cancer cell lines	PpIX	
Andrew M. Hau	2017	28427860	0	Differential mTOR pathway profiles in bladder cancer cell line subtypes topredict sensitivity to mTOR inhibition	mTOR，AKT1, AKT2, AKT3, EIF4EBP1,MTOR, RICTOR, RPS6, and RPTOR，	mTOR

Table S1. The list of bladder cancer related articles either used or not used RT4.

Author	Year	PMID	using RT4 or not(0 represents No;1 represents Yes)	Title	Gene	Pathway
Shuko Hata et a	2017	28947209	0	Expression of AR, 5αR1 and 5αR2 in bladder urothelial carcinoma andrelationship to clinicopathological factors	AR, 5αR1 and 5αR2，	AR
LIN HAO et al.	2017	28521465	0	Tumor necrosis factor-related apoptosis-inducing ligand inhibits proliferation and induces apoptosis of prostate and bladder cancer cells	TRAIL	
Min Ho Han et	2017	27654302	0	Fucoidan Induces ROS-Dependent Apoptosis in 5637 Human Bladder Cancer Cells by Downregulating Telomerase Activity via Inactivation of the PI3K/Akt Signaling Pathway	Bax/Bcl-2，hTERT, c-myc, and Sp1,PARP,	PI3K/Akt
W. GU et al.	2017	28592119	0	Tea Polyphenol inhibits autophagy to sensitize Epirubicin-induced apoptosis in human bladder cancer cells	LC3,JNK,BCL-2,PARP,Caspase-3,	JNK/Bcl-2/Beclin-1
HAO GENG et :	2017	28123584	0	Cigarette smoke extract-induced proliferation of normal human urothelial cells via the MAPK/AP-1 pathway	MAPK/AP-1,CyclinD1,PCNA,P21,JNK,P38,ERK, JUN	MAPK/AP-1
SHENg-LIN gA	2017	28791418	0	TRPM7 is overexpressed in bladder cancer and promotes proliferation, migration, invasion and tumor growth	TRPM7	
Ahn, K. I., et al.	2017	29070760	1	Induction of apoptosis by ethanol extract of Citrus unshiu Markovich peel in human bladder cancer T24 cells through ROS-mediated inactivation of the PI3K/Akt pathway	caspase-8，-9和-3 Bcl-2	ROS介导的PI3K / Akt
Ai, X. L., et al.	2017	28074341	0	Gap junction protein connexin43 deregulation contributes to bladder carcinogenesis via targeting MAPK pathway.	Cx43	MAPK JNK和ERK
Bacchetti, T., et	2017	28430636	0	Exploring the role of paraoxonase-2 in bladder cancer: analyses performed on tissue samples, urines and cell cultures	PON2	
Bettiga, A., et al	2017	28191815	1	Bladder cancer cell growth and motility implicate cannabinoid 2 receptor-mediated modifications of sphingolipids metabolism	CB	Akt
Bi, H., et al	2017	28968986	1	Effect of wound fluid on chemotherapy sensitivity of T24 bladder cancer cells with different enhancer of zeste homolog 2 status	EZH2 WF	
Bognar, Z., et a	2017	29220397	0	Desethylamiodarone-A metabolite of amiodarone-Induces apoptosis on T24 human bladder cancer cells via multiple pathways.	DEA PARP-1和caspase-3 BMI1	Bax / Bcl-2
Cao, H. L., et al	2017	28714368	0	Cordycepin induces apoptosis in human bladder cancer cells via activation of A3 adenosine receptors	caspase-3	A3 Adenosine receptors and AKT pathway
Cao, J. Y., et al	2017	28746469	0	Interleukin-27 augments the inhibitory effects of sorafenib on bladder cancer cells	MMP）-2和MMP-9	AKT / mTOR / MAPK
Cao, W., et al.	2017	28789701	0	A novel bladder cancer - specific oncolytic adenovirus by CD46 and its effect combined with cisplatin against cancer cells of CAR negative expression	CRAds p53，Bax和caspase-3 Bcl-2	
Carmo, A., et al	2017	28618950	0	MiR-1-3p inhibits the proliferation and invasion of bladder cancer cells by suppressing CCL2 expression	miR-1-3p和CCL2	
Cesario, J. M., e	2017	27752923	0	A simple method to induce hypoxia-induced vascular endothelial growth factor-A (VEGF-A) expression in T24 human bladder cancer cells	VEGF-A HIF-1α	

Table S1. The list of bladder cancer related articles either used or not used RT4.

Author	Year	PMID	using RT4 or not(0 represents No;1 represents Yes)	Title	Gene	Pathway
Chang, H. Y., e	2017	28841878	1	The role of Lutheran/basal cell adhesion molecule in human bladder carcinogenesis.	Lu / BCAM F-actin	Erk phosphorylation
Chen, M., et al.	2017	28534961	0	Disruption of serine/threonine protein phosphatase 5 inhibits tumorigenesis of urinary bladder cancer cells.	PPP5C c-myc和CDK4 p27，BAD和 Beclin1	
Chen, M. C., et	2017	28710377	0	Antiangiogenic activity of phthalides-enriched Angelica Sinensis extract by suppressing WSB-1/pVHL/HIF-1alpha/VEGF signaling in bladder cancer	HIF-1α	WSB-1 / VHL / HIF-1α/ VEGF PI3K / AKT / mTOR
Cui, X., et al.	2017	28139689	1	NF-kappaB suppresses apoptosis and promotes bladder cancer cell proliferation by upregulating survivin expression in vitro and in vivo	Survivin	NF-κB/ survivin
DT, D. E. O., et	2017	28229968	1	Cytotoxic and toxicogenomic effects of silibinin in bladder cancer cells with different TP53 status	FRAP / mTOR，FGFR3，AKT2和 DNMT1基因以及miR100和miR203	TP53
Du, P., et al.	2017	28739745	0	Reduced Expression of Metastasis Suppressor-1 (MTSS1) Accelerates Progression of Human Bladder Uroepithelium Cell Carcinoma.	MTSS1	
Dyshlovoy, S. A	2017	28143426	1	The marine triterpene glycoside frondoside A induces p53-independent apoptosis and inhibits autophagy in urothelial carcinoma cells.	caspase-3，-8和-9，PARP，Bax，p21	
Elbarbary, R. A	2017	28827400	0	UPF1 helicase promotes TSN-mediated miRNA decay.	UPF1	
Fan, B., et al.	2017	28968601	0	Fangchinoline Induces Apoptosis, Autophagy and Energetic Impairment in Bladder Cancer.	LC3-II / LC3-I p62	
Fan, Z., et al	2017	27433879	0	Effect of autophagy on cisplatin-induced bladder cancer cell apoptosis.	GFP-LC3	mTOR / P70S6K
Feng, C., et al.	2017	29042941	0	Epigallocatechin gallate inhibits the growth and promotes the apoptosis of bladder cancer cells.	TFPI-2	
Gan, Y., et al.	2017	29163683	0	Knockdown of HMGN5 increases the chemosensitivity of human urothelial bladder cancer cells to cisplatin by targeting PI3K/Akt signaling.	HMGN5 slug，E-cadherin和VEGF-C	PI3K）/ Akt
Gao, F., et al.	2017	28521777	0	CIP2A mediates fibronectin-induced bladder cancer cell proliferation by stabilizing beta-catenin.	FN和CIP2A	
Chen, D., et al.	2017	28283461	0	Induction of mitochondrial-dependent apoptosis in T24 cells by a selenium (Se)-containing polysaccharide from Ginkgo biloba L. leaves	caspase-9，caspase-3和PARP	
Chen DJ1, Chen	2016	27660415	0	Downregulation of DOCK1 sensitizes bladder cancer cells to cisplatin through preventing epithelial-mesenchymal transition	DOCK1、E-Cad、vimentin	EMT
Chen J1, Wang	2016	26733306	0	Maspin enhances cisplatin chemosensitivity in bladder cancer T24 and 5637 cells and correlates with prognosis of muscle-invasive bladder cancer patients receiving cisplatin based neoadjuvant chemotherapy	Maspin,Caspase3,Bcl-2,	PI3K/ AKT/mTOR
Chen M1, Zhua	2016	27018306	0	Tetracycline-inducible shRNA targeting antisense long non-coding RNA HIF1A-AS2 represses the malignant phenotypes of bladder cancer	HIF1A-AS2,	

Table S1. The list of bladder cancer related articles either used or not used RT4.

Author	Year	PMID	using RT4 or not(0 represents No;1 represents Yes)	Title	Gene	Pathway
Chiu KY1, Wu (	2016	26679052	0	Inhibition of growth, migration and invasion of human bladder cancer cells by antrocin, a sesquiterpene lactone isolated from Antrodia cinnamomea, and its molecular mechanisms	Fas，DR5，Bax,caspase-3, -8 and -9	FAK-paxillin,ERK-c-Fos-MMP2
Choi BH1, Lee I	2016	27706018	0	Controls of Nuclear Factor-Kappa B Signaling Activity by 5'-AMP-Activated Protein Kinase Activation With Examples in Human Bladder Cancer Cells	NF-κB	TLR4 / NF-κB
Choi EO1, Park	2016	27571890	0	Baicalein induces apoptosis via ROS-dependent activation of caspases in human bladder cancer 5637 cells	PARP,caspase-3, -8 and -9 ,Bcl-2 ,MMP	caspase
Péchery A1, Fau	2016	27638828	1	Apoptotic effect of the selective PPARbeta/delta agonist GW501516 in invasive bladder cancer cells	Bcl-2,Bax,Bid,p53,p21,细胞色素c	caspase-
Chuang CH, Du	2016	26777732	0	Immunosensor for the ultrasensitive and quantitative detection of bladder cancer in point of care testing	Galectin-1	
Chung, Y. H.	2016	27349281	0	RIP kinase-mediated ROS production triggers XAF1 expression through activation of TAp73 in casticin-treated bladder cancer cells	XAF1,TAp73,PARP ,Mcl-2 ,Bcl-2,TAp63,p53	JNK/p38-MAPK,
Coccia A1, Mos	2016	27748855	0	Extra-virgin olive oil phenols block cell cycle progression and modulate chemotherapeutictoxicity in bladder cancer cells.	PARP,caspase-3,-9	
Deng QF1, Sun	2016	26883573	0	Cigarette smoke extract induces the proliferation of normal human urothelial cells through the NF-kappaB pathway	NF-κB p65, p50, IκBα, p-IKKα/β, cyclin D1, p21 and PCNA	NF-kappaB
Ding G	2016	27158349	0	Androgen receptor (AR) promotes male bladder cancer cell proliferation and migration via regulating CD24 and VEGF	CD24,VEGF，MMP9	
Egawa H	2016	26837847	0	The miR-130 family promotes cell migration and invasion in bladder cancer through FAK and Akt phosphorylation by regulating PTEN	PTEN	FAK and Akt phosphorylation
Fu X,	2016	26209051	0	XIAP inhibitor Embelin inhibits bladder cancer survival and invasion in vitro	XIAP,PI3K，AKt，p-AKt	
Gabig TG1,	2016	27520378	1	Clostridium perfringens enterotoxin as a potential drug for intravesical treatment of bladder cancer	CLDN4	
Gawlik-Rzemiei	2016	27478472	0	Silencing expression of the NANOG gene and changes in migration and metastasis of urinary bladder cancer cells	MMP-2; MMP-9; RNAi; TIMP-1;	
Ge, Q.	2016	27055644	0	[Effect of synthetic small double-stranded RNA on the development of bladder cancer by activating P21 expression]	p21,CDK4,CDK6	
Gong YQ	2016	28101210	0	UBE2T silencing suppresses proliferation and induces cell cycle arrest and apoptosis in bladder cancer cells	UBE2T ,	
Greco KA	2016	26876462	0	PLK-1 Silencing in Bladder Cancer by siRNA Delivered With Exosomes	PLK-1	
Gross-Cohen, M	2016	26968815	0	Heparanase 2 expression inversely correlates with bladder carcinoma grade and stage	Hpa2	
He, A.	2016	27514530	0	Over-expression of long noncoding RNA BANCR inhibits malignant phenotypes of human bladder cancer	Caspase-3 ,BANCR	
Hinrichsen, S.	2016	26780055	0	Cytotoxic activity of selenosulfate versus selenite in tumor cells depends on cell line and presence of amino acids	HepG2	

Table S1. The list of bladder cancer related articles either used or not used RT4.

Author	Year	PMID	using RT4 or not(0 represents No;1 represents Yes)	Title	Gene	Pathway
KE YAO HU	2016	27373212	0	Targeting of MCT1 and PFKFB3 influences cell proliferation and apoptosis in bladder cancer by altering the tumor microenvironment	PFKFB3，MCT1，CD31,CD305	
Huang, K	2016	27852389	0	[Expression of PAK1 in bladder cancer and its influence on invasion of bladder cancer cells]	PAK1	
Iskender, B	2016	27456363	0	Reprogramming bladder cancer cells for studying cancer initiation and progression	OCT4, SOX2, KLF4 and c-MYC	
Iskender, B.	2016	26718217	0	Inhibition of epithelial-mesenchymal transition in bladder cancer cells via modulation of mTOR signalling	ERK 1/2，N-cadherin, Snail, Slug, beta-catenin	mTOR
Jiang, YJ (	2016	26715272	0	Rab23 is overexpressed in human bladder cancer and promotes cancer cell proliferation and invasion	Rab23，	NF-kappa B
Jou, Y. C.	2016	27557492	0	Foxp3 enhances HIF-1alpha target gene expression in human bladder cancer through decreasing its ubiquitin-proteasomal degradation	Foxp3 ， GLUT-4,-9, and VEGF-A, B-, D ， HIF-1α	
Karkoulis, P. K.	2016	26662567	0	17-DMAG induces heat shock protein 90 functional impairment in human bladder cancer cells: knocking down the hallmark traits of malignancy	Cdk4, E2F1 and Cyclin D1，PARP and Lamin A/C，(caspase-8, caspase-9 and caspase-3，Hsp90, co-chaperoneHsp70, and CHIP	HGF/c-Met
Kashiwagi, E.	2016	27322140	0	Androgen receptor activity modulates responses to cisplatin treatment in bladder cancer	AR ， NF-κB ，	NF-κB
Zhu Y1, Dai B1	2016	27130667	0	Long non-coding RNA LOC572558 inhibits bladder cancer cell proliferation and tumor growth by regulating the AKT-MDM2-p53 signaling axis	Akt, CREB, BAD,FAK, Hsp90, P53, mTOR, Myc,MDM2	**AKT-MDM2-p53 signaling**
Zhou J1, Duan	2016	27698912	0	MiR-193a-5p Targets the Coding Region of AP-2alpha mRNA and Induces Cisplatin Resistance in Bladder Cancers	AP-2α, p21, MMP9	
Zhong J1,2, Ch	2016	26695143	1	Testis expressed 19 is a novel cancer-testis antigen expressed in bladder cancer	TEX19	
Zheng SS1, Ga	2016	26718084	0	Downregulation of fatty acid synthase complex suppresses cell migration by targeting phosphor-AKT in bladder cancer	AKT, FASN, MMP-9	PI3K/AKT pathway and MMP-9
Zheng L1, Che	2016	26648574	0	Capsaicin enhances anti-proliferation efficacy of pirarubicin via activating TRPV1 and inhibiting PCNA nuclear translocation in 5637 cells	TRPV1, PCNA	
Zhang Y1, Nol	2016	27771248	0	Dynamin2 GTPase contributes to invadopodia formation in invasive bladder cancer cells	Arp2/3	
Zhang Y1, Liu	2016	26983576	0	Tetrandrine reverses epithelial-mesenchymal transition in bladder cancer by downregulating Gli-1	Gli-1	Hedgehog signaling pathway
Zhang S1, Pei	2016	26615421	1	Functional characterization of the tumor suppressor CMTM8 and its association with prognosis in bladder cancer	CMTM8	
Zhang R1, Che	2016	27556506	0	Cheliensisin A (Chel A) induces apoptosis in human bladder cancer cells by promoting PHLPP2 protein degradation	PHLPP2	JNK/C-Jun phosphorylation and activation

Table S1. The list of bladder cancer related articles either used or not used RT4.

Author	Year	PMID	using RT4 or not(0 represents No;1 represents Yes)	Title	Gene	Pathway
Zhang P1, Wan;	2016	26889242	0	Suppression of SIPA-1 expression may reduce bladder cancer invasion and metastasis via the downregulation of E-cadherin and ZO-1	SIPA-1	
Zhang N1, Bi X	2016	27350089	0	TGF-beta1 promotes the migration and invasion of bladder carcinoma cells by increasing fascin1 expression	fascin1	
Zhang M1, Zhu	2016	27133066	0	MiR-194 inhibits cell proliferation and invasion via repression of RAP2B in bladder cancer	RAP2B	miR-194/RAP2B axis
Zhang J1, Wang	2016	26723509	0	MicroRNA-542-3p suppresses cellular proliferation of bladder cancer cells through post-transcriptionally regulating survivin	survivin	miR-542-3p-survivin signal axis
Zhang H1,2, Pr	2016	27189164	1	Biased Expression of the FOXP3Delta3 Isoform in Aggressive Bladder Cancer Mediates Differentiation and Cisplatin Chemotherapy Resistance	FOXP3Δ3	
Zhang C1, Ma Σ	2016	26775686	0	MicroRNA-30a as a prognostic factor in urothelial carcinoma of bladder inhibits cellular malignancy by antagonising Notch1	Notch1	
Yang X1, Jiang	2016	27221781	0	Licochalcone A induces T24 bladder cancer cell apoptosis by increasing intracellular calcium levels	Bcl, Apaf-1, calpain 2, caspase	
Yang J1, Yuan	2016	26494000	1	miR-186 downregulates protein phosphatase PPM1B in bladder cancer and mediates G1-S phase transition	PPM1B	NF-κB signaling pathways
Xie X1, Pan J1,	2016	26820254	0	Gene expression profiling of microRNAs associated with UCA1 in bladder cancer cells	UCA1, p27kip1	PI3K-Akt signaling pathway
Wu Z1,2, Wang	2016	27977784	0	High Expression of Derlin-1 Is Associated with the Malignancy of Bladder Cancer in a Chinese Han Population	Derlin-1	
Wu D1, Niu X2	2016	27035227	0	MicroRNA-335 is downregulated in bladder cancer and inhibits cell growth, migration and invasion via targeting ROCK1	ROCK1	miR-335/ROCK1 axis
Wu CL1, Ho JY	2016	27058893	0	MiR-429 reverses epithelial-mesenchymal transition by restoring E-cadherin expression in bladder cancer	ZEB1	
Wang X1, Wu (	2016	27356628	0	MicroRNA335 inhibits bladder cancer cell growth and migration by targeting mitogenactivated protein kinase 1	MAPK1	
Wang X1, Chen	2016	26873485	0	The SMAD2/3 pathway is involved in hepaCAM-induced apoptosis by inhibiting the nuclear translocation of SMAD2/3 in bladder cancer cells	SMAD/PARP	SMAD2/3 pathway
Wang SC1, Hua	2016	27404495	1	Gene Expression and DNA Methylation Status of Glutathione S-Transferase Mu1 and Mu5 in Urothelial Carcinoma	GSTM1/GSTM5	
Wang S1,2, Lei	2016	27391608	0	The Reversal Effect and Its Mechanisms of Tetramethylpyrazine on Multidrug Resistance in Human Bladder Cancer	MRP1, GST, BCL-2, LRP, TOPO-II	
Wang J1, Zhao	2016	27571748	0	miR-451 suppresses bladder cancer cell migration and invasion via directly targeting c-Myc	c-Myc	
Wang H1, Ke C	2016	27830467	0	MicroRNA-92 promotes invasion and chemoresistance by targeting GSK3beta and activating Wnt signaling in bladder cancer cells	GSK3β	Wnt/c-myc/MMP7 signaling
Wang DG1, Zha	2016	27687590	0	Fiber-modified adenovirus-mediated suicide gene therapy can efficiently eliminate bladder cancer cells in vitro and in vivo	HSV-TK	

Table S1. The list of bladder cancer related articles either used or not used RT4.

Author	Year	PMID	using RT4 or not(0 represents No;1 represents Yes)	Title	Gene	Pathway
Wang C1,2, Ge	2016	27012825	0	Targeted p53 activation by saRNA suppresses human bladder cancer cells growth and metastasis	p53, Cyclin-CDK genes (Cyclin D1 and CDK4/6) , EMT-associated genes (E-cadherin, β-catenin, ZEB1 and Vimentin)	
Tsui KH1, Lin `	2016	26965996	1	Prostate-derived ets factor represses tumorigenesis and modulates epithelial-to-mesenchymal transition in bladder carcinoma cells	PDEF	
Tian DW1, Hu l	2016	27356780	0	Biological effects of eukaryotic recombinant plasmid pReceiver-M61-BAI-1 transfection on T24 cells and HUVECs	BAI-1	
Tan ST1, Liu S`	2016	26987391	0	TRIM29 Overexpression Promotes Proliferation and Survival of Bladder Cancer Cells through NF-kappaB Signaling	TRIM29, cyclin, Bcl	PKC and NF-κB signaling pathways
Sun Y1, Guan Z	2016	26647959	0	NF-kappaB signaling plays irreplaceable roles in cisplatin-induced bladder cancer chemoresistance and tumor progression	ABCB1	NF-κB signaling
Sun Y1, Guan Z	2016	26717965	0	HIF-1alpha/MDR1 pathway confers chemoresistance to cisplatin in bladder cancer	MDR1, HIF-1α	HIF-1α/MDR1 pathway
Sun X1, Deng (	2016	26776764	0	Curcumin reverses benzidine-induced cell proliferation by suppressing ERK1/2 pathway in human bladder cancer T24 cells	cyclin D1, PCNA, p21	ERK1/2 pathway
Sun E1, Zhang	2016	26224479	0	Down-regulation of Sphk2 suppresses bladder cancer progression	SphK2	
Shen KH1,2,3,	2016	27440446	0	Role of galectin-1 in urinary bladder urothelial carcinoma cell invasion through the JNK pathway	galectin-1, LGALS1	Ras-Rac1-MEKK4-JNK-AP1 signaling pathway
Scarpa ES1, Em	2016	27812566	0	Betacyanins enhance vitexin-2-O-xyloside mediated inhibition of proliferation of T24 bladder cancer cells	BCL2, BAX, BIRC5, CTNNB1	
Sahu D1, Gupta	2016	27979310	1	Argininosuccinate Synthetase 1 Loss in Invasive Bladder Cancer Regulates Survival through General Control Nonderepressible 2 Kinase-Mediated Eukaryotic Initiation Factor 2alpha Activity and Is Targetable by Pegylated Arginine Deiminase	ADI-PEG 20	
Qian K1,2, Wan	2016	27775662	0	Capsaicin Suppresses Cell Proliferation, Induces Cell Cycle Arrest and ROS Production in Bladder Cancer Cells through FOXO3a-Mediated Pathways	Catalase, SOD2	FOXO3a-mediated pathways
Qi D#1, Li J#1,:	2016	27777512	0	Long non-coding RNA DBCCR1-003 regulate the expression of DBCCR1 via DNMT1 in bladder cancer	DBCCR1, DNMT1	
Pinto-Leite R1,2	2016	27235118	0	mTOR inhibitors in urinary bladder cancer	PI3K, AKT, 4EBP1, elF4E	PI3K/AKT/mTOR pathway
Pereira PM1,2, :	2016	27750007	0	Mitochondria-Targeted Photodynamic Therapy with a Galactodendritic Chlorin to Enhance Cell Death in Resistant Bladder Cancer Cells	GLUT1, galectin-1	
Peixoto A1,2,3,	2016	27542232	0	Hypoxia enhances the malignant nature of bladder cancer cells and concomitantly antagonizes protein O-glycosylation extension	HIF-1α, CA-IX, epithelial markers (CDH1, EPCAM and DSP) , mesenchymal-characteristic genes (CDH2, FN1, SPARC, and VIM)	
Pawar A1, Meie	2016	27269287	0	Ral-Arf6 crosstalk regulates Ral dependent exocyst trafficking and anchorage independent growth signalling	RalA, Arf6, Ras	Ral-RalBP1-ARNO-Arf6 pathway
Park S1, Fudhai	2016	27765366	0	Cytotoxic effects of kazinol A derived from Broussonetia papyrifera on human bladder cancer cells, T24 and T24R2	cyclin D1, p21	AKT-BAD pathway and AMPK-mTOR pathway

Table S1. The list of bladder cancer related articles either used or not used RT4.

Author	Year	PMID	using RT4 or not(0 represents No;1 represents Yes)	Title	Gene	Pathway
Park BH1, Lim	2016	27564099	0	Curcumin potentiates antitumor activity of cisplatin in bladder cancer cell lines via ROS-mediated activation of ERK1/2	p53, p21, p-STAT3, caspase-3, p-MEK	ROS-mediated ERK1/2 signaling
Pang X1,2, Fu X	2016	26243398	0	Overexpression of CIP2A promotes bladder cancer progression by regulating EMT	CIP2A	
Pan XW1, Li L1	2016	26496799	0	Icaritin acts synergistically with epirubicin to suppress bladder cancer growth through inhibition of autophagy	ATG3, ATG5, ATG7, ATG12, LC3	
Pan CW1, Liu H	2016	27147566	0	JNK2 downregulation promotes tumorigenesis and chemoresistance by decreasing p53 stability in bladder cancer	JNK2, p53	
Ojha R1, Singh	2016	27474203	0	JAK-mediated autophagy regulates stemness and cell survival in cisplatin resistant bladder cancer cells	JAK, MDR1, ABCG2, ALDH1	IFN-γ mediated JAK2 and STAT3 pathway
Müller K1, Klei	2016	27736810	0	EGF receptor targeted lipo-oligocation polyplexes for antitumoral siRNA and miRNA delivery	EGF, EG5, GE11	
Mirzaei MR1, K	2016	26862520	0	Altered Expression of High Molecular Weight Heat Shock Proteins after OCT4B1 Suppression in Human Tumor Cell Lines	OCT4B1, HSP90, HSP70, HSP60 gene families	
Mirzaei MR1, A	2016	27081464	0	Down-regulation of HSP40 gene family following OCT4B1 suppression in human tumor cell lines	OCT4B1, HSP40 family gene	
McBeth L1, Nw	2016	27036026	0	Glucocorticoid receptor beta increases migration of human bladder cancer cells	FKBP51, GILZ, p21	
Matsumoto R1,2	2016	27698389	0	Aldo-keto reductase 1C1 induced by interleukin-1beta mediates the invasive potential and drug resistance of metastatic bladder cancer cells	AKR1C1	
Long Y1, Wu Z	2016	27485165	0	MicroRNA-101 inhibits the proliferation and invasion of bladder cancer cells via targeting c-FOS	c-FOS	
Liu Z1, Yokoya	2016	26921394	1	High Sensitivity of an Ha-RAS Transgenic Model of Superficial Bladder Cancer to Metformin Is Associated with approximately 240-Fold Higher Drug Concentration in Urine than Serum	AMPK/ PTEN	PI3K/mTOR pathway
Liu L1,2, Liu Y	2016	26743236	1	Synthetic Bax-Anti Bcl2 combination module actuated by super artificial hTERT promoter selectively inhibits malignant phenotypes of bladder cancer	hTERT/Bcl2	Bcl2/Bax
Liu J1, Xu R1, 2	2016	27241143	0	[Mechanisms for effect of osthole on inhibiting the growth and invasion of bladder cancer cells]	COX-2, VEGF, NF-κB	
Liu J1, Wang H	2016	27099514	1	Repression of the miR-93-enhanced sensitivity of bladder carcinoma to chemotherapy involves the regulation of LASS2	LASS2	
Lin JF1, Lin YC	2016	27143856	1	Autophagy inhibition enhances RAD001-induced cytotoxicity in human bladder cancer cells	RAD001/mTOR	
Lin J1,2, Liu Y3	2016	26427661	0	Synthetic Tet-inducible small hairpin RNAs targeting hTERT or Bcl-2 inhibit malignant phenotypes of bladder cancer T24 and 5637 cells	hTERT, Bcl-2	
Lin F1, Dong L	2016	27766041	0	An Efficient Light-Inducible P53 Expression System for Inhibiting Proliferation of Bladder Cancer Cell	p53, mcherry gene	CRISPR-Cas9 based light-inducible gene expression system

Table S1. The list of bladder cancer related articles either used or not used RT4.

Author	Year	PMID	using RT4 or not(0 represents No;1 represents Yes)	Title	Gene	Pathway
Lima L1,2,3, G	2016	27835695	0	Reference Genes for Addressing Gene Expression of Bladder Cancer Cell Models under Hypoxia: A Step Towards Transcriptomic Studies	HIF-1, HPRT, ACTB, 18S, GAPDH, TBP, B2M, SDHA	FGFR3/CCND1 and E2F3/RB1 pathways
Liao YX1, Zeng	2016	27082503	0	Silencing of RTKN2 by siRNA suppresses proliferation, and induces G1 arrest and apoptosis in human bladder cancer cells	RTKN2, MCM10, CDK2, CDC24A , CDC6	
Li X1, Wang H	2016	27485374	0	Emodin enhances cisplatin-induced cytotoxicity in human bladder cancer cells through ROS elevation and MRP1 downregulation	MDR1, MRP1, MRP2, ABCG2, CTR1, ATP7A, ATP7B	
Li X1, Liu S2.	2016	27831760	1	Suppression of HBXIP Reduces Cell Proliferation, Migration and Invasion In Vitro, and Tumorigenesis In Vivo in Human Urothelial Carcinoma of the Bladder	HBXIP	
Léger K1,2, Ho	2016	27295004	0	ARTD1 regulates cyclin E expression and consequently cell-cycle re-entry and G1/S progression in T24 bladder carcinoma cells	ARTD1, cyclin E, E2F-1, c-myc, Cdk2, p27	
Kurobe M1, Ko	2016	27930669	1	Development of RNA-FISH Assay for Detection of Oncogenic FGFR3-TACC3 Fusion Genes in FFPE Samples	FGFR/FGFR3-TACC3	
Kong C1, Zhan	2016	27846823	0	Overexpression of UNC5B in bladder cancer cells inhibits proliferation and reduces the volume of transplantation tumors in nude mice	UNC5B	
Kobayashi K1,	2016	27599396	0	Clinical significance of CD44 variant 9 expression as a prognostic indicator in bladder cancer	CD44v9, CK5/6, CK20	
Kim SH1, Ho J	2016	26966728	0	Upregulated expression of BCL2, MCM7, and CCNE1 indicate cisplatin-resistance in the set of two human bladder cancer cell lines: T24 cisplatin sensitive and T24R2 cisplatin resistant bladder cancer cell lines	PRKAR2A, PRKAR2B, CYCS, BCL2, BIRC3, DFFB, CASP6, CDK6, CCNE1, STEAP3, MCM7, ORC2, ORC5, ANAPC1, and ANAPC7, CDC7, CDC27, SKP1	
Kawasaki-Nanri	2016	27020040	1	Differential effects of adipose tissue stromal cells on the apoptosis, growth and invasion of bladder urothelial carcinoma between the superficial and invasive types	ATSCs	
Kawahara T1,2,	2016	27447553	0	ZKSCAN3 promotes bladder cancer cell proliferation, migration, and invasion	ZKSCAN3, MMP-2/MMP-9, c-myc/FGFR3, p53/PTEN	
Kashiwagi E1, I	2016	27322140	0	Androgen receptor activity modulates responses to cisplatin treatment in bladder cancer	AR, NF-κB	
Karkoulis PK1,	2016	26662567	1	17-DMAG induces heat shock protein 90 functional impairment in human bladder cancer cells: knocking down the hallmark traits of malignancy	Hsp90	
Jou YC1, Tsai Y	2016	27557492	0	Foxp3 enhances HIF-1alpha target gene expression in human bladder cancer through decreasing its ubiquitin-proteasomal degradation	Foxp3, HIF-1α, GLUT-4,-9, VEGF-A, B-, D	
Jiang Y1, Han Y	2016	26715272	0	Rab23 is overexpressed in human bladder cancer and promotes cancer cell proliferation and invasion	Rab23, FGFR3, cyclin E, c-myc, MMP-9	NF-κB signaling
Iskender B1,2,	2016	26718217	0	Inhibition of epithelial-mesenchymal transition in bladder cancer cells via modulation of mTOR signalling	mTOR, ERK 1/2, p38, MAPK, Src	mTOR signalling

Table S1. The list of bladder cancer related articles either used or not used RT4.

Author	Year	PMID	using RT4 or not(0 represents No;1 represents Yes)	Title	Gene	Pathway
Iskender B1,2, I	2016	27456363	0	Reprogramming bladder cancer cells for studying cancer initiation and progression	OCT4, SOX2, KLF4, c-MYC	
Huang B1, Zhai	2016	27904673	0	MicroRNA-206 acts as a tumor suppressor in bladder cancer via targeting YRDC	YRDC	
Hu KY1, Wang	2016	27373212	0	Targeting of MCT1 and PFKFB3 influences cell proliferation and apoptosis in bladder cancer by altering the tumor microenvironment	MCT1, PFKFB3	
Hu H1, Zhao J1	2016	26637476	0	Expression of Annexin A2 and Its Correlation With Drug Resistance and Recurrence of Bladder Cancer	annexin A2	
Gross-Cohen M	2016	26968815	0	Heparanase 2 expression inversely correlates with bladder carcinoma grade and stage	Hpa2, LOX	
Gong YQ1, Pen	2016	28101210	0	UBE2T silencing suppresses proliferation and induces cell cycle arrest and apoptosis in bladder cancer cells	UBE2T	
Gawlik-Rzemiei	2016	27478472	0	Silencing expression of the NANOG gene and changes in migration and metastasis of urinary bladder cancer cells	NANOG, MMP-2, MMP-9	
Gabig TG1, Wa	2016	27520378	1	Clostridium perfringens enterotoxin as a potential drug for intravesical treatment of bladder cancer	CPE	
Fu X1,2, Pang	2016	26209051	0	XIAP inhibitor Embelin inhibits bladder cancer survival and invasion in vitro	PI3K, p-Akt	PI3K/Akt pathway
Egawa H1, Jing	2016	26837847	0	The miR-130 family promotes cell migration and invasion in bladder cancer through FAK and Akt phosphorylation by regulating PTEN	FAK, Akt, PTEN	
Ding G1, Yu S1	2016	27158349	0	Androgen receptor (AR) promotes male bladder cancer cell proliferation and migration via regulating CD24 and VEGF	AR, CD24, VEGF, MMP9	
Deng QF1, Sun	2016	26883573	0	Cigarette smoke extract induces the proliferation of normal human urothelial cells through the NF-kappaB pathway	NF-κB, p65/p50, cyclin D1	NF-κB pathway
Coccia A1, Mos	2016	27748855	0	Extra-virgin olive oil phenols block cell cycle progression and modulate chemotherapeutic toxicity in bladder cancer cells	PARP-1	
Chung YH1, Ki	2016	27349281	0	RIP kinase-mediated ROS production triggers XAF1 expression through activation of TAp73 in casticin-treated bladder cancer cells	MAPK, TAp73, XAF1, TAp63, RIP	TAp73-induced XAF1 apoptosis signaling pathway
Péchery A1, Fa	2016	27638828	1	Apoptotic effect of the selective PPARbeta/delta agonist GW501516 in invasive bladder cancer cells	ROS	
Choi EO1, Park	2016	27571890	0	Baicalein induces apoptosis via ROS-dependent activation of caspases in human bladder cancer 5637 cells	cIAP-1, cIAP-2, DR4, DR5, MMP	
Choi BH1, Lee	2016	27706018	0	Controls of Nuclear Factor-Kappa B Signaling Activity by 5'-AMP-Activated Protein Kinase Activation With Examples in Human Bladder Cancer Cells	AMPK, NF-κB	NF-κB signaling
Chiu KY1, Wu	2016	26679052	0	Inhibition of growth, migration and invasion of human bladder cancer cells by antrocin, a sesquiterpene lactone isolated from Antrodia cinnamomea, and its molecular mechanisms	MMPs, ERK, c-Fos, FAK	FAK-paxillin and ERK-c-Fos-MMP2 signaling pathways

Table S1. The list of bladder cancer related articles either used or not used RT4.

Author	Year	PMID	using RT4 or not(0 represents No;1 represents Yes)	Title	Gene	Pathway
Chen J1, Wang	2016	26733306	0	Maspin enhances cisplatin chemosensitivity in bladder cancer T24 and 5637 cells and correlates with prognosis of muscle-invasive bladder cancer patients receiving cisplatin based neoadjuvant chemotherapy	AKT, PI3K, mTOR, Bcl-2, Caspase3	PI3K/ AKT/mTOR signal passway
Chen DJ1, Chen	2016	27660415	0	Downregulation of DOCK1 sensitizes bladder cancer cells to cisplatin through preventing epithelial-mesenchymal transition	DOCK1	
Chuang CH et a	2015	26087450	1	Lab on a chip for multiplexed immunoassays to detect bladder cancer using multifunctional dielectrophoretic manipulations	Gal- 1,LDH-B,	
Cogoi S et al.	2015	26057859	0	Potent Apoptotic Response Induced by Chloroacetamidine Anthrathiophenediones in Bladder Cancer Cells	HRAS,PARP-1,caspases 3/7,annexin 5,cyclin D1,p21,compound 3a,compound 3b	
Conde VR et al.	2015	25907297	1	The progression from a lower to a higher invasive stage of bladder cancer is associated with severe alterations in glucose and pyruvate metabolism	GLUT1,GLUT3,MCT4,PFK1,GPT,LDH	
Cui M et al.	2015	25681288	1	Intravenous siRNA Silencing of Survivin Enhances Activity of Mitomycin C in Human Bladder RT4 Xenografts	MMC,Survivin，HLA，	
de Souza D et a	2015	25811955	0	New organochalcogen multitarget drug: synthesis and antioxidant and antitumoral activities of chalcogenozidovudine derivatives	BAX,Bcl-2,caspase9,survivin	
Zhuang CL et a	2015	25775949	0	Synthetic miRNA sponges driven by mutant hTERT promoter selectively inhibit the progression of bladder cancer	hTERT、Caspase3	
Zhu H et al.	2015	25701463	0	Downregulation of UPK1A suppresses proliferation and enhances apoptosis of bladder transitional cell carcinoma cells	UPK1A	
Zhu H et al.	2015	26449463	0	Long non-coding RNA ANRIL is up-regulated in bladder cancer and regulates bladder cancer cell proliferation and apoptosis through the intrinsic pathway	Bax、Bcl-xl、Caspase3、Caspase8、Caspase9、PARP、Smac、cytc	intrinsic pathway
Zhou X U et al.	2015	26722309	1	miR-128 downregulation promotes growth and metastasis of bladder cancer cells and involves VEGF-C upregulation	VEGF-C	
Zheng Y et al.	2015	26322830	0	Compound A Inhibits Bladder Cancer Growth Predominantly via Glucocorticoid Receptor Transrepression	NF-kB、GR、GILZ、FKBP51、MMP2、MMP9、IL-6、VEGF	AR pathway
Zhao X et al.	2015	26396669	0	MiRNA-29c regulates cell growth and invasion by targeting CDK6 in bladder cancer	CDK6	
Zhao X et al.	2015	26396669	0	MiRNA-125b inhibits proliferation and migration by targeting SphK1 in bladder cancer	SPHK1	
Zhao L et al.	2015	25757908	0	Benzidine induces epithelial-mesenchymal transition in human uroepithelial cells through ERK1/2 pathway	Vimentin、E-cadherin、ZO-1、ERK1/2、P38、JNK、MMP2、AP-1	ERK1/2 pathway、e p38 MAPK pathway
Zhang Z W et a	2015	26521794	1	Caffeine Suppresses Apoptosis of Bladder Cancer RT4 Cells in Response to Ionizing Radiation by Inhibiting Ataxia Telangiectasia Mutated-Chk2-p53 Axis	p53、PUMA、p21、Bax、γH2AX、ATM、ATR	ATM-Chk2-p53-Puma DNA damage-signaling axis
Zhang X et al.	2015	26599571	0	MicroRNA-203 Is a Prognostic Indicator in Bladder Cancer and Enhances Chemosensitivity to Cisplatin via Apoptosis by Targeting Bcl-w and Survivin	Bcl-w、Survivin	

Table S1. The list of bladder cancer related articles either used or not used RT4.

Author	Year	PMID	using RT4 or not(0 represents No;1 represents Yes)	Title	Gene	Pathway
Zhang X et al.	2015	26473854	0	G Protein-Coupled Receptor 87 (GPR87) Promotes Cell Proliferation in Human Bladder Cancer Cells	GPR87、p53、p21、ERK1/2、AKT 、MEK1/2、PI3K	MAPK pathway、p53 Pathway
Zhang T et al.	2015	25585815	0	The ATM inhibitor KU55933 sensitizes radioresistant bladder cancer cells with DAB2IP gene defect	DAB2IP、ATM、DNA-PKcs、Rad51 、CHK1、SMC1、 TIF1-β	
Zhang T et al.	2015	25865820	0	c-Fos is involved in inhibition of human bladder carcinoma T24 cells by brazilin	c-Fos	
Zhang Q et al.	2015	26484567	0	Honokiol inhibits bladder tumor growth by suppressing EZH2/miR-143 axis	EZH2、MMP9、 CD44、 Sox2、 cyclinD1	EZH2/miR-143 axis
Zhang Y et al.	2015	26541358	0	Synthetic Tet-inducible artificial microRNAs targeting β-catenin or HIF-1α inhibit malignant phenotypes of bladder cancer cells T24 and 5637	HIF-1α、β -catenin	
Zeng T et al.	2015	25797626	0	IRE1alpha-TRAF2-ASK1 complex-mediated endoplasmic reticulum stress and mitochondrial dysfunction contribute to CXC195-induced apoptosis in human bladder carcinoma T24 cells	GRP78、 CHOP、 IRE1α、 TRAF2、 ASK1、JNK、	IRE1α-TRAF2-ASK1
Zeng J et al.	2015	26550193	0	Role of WISP3 siRNA in proliferation, apoptosis and invasion of bladder cancer cells	WISP3、Caspase3、Caspase9	
Yuge K et al.	2015	26184482	0	Nicotine Induces Tumor Growth and Chemoresistance through Activation of the PI3K/Akt/mTOR Pathway in Bladder Cancer	AKT、pS6	PI3K/Akt/mTOR Pathway
Yu X et al.	2015	25691904	0	Human adipose derived stem cells induced cell apoptosis and s phase arrest in bladder tumor	caspase-3、PARP、Bax、Bcl-2、Akt 、PTEN	PTEN/PI4K/Akt pathway
Yu DS et al.	2015	26228277	0	Bacille Calmette-Guerin can induce cellular apoptosis of urothelial cancer directly through toll-like receptor 7 activation	TLR7、ATG2、LC3、IRAK2、 IRAK4、caspase 8	
Yeh H H et al.	2015	25885317	0	Ras induces experimental lung metastasis through up-regulation of RbAp46 to suppress RECK promoter activity	Ras、RECK、MMP-9、RbAp46 、 HDAC1 、 SP1	
Yeh CR et al.	2015	26045993	0	Fibroblast ERalpha promotes bladder cancer invasion via increasing the CCL1 and IL-6 signals in the tumor microenvironment	CCL1、IL-6、Erα、CCL5、CCL11、 CXCL1、CXCL7	
Yang Y et al.	2015	25973680	0	RGD-modified oncolytic adenovirus exhibited potent cytotoxic effect on CAR-negative bladder cancer-initiating cells	caspase 8、caspase 3、PARP、TRAIL 、survivin、Nanog、MRP1	
Yang X et al.	2015	25266796	0	A lentiviral sponge for miRNA-21 diminishes aerobic glycolysis in bladder cancer T24 cells via the PTEN/PI3K/AKT/mTOR axis	GLUT1、GLUT3、 LDHA、LDHB、 HK1、 HK2、 PKM、HIF-1α、PTEN 、AKT、mTOR	PTEN/PI3K/AKT/Mtor
Yang T et al.	2015	25887782	1	Huachansu suppresses human bladder cancer cell growth through the Fas/Fasl and TNF- alpha/TNFR1 pathway in vitro and in vivo	Fas、Fasl、TNFa、TNFR1、 Caspase-3、Caspase-8、Caspase-9、 PARP、 p65、iκB-α、Bax、Bcl-2、XIAP	Fas/Fasl,TNF- alpha/TNFR1 pathway、NF-κB pathway
Yang G et al.	2015	25536294	0	Quantitative glycome analysis of N-glycan patterns in bladder cancer vs normal bladder cells using an integrated strategy	Jacalin、 LTL、 LCA、 PTL-II、 NPA、 LEL、 SJA、Con A	
Yan Z et al.	2015	25695283	0	Adenovirus-mediated LRIG1 expression enhances the chemosensitivity of bladder cancer cells to cisplatin	LRIG1、EGFR、Bcl-2、Bax	
Xue Y et al.	2015	25514464	0	A novel antisense long noncoding RNA regulates the expression of MDC1 in bladder cancer	MDC1	

Table S1. The list of bladder cancer related articles either used or not used RT4.

Author	Year	PMID	using RT4 or not(0 represents No;1 represents Yes)	Title	Gene	Pathway
Xue M et al.	2015	25824695	0	Hypoxia regulates the expression and localization of CCAAT/enhancer binding protein alpha by hypoxia inducible factor-1alpha in bladder transitional carcinoma cells	C/EBPα、HIF-1α	
Xue D et al.	2015	25846736	0	Clinical significance and biological roles of TRIM24 in human bladder carcinoma	cyclin D1、 cyclin E、IκBα、AKT、TRIM24	NF-κB pathways，AKT signaling pathways
Xu S T et al.	2015	25591934	0	Role of osteopontin in the regulation of human bladder cancer proliferation and migration in T24 cells	OPN、P53、Caspase-3、Caspase-8、Caspase-9	
Xu S T et al.	2015	26339359	0	Targeting MACC1 by RNA interference inhibits proliferation and invasion of bladder urothelial carcinoma in T24 cells	MACC1、 caspase-3、 caspase-8、Bax、 Met	
Xie D et al.	2015	25596753	0	Up-regulation of miR-9 target CBX7 to regulate invasion ability of bladder transitional cell carcinoma	CBX7	
Wen J et al.	2015	25745360	0	Human urothelial carcinoma cell response to Sunitinib malate therapy in vitro	Fas、 FasL、 PARP	Fas/Fasl pathway
Wen J et al.	2015	25837361	0	Effects of sunitinib malate on growth of human bladder transitional cell line T24 in vitro	Fas、 FasL、 PARP	Fas/Fasl pathway
Wei S et al.	2015	26381881	0	Silencing of ATPase Inhibitory Factor 1 Inhibits Cell Growth via Cell Cycle Arrest in Bladder Cancer	IF1、cdk2、cyclin D、cdk4、cdk6.	
Wei B et al.	2015	25326807	0	Cripto-1 expression and its prognostic value in human bladder cancer patients	Cripto-1	
Wang Y W.et al	2015	25569706	0	Antiangiogenesis as the novel mechanism for justicidin A in the anticancer effect on human bladder cancer	VEGF、PDGF-A、TF、MT-MMP、MMP-2、MMP-9	
Wang Y et al.	2015	25790869	0	Reduced ING4 Expression Is Associated with the Malignancy of Human Bladder	ING4	
Wang P et al.	2015	26397392	0	Licochalcone C induces apoptosis via B-cell lymphoma 2 family proteins in T24 cells	PARP、caspase-3、 Bcl-2 、Bax、Bim、 Bcl-w、 Bcl-XL	
Wang H et al.	2015	26150338	0	miR-9 promotes cell proliferation and inhibits apoptosis by targeting LASS2 in bladder cancer	LASS2、cyclin D1、 MMP9、 Bcl-2 、E-cadherin 、BAX、Survivin	
Wang D et al.	2015	26622608	0	In vitro and in vivo targeting of bladder carcinoma with metformin in combination with cisplatin	mTOR、AMPK	AKT/mTOR pathway
Wang C et al.	2015	25918708	0	A New Double Stranded RNA Suppresses Bladder Cancer Development by Upregulating p21 (Waf1/CIP1) Expression	P21、Cyclin D1、 CDK4、 CDK6、CyclinA2	
Varol N et al.	2015	25349215	0	Does Wnt/beta-catenin pathway contribute to the stability of DNMT1 expression in urological cancer cell lines?	DNMT1、b-catenin、pGSK3b、HAUSP、UHRF1	Wnt/β-catenin signaling pathway
Tsui K H et al.	2015	26249737	1	Growth differentiation factor-15: a p53- and demethylation-upregulating gene represses cell proliferation, invasion, and tumorigenesis in bladder carcinoma cells	GDF15、p53、SNAIL、SLUG、 E-cadherin、 N-cadherin、MASPIN、NDRG1、NDRG3	
Tian Y F et al.	2015	26107200	0	OK-432 Suppresses Proliferation and Metastasis by Tumor Associated Macrophages in Bladder Cancer	PCNA、E-Cadherin、 Vimentin、snail	
Tian D et al.	2015	26129954	0	Expression of brainspecific angiogenesis inhibitor1 and association with p53, microvessel density and vascular endothelial growth factor in the tissue of human bladder transitional cell carcinoma	BAI-1、VEGF、p53	

Table S1. The list of bladder cancer related articles either used or not used RT4.

Author	Year	PMID	using RT4 or not(0 represents No;1 represents Yes)	Title	Gene	Pathway
Taoka Y et al.	2015	26299484	0	Protein expression profile related to cisplatin resistance in bladder cancer cell lines detected by two-dimensional gel electrophoresis	HNRNPA3、PCK2、PPL、PGK1、TKT、SERPINB2、GOT2、EIF3A	
Tang Y et al.	2015	26136904	0	Afatinib inhibits proliferation and invasion and promotes apoptosis of the T24 bladder cancer cell line	Bcl-2、Bax、Akt、ERK1/2、MMP-2、MMP-9	The PI3K/Akt pathways, MAPK/ERK pathways
Takeuchi H et a	2015	26323344	0	Sequential gemcitabine and tamoxifen treatment enhances apoptosis and blocks transformation in bladder cancer cells	Erα、Erβ、PARP、caspase-3、p70S6K	
Sun M et al.	2015	25434982	0	Lentinan reduces tumor progression by enhancing gemcitabine chemotherapy in urothelial bladder cancer	Bcl-2、survivin	
Sun H et al.	2015	25878394	0	miR-34a inhibits proliferation and invasion of bladder cancer cells by targeting orphan nuclear receptor HNF4G	HNF4G	
Sun D et al.	2015	25499922	0	MK2206 potentiates cisplatin-induced cytotoxicity and apoptosis through an interaction of inactivated Akt signaling pathway	Akt、4EBP1、GSK3β、mTOR、70S6K、S6RP、ZEB1、Slug、Snail、Bax、Survivin、Caspase-8、Caspase-9、Caspase-3、mTOR	Akt signaling pathway
Su C C et al.	2015	25967669	1	Cantharidin Induces Apoptosis Through the Calcium/PKC-Regulated Endoplasmic Reticulum Stress Pathway in Human Bladder Cancer Cells	Caspase-7、Caspase-9、Caspase-3、Caspase-12、eIF-2、PKC、Grp78	
Smolensky D. e	2015	26597249	0	Phosphatidylinositol- 3-kinase inhibitor induces chemosensitivity to a novel derivative of doxorubicin, AD198 chemotherapy in human bladder cancer cells in vitro	PARP、AKT、GSK-3β、ERK1/2	PI3K/AKT signaling pathway, p38 MAPK pathway
Shu J. et al.	2015	25564356	0	Downregulation of angiogenin inhibits the growth and induces apoptosis in human bladder cancer cells through regulating AKT/mTOR signaling pathway	ANG、AKT、GSK-3β、mTOR、Bcl-2、Bax、Caspase-3、4E-BP1、p70S6	AKT/mTOR signaling pathway
Shi H et al.	2015	26125467	0	Overexpression of monocarboxylate anion transporter 1 and 4 in T24-induced cancer-associated fibroblasts regulates the progression of bladder cancer cells in a 3D microfluidic device	a-SMA、CD34、MCT1、MCT4	
Savio A L. et al.	2015	25771977	1	Inhibition of bladder cancer cell proliferation by allyl isothiocyanate	ANLN、S100P、SMAD4、BAX, BCL2、CDK1	
Sabbineni H. et	2015	26148825	0	Genetic deletion and pharmacological inhibition of Akt1 isoform attenuates bladder cancer cell proliferation, motility and invasion	Akt1、Akt2、Akt3	
Rogler A et al.	2015	25732201	0	Functional analyses and prognostic significance of SFRP1 expression in bladder cancer	SFRP1、AXIN2、BMP4、CD44、CMYC、CYCLIND1、SURVIVIN.	Wnt signaling pathway
Rieger C et al.	2015	25595385	0	Characterization of different carbon nanotubes for the development of a mucoadhesive drug delivery system for intravesical treatment of bladder cancer	CNT-1、CNT-2、CNT-3、CNT-4	
Rieger C et al.	2015	26201840	0	Antisense- and siRNA-mediated inhibition of the anti-apoptotic gene Bcl-xL for chemosensitization of bladder cancer cells	Bcl-xL、BX713、BAX	
Resnik N et al.	2015	26361392	1	Highly Selective Anti-Cancer Activity of Cholesterol-Interacting Agents Methyl-beta-Cyclodextrin and Ostreolysin A/Pleurotolysin B Protein Complex on Urothelial Cancer Cells	OlyA、PARP、LC3-I、LC3-II	

Table S1. The list of bladder cancer related articles either used or not used RT4.

Author	Year	PMID	using RT4 or not(0 represents No;1 represents Yes)	Title	Gene	Pathway
Qu H et al.	2015	26276292	0	Effect of salinomycin on metastasis and invasion of bladder cancer cell line T24	E-cadherin、vimentin	
Pegas Henrique:	2015	25524242	0	Expression and Function of LPA1 in Bladder Cancer	LPA1、LPA2、LPA3、ATX、MYPT1、MLC	
Papadopoulos E	2015	25833690	0	Gemcitabine impacts differentially on bladder and kidney cancer cells: distinct modulations in the expression patterns of apoptosis-related microRNAs and BCL2 family genes	BCL2、 BAX、 BCL2L12	
Papadopoulos E	2015	26356996	0	Cisplatin and Paclitaxel Alter the Expression Pattern of miR-143/145 and miR-183/96/182 Clusters in T24 Bladder Cancer Cells	RNU48、AKT、PAI-1、COX-2、ERK5、ILK、CBFB、CLINT1、PPP3CA、FSCN1、SOCS7、PAK1、IGF-IR、NR、IRS1、MAP4K1、FOXO1、SMAD4、RECK	
Pan Q et al.	2015	26245871	0	Metformin can block precancerous progression to invasive tumors of bladder through inhibiting STAT3-mediated signaling pathways	Bcl2、 BAX、cyclin D1、STAT3	STAT3 pathway
Olbert PJ et al.	2015	25499923	0	TLR4- and TLR9-dependent effects on cytokines, cell viability, and invasion in human bladder cancer cells	TLR4、TLR9、IL-6、IL-8、TNFα、INFβ	
Nowak J M.et a	2015	25544037	0	Gelsolin is a potential cellular target for cotinine to regulate the migration and apoptosis of A549 and T24 cancer cells	F-actin、gelsolin	
Mu D W et al.	2015	26823699	0	Oleanolic acid suppresses the proliferation of human bladder cancer by Akt/mTOR/S6K and ERK1/2 signaling	AKT、mTOR、S6K、ERK1/2	Akt/mTOR/S6K pathway、 ERK1/2 pathway
Mo M et al.	2015	25798926	0	CCL21/CCR7 enhances the proliferation, migration, and invasion of human bladder cancer T24 cells	CCL21、CCR7、MMP-2、 MMP-9、Bcl-2、<br>Bax、VEGF-C、HuR	
Miglietta G et al	2015	26674223	0	Nucleic Acid Targeted Therapy: G4 Oligonucleotides Downregulate HRAS in Bladder Cancer Cells through a Decoy Mechanism	p21、CTRL-1、CTRL-2、CTRL-3	
Matsumoto R et	2015	25816892	1	Adaptor protein CRK induces epithelial-mesenchymal transition and metastasis of bladder cancer cells through HGF/c-Met feedback loop	CRK-I、CRK-II、MIB-1、MMP9、E-cadherin、 N-cadherin、vimentin、Zeb1、ERK 、Akt	Akt signaling pathway、HGF/c-Met feedback loop
Mani J et al.	2015	25885284	1	Chemoresistance is associated with increased cytoprotective autophagy and diminished apoptosis in bladder cancer cells treated with the BH3 mimetic (-)-Gossypol (AT-101)	Bcl-xL、Mcl-1、ATG5、Bax、Bak、p62、LC3	
Lv L et al.	2015	25444900	0	MiR-193a-3p promotes the multi-chemoresistance of bladder cancer by targeting the HOXC9 gene	HOXC9、ACTB、CDKN1A、EDN1、RelA、TERT、ODC1、NQO1、ECSIT、HO-1、Nrf-2	DNA damage response、 Notch、NF-κB、 Myc/Max and Oxidative Stress
Lu H et al.	2015	26046128	0	DUOX2 promotes the elimination of the Klebsiella pneumoniae strain K5 from T24 cells through the reactive oxygen species pathway	IFN-γ、DUOX2、TNF-α、 IL-1β、IL-17、PMA 、MDP	
Liu L et al.	2015	25934337	0	Inducing cell growth arrest and apoptosis by silencing long non-coding RNA PCAT-1 in human bladder cancer	PCAT-1	

Table S1. The list of bladder cancer related articles either used or not used RT4.

Author	Year	PMID	using RT4 or not(0 represents No;1 represents Yes)	Title	Gene	Pathway
Liang K et al.	2015	25323066	0	KLF8 is required for bladder cancer cell proliferation and migration	KLF8、E-Cadherin、 Vimentin	
Li Y et al.	2015	25991669	0	The miR-193a-3p-regulated ING5 gene activates the DNA damage response pathway and inhibits multi-chemoresistance in bladder cancer	ING5、LOXL4、SRSF2、 HIC2、PLAU、 LOXL4、 PSEN1、HOXC9	DNA damage, NF-κB and Myc/Max pathways
Li Y et al.	2015	26617702	0	Expression of Robo protein in bladder cancer tissues and its effect on the growth of cancer cells by blocking Robo protein	Robo1、Robo4	
Li W et al.	2015	25722217	0	Evaluation of transforming growth factor-beta1 suppress Pokemon/epithelial-mesenchymal transition expression in human bladder cancer cells	TGF-β1、MMP2、 MMP9、 Twist、VEGF、β-catenin	
Li S et al.	2015	26163939	0	The YAP1 oncogene contributes to bladder cancer cell proliferation and migration by regulating the H19 long noncoding RNA	YAP1	
Li G et al.	2015	27352332	0	Ectopic WWOX Expression Inhibits Growth of 5637 Bladder Cancer Cell In Vitro and In Vivo	WWOX、caspase-3、PARP、Bcl-2、BAX	
Li F et al.	2015	26397365	0	G9a Inhibition Induces Autophagic Cell Death via AMPK/mTOR Pathway in Bladder Transitional Cell Carcinoma	G9a 、 p62、LC-3 I/II 、H3K9me2、AMPK、ACC、-Raptor、mTOR、S6K、4E-BP1	AMPK/mTOR Pathway
Lei Y et al.	2015	25658842	0	miR-101 suppresses vascular endothelial growth factor C that inhibits migration and invasion and enhances cisplatin chemosensitivity of bladder cancer cells	VEGF、	
Lan G et al.	2015	26170849	0	MicroRNA-490-5p is a novel tumor suppressor targeting c-FOS in human bladder cancer	c-FOS、TET1、VRAG	
Kuwada M et al	2015	26342407	1	Pro-chemotherapeutic effects of antibody against extracellular domain of claudin-4 in bladder cancer	CLDN4、CL4、ACTB、MMP9、EGFR、ERK1/2	
Konstantakou E	2015	26198749	1	3-BrPA eliminates human bladder cancer cells with highly oncogenic signatures via engagement of specific death programs and perturbation of multiple signaling and metabolic determinants	ICAD、caspase-3、 caspase-8、caspase-9、PARP、ATG5、ATG7、ATG12、Beclin-1 、LC3B、P53、GSK-3β、AKT、mTOR、FoxO1、FoxO3、p38	AkT pathway、 MAPKpathway
Konac E et al.	2015	26171069	0	Synergistic effects of cisplatin and proteasome inhibitor bortezomib on human bladder cancer cells	casp-3、 casp-8、casp-9、Bcl-2、Bcl-xL、Bim、Bik	
Konac E et al.	2015	26178977	0	LINE-1 hypomethylation induced by reactive oxygen species is mediated via depletion of S-adenosylmethionine	LINE-1、TA、NAC、SAM	
Kim G Y et al.	2015	26246284	0	Gecko proteins induce the apoptosis of bladder cancer 5637 cells by inhibiting Akt and activating the intrinsic caspase cascade	Akt 、 caspase 9 、 caspase 3、 caspase 7	AkT pathway
Kim G Y et al.	2015	26612629	0	Erratum to: Gecko proteins induce the apoptosis of bladder cancer 5637 cells by inhibiting Akt and activating the intrinsic caspase cascade	Akt 、 caspase 9 、 caspase 3、 caspase 8	AkT pathway
Jin H et al.	2015	25402510	0	Divergent behaviors and underlying mechanisms of cell migration and invasion in non-metastatic T24 and its metastatic derivative T24T bladder cancer cell lines	CDC42、Rac1、SOD2、RhoA、MMP2、VEGF、NK/c-Jun、ERK、P38	MAPK pathway

Table S1. The list of bladder cancer related articles either used or not used RT4.

Author	Year	PMID	using RT4 or not(0 represents No;1 represents Yes)	Title	Gene	Pathway
Jin D et al.	2015	25388838	0	Catalpol Inhibited the Proliferation of T24 Human Bladder Cancer Cells by Inducing Apoptosis Through the Blockade of Akt-Mediated Anti-apoptotic Signaling	PARP、caspase 3、 Bax、 Bad、 Bcl-2、Bcl-xl、Akt、 PI3K、PDK	AkT pathway
Jiang Z et al.	2015	25752336	0	Reduction of protein kinase C alpha (PKC-alpha) promote apoptosis via down-regulation of Dicer in bladder cancer	PKC-a、PARP、 DICER、Caspase-3 、	
Jiang L et al.	2015	26459851	0	Rhoassociated kinase inhibitor, Y27632, inhibits the invasion and proliferation of T24 and 5367 bladder cancer cells	MLCK	
Ito Y et al.	2015	25926105	0	Down-regulation of NF kappa B activation is an effective therapeutic modality in acquired platinum-resistant bladder cancer	p65、Ki-67、NF-κB、Bcl-2、survivin	NF-κB pathway
Huang W et al.	2015	26090392	0	Roles of ERbeta and GPR30 in Proliferative Response of Human Bladder Cancer Cell to Estrogen	Erβ、GPR30、c-FOS、cyclinD1	EGFR-MAPK pathway
Huang J et al.	2015	26239274	0	NMyc downstreamregulated gene 2 suppresses the proliferation of T24 human bladder cancer cells via induction of oncosis	NDRG2、	
Ho I L et al.	2015	24925635	0	Nucleophosmin1 associated with drug resistance and recurrence of bladder cancer	NPM1	
Heubach J et al.	2015	26592553	0	MLN4924 Synergistically Enhances Cisplatin-induced Cytotoxicity via JNK and Bcl-xL Pathways in Human Urothelial Carcinoma	JNK、ATM、Bcl-xL、Bcl-2、caspase 3、caspase7、PARP、ERK、P38、ATR、BRCA1	JNK and Bcl-xL Pathways；ERK1/2 and p38 pathways.
Hensel J et al.	2015	26078295	0	Patient Mutation Directed shRNA Screen Uncovers Novel Bladder Tumor Growth Suppressors	PCIF1、 SAMD9L、 IQGAP1、 MED1、 KATNAL1、TGFBR2	
Hattori S et al.	2015	26118200	0	Improved Target Cell Selection and Counting Method for UroVysion Fluorescence in Situ Hybridization	CK7、PCNA、DAPI	
Gao D et al.	2015	26503336	0	CMTM8 inhibits the carcinogenesis and progression of bladder cancer	CMTM8、	
Gan Y et al.	2015	25796505	0	Knockdown of HMGN5 suppresses the viability and invasion of human urothelial bladder cancer 5637 cells in vitro and in vivo	HMGN5、 cyclinD1、E-cadherin、VEGF-C	
Gan Y et al.	2015	25796505	0	Erratum to: Knockdown of HMGN5 suppresses the viability and invasion of human urothelial bladder cancer 5638 cells in vitro and in vivo	HMGN5、 cyclinD2、E-cadherin、VEGF-C	
Fujii T et al.	2015	26514209	0	microRNA-145 promotes differentiation in human urothelial carcinoma through down-regulation of syndecan-1	Syndecan-1、p63、CK5、MUC-1、MUC-2、MUC-5、NSE、UCHL-1、SOX2、NANOG、E2F3、CD44、p53、E-cadherin、SA-β-gal 、PAP、	
Fu X et al.	2015	25800227	0	Synthetic artificial microRNAs targeting UCA1-MALAT1 or c-Myc inhibit malignant phenotypes of bladder cancer cells T24 and 5637	UCA1-MALAT1、c-Myc、caspase-3	
Fu B et al.	2015	25585941	0	MiR-221-induced PUMA silencing mediates immune evasion of bladder cancer cells	Bax、Bcl-2、PUMA、MMP-9、VEGF-C	
Franzen C A et a	2015	26280654	0	Urothelial cells undergo epithelial-to-mesenchymal transition after exposure to muscle invasive bladder cancer exosomes	E-cadherin、α-SMA、β-catenin、vimentin, S100A4、snail、twist	

Table S1. The list of bladder cancer related articles either used or not used RT4.

Author	Year	PMID	using RT4 or not(0 represents No;1 represents Yes)	Title	Gene	Pathway
Du Y et al.	2015	25496438	0	G-protein-coupled receptor 137 accelerates proliferation of urinary bladder cancer cells in vitro	GPR137	
Du H F et al.	2015	26192362	0	Expression of hepaCAM inhibits bladder cancer cell proliferation via a Wnt/beta-catenin-dependent pathway in vitro and in vivo	b-catenin、 hepaCAM、c-Myc、cyclinD1、GSK3b、AKT	Wnt/β-catenin-dependent pathway，AKT pathway
Deng H et al.	2015	25542424	0	The miR-193a-3p regulated PSEN1 gene suppresses the multi-chemoresistance of bladder cancer	PSEN1、ODC1、TERT、ACTB、CDKN1A	NF-κB pathway，Notch pathway,DNA damage, Myc/Max pathways
Ou, Y.	2014	24140250	0	Activation of cyclic AMP/PKA pathway inhibits bladder cancer cell invasion by targeting MAP4-dependent microtubule dynamics	MAP4	cAMP/PKA/MAP4
Fu, L.	2014	24935471	0	ADAM10 regulates proliferation, invasion, and chemoresistance of bladder cancer cells	ADAM10	
Cho, T. M.	2014	24333868	0	AKT signaling is involved in fucoidan-induced inhibition of growth and migration of human bladder cancer cells	Cyclin D1\Cyclin E\CDK2\CDK4\P21\P27\P53\P38\ERK \JNK\AKT\	AKT
Liu, J.	2014	24388773	0	alpha4 contributes to bladder urothelial carcinoma cell invasion and/or metastasis via regulation of E-cadherin and is a predictor of outcome in bladder urothelial carcinoma patients	α4、	
Yamasaki, M.	2014	24426186	0	alpha-Lipoic acid suppresses migration and invasion via downregulation of cell surface beta1-integrin expression in bladder cancer cells	FAK\p-akt	erk\PI3K/Akt
Mirzaei, M. R.	2014	25008565	0	Altered expression of apoptotic genes in response to OCT4B1 suppression in human tumor cell lines	OCT4B1	
Zhao, B.	2014	24700345	0	Altholactone induces reactive oxygen species-mediated apoptosis in bladder cancer T24 cells through mitochondrial dysfunction, MAPK-p38 activation and Akt suppression	Pakt\Akt\p38\bax\bcl-2\	MAPK-p38
Lourenco, L. M	2014	24943806	0	Amphiphilic phthalocyanine-cyclodextrin conjugates for cancer photodynamic therapy	a-CD\b-CD\r-CD	
Jitao, W.	2014	24329492	0	Androgen receptor inducing bladder cancer progression by promoting an epithelial-mesenchymal transition	AR\E-cadherin, b-catenin, N-cadherin	
Rathore, K.	2014	24964787	0	Animal model of naturally occurring bladder cancer: characterization of four new canine transitional cell carcinoma cell lines	PDGFR, EGFR, VEGFR, p-ERK1/2, COX-2, p65, cyclin D1,P27	
Otterlei, M.	2014	25099010	0	Antimetastatic effects of licochalcone B on human bladder carcinoma T24 by inhibition of matrix metalloproteinases-9 and NF-small ka, CyrillicB activity	MMP-2,MMP-9,LCB	NF-Kb
Braesch-Anders	2014	25513803	0	ApoD mediates binding of HDL to LDL and to growing T24 carcinoma	ApoD,D544,ldl17,E981,H219,LDL20 ,J29,	
Srivastava, A. K	2014	25123267	0	Appraisal of diagnostic ability of UCA1 as a biomarker of carcinoma of the urinary bladder	UCA1	

Table S1. The list of bladder cancer related articles either used or not used RT4.

Author	Year	PMID	using RT4 or not(0 represents No;1 represents Yes)	Title	Gene	Pathway
Liu, S.	2014	25257954	0	Arsenic induced overexpression of inflammatory cytokines based on the human urothelial cell model in vitro and urinary secretion of individuals chronically exposed to arsenic	TGF-α\TNF-α\IL-8\	
Laurent, V. M.	2014	24857933	0	Atomic force microscopy reveals a role for endothelial cell ICAM-1 expression in bladder cancer cell adherence	CD43	
Du, L.	2014	24650577	0	Autophagy inhibition sensitizes bladder cancer cells to the photodynamic effects of the novel photosensitizer chlorophyllin e4	LC3\P62\Beclin1\	
Ojha, R.	2014	25020236	0	Autophagy inhibition suppresses the tumorigenic potential of cancer stem cell enriched side population in bladder cancer	SOX-2\NANOG, KLF-4, OCT-4, ABCG2, and MDR1,LC3\Bcl-2, Bcl-XL, c-IAP1/2 and XIAP\	
Li, C.	2014	25002124	0	BCMab1, a monoclonal antibody against aberrantly glycosylated integrin alpha3beta1, has potent antitumor activity of bladder cancer in vivo	BCMab1,a3, Talin1, and Kindlin2 ,a3b1,GALNT1	
Bao, Y.	2014	25532034	0	Benefits and risks of the hormetic effects of dietary isothiocyanates on cancer prevention	Nrf2,GPx2	NF-kB, FOXO, HIF, Nrf2,
Gerhardt, D.	2014	24239461	0	Boldine induces cell cycle arrest and apoptosis in T24 human bladder cancer cell line via regulation of ERK, AKT, and GSK-3beta	ERK,GSK-3β,AKT	
Begnini, K. R.	2014	25530785	0	Brazilian red propolis induces apoptosis-like cell death and decreases migration potential in bladder cancer cells	caspase-9, caspase-8, caspase-3, and p53,Bcl-2,	Bax/Bcl-2
Liang, W.	2014	24968949		CAV-1 contributes to bladder cancer progression by inducing epithelial-to-mesenchymal transition	CAV-1	PI3K/AKT
Savio, A. L.	2014	24625788	1	Cell cycle kinetics, apoptosis rates, DNA damage and TP53 gene expression in bladder cancer cells treated with allyl isothiocyanate (mustard essential oil)	TP53	p53
Zheng, X.	2014	24626525	0	Cholera toxin, a typical protein kinase A activator, induces G1 phase growth arrest in human bladder transitional cell carcinoma cells via inhibiting the c-Raf/MEK/ERK signaling pathway	PKA,p-c-RafSer43, p-c-RafSer259 , p-c-RafSer621 , c-Raf, p-Mek1/2Ser217/221, p-Erk1/2Thr202/Tyr204 , Mek and Erk	c-Raf/MEK/ERK
Man, X.	2014	24443255	0	Clinical significance and biological roles of CARMA3 in human bladder carcinoma	CARMA3/cyclin D1 and Bcl-2/	NF-κB
Han, B.	2014	24375195	0	Clinical significance and biological roles of CRKL in human bladder carcinoma	CRKL	
Becker, M. N.	2014	24054871	0	The combination of an mTORc1/TORc2 inhibitor with lapatinib is synergistic in bladder cancer in vitro	pEGFR,pAktS473,pAkt T308	mTOR,EGFR/HER2
Wang, L.	2014	25085582	0	Combination of bladder cancer-specific oncolytic adenovirus gene therapy with cisplatin on bladder cancer in vitro	caspase-9 and caspase-3,	
Wang, Y.	2014	24820432	0	The combinatory effects of PPAR-gamma agonist and survivin inhibition on the cancer stem-like phenotype and cell proliferation in bladder cancer cells	PPAR-gamma	

Table S1. The list of bladder cancer related articles either used or not used RT4.

Author	Year	PMID	using RT4 or not(0 represents No;1 represents Yes)	Title	Gene	Pathway
Zheng, J.	2014	24583128	0	CXCL5 knockdown expression inhibits human bladder cancer T24 cells proliferation and migration	CXCL5	PI3K-AKT and ERK1/2
Kerr, M.	2014	25224279	0	Deoxycytidine kinase expression underpins response to gemcitabine in bladder cancer	dCK	
Hattori, S.	2014	24726313	0	Detection of bladder cancer by measuring CD44v6 expression in urine with real-time quantitative reverse transcription polymerase chain reaction	CD44v6,	
Zhu, Y.	2014	24737586	0	DNA damage-inducible gene, UNC5A, functions as a tumor-suppressor in bladder cancer	p53/UNC5A/	
Lv, L.	2014	25188512	0	The DNA methylation-regulated miR-193a-3p dictates the multi-chemoresistance of bladder cancer via repression of SRSF2/PLAU/HIC2 expression	SRSF2, PLAU and HIC2,miR-193a-3p	miR-193a-3p/SRSF2,PLAU,HIC2/five
Lin, J.	2014	24846322	1	Downregulation of HIPK2 increases resistance of bladder cancer cell to cisplatin by regulating Wip1	HIPK2	HIPK2/Wip1
Fan, Y.	2014	24952510	0	Down-regulation of miR-29c in human bladder cancer and the inhibition of proliferation in T24 cell via PI3K-AKT pathway	miR-29c i	PI3K-AKT
Kyou Kwon, J.	2014	24239466	0	Dual inhibition by S6K1 and Elf4E is essential for controlling cellular growth and invasion in bladder cancer	S6K1 and Elf4E	mTORC1
Ma, L.	2014	24718854	0	Dual targeting of heat shock proteins 90 and 70 promotes cell death and enhances the anticancer effect of chemotherapeutic agents in bladder cancer	HSP90/17-AAG	Akt
Janaszek-Seydli	2014	25618112	0	Effect of different Bacillus Calmette-Guerin substrains on growth inhibition of T24 bladder cancer cells and cytokines secretion by BCG activated peripheral blood mononuclear cells of PBMCs	IFN-gamma, TNF-alpha, IL-12 IL-10, IL-4	
Guo, C.	2014	24393848	0	The effect of Pokemon on bladder cancer epithelial-mesenchymal transition	b-catenin, E-cadherin and Pokemon	
Wang, H. F.	2014	25501205	0	Effect of siRNA targeting EZH2 on cell viability and apoptosis of bladder cancer T24 cells	EZH2	
Wang, Y. H.	2014	24606459	0	Effect of TLR4 and B7-H1 on immune escape of urothelial bladder cancer and its clinical significance	TLR4 and B7-H1	ERK
Zhang, B.	2014	24519067	0	The effects of STAT3 and Survivin silencing on the growth of human bladder carcinoma cells	STAT3 and Survivin	
Spilka, R.	2014	25070653	0	eIF3a is over-expressed in urinary bladder cancer and influences its phenotype independent of translation initiation	eIF3a /RRM2, tyrosinated α-tubulin	
Bu, Q	2014	25109742	0	Enforced expression of miR-101 enhances cisplatin sensitivity in human bladder cancer cells by modulating the cyclooxygenase-2 pathway	miR101 and cyclooxygenase2 (COX2	COX2
Antony, P.	2014	25465919	1	Epigenetic inactivation of ST6GAL1 in human bladder cancer	ST6GAL1,	
Varol, N.	2014	24665856	0	The epigenetically regulated effects of Wnt antagonists on the expression of genes in the apoptosis pathway in human bladder cancer cell line (T24)	CTNNB1, GSK3beta, c-MYC, CCND1, CASP-3, CASP-8, CASP-9, BCL2L1, and WIF-1	

Table S1. The list of bladder cancer related articles either used or not used RT4.

Author	Year	PMID	using RT4 or not(0 represents No;1 represents Yes)	Title	Gene	Pathway
Park, S. L.	2014	25175278	0	EPO gene expression induces the proliferation, migration and invasion of bladder cancer cells through the p21WAF1mediated ERK1/2/NF-kappaB/MMP-9 pathway	EPO /ERK1/2	p21WAF1 ERK1/2/NF-kappaB/MMP-9
Han, B.	2014	24616912	1	Estrogen receptor beta (ERbeta) is a novel prognostic marker of recurrence survival in non-muscle-invasive bladder cancer potentially by inhibiting cadherin switch	Erbeta	E-cadherin and N-cadherin
Srivastava, A. K	2014	24852426	0	Evaluation of urinary XIAP as a diagnostic biomarker of carcinoma of urinary bladder	XIAP	
Zhang, T.	2014	24566141	0	Evodiamine induces apoptosis and enhances TRAIL-induced apoptosis in human bladder cancer cells through mTOR/S6K1-mediated downregulation of Mcl-1	Mcl-1	mTOR/S6K1
Takagi, S.	2014	24222607	0	Expression of Aggrus/podoplanin in bladder cancer and its role in pulmonary metastasis	Aggrus/podoplanin	
Rubenwolf, P. (	2014	24022233	1	Expression of aquaporin water channels in human urothelial carcinoma: correlation of AQP3 expression with tumour grade and stage	AQP3, 4 and 7	aquaporin water channels
Ding, X.	2014	24571540	0	Expression of HMGA2 in bladder cancer and its association with epithelial-to-mesenchymal transition	HMGA2	
Wang, X.	2014	25374080	1	Expression of IkappaBalpha in bladder cancer cell lines is negatively correlated with epithelial-mesenchymal transition and cell invasion in vitro]	IkappaBalpha	E-cadherin and N-cadherin
Acquaviva, J.	2014	24784839	1	FGFR3 translocations in bladder cancer: differential sensitivity to HSP90 inhibition based on drug metabolism	FGFR3TACC3, phosphorylated FGFR3, ERK and AKT (p-FGFR, p-ERK, p-AKT,p-ERK, HER2, CDC2, and BIM	
Xiao, W.	2014	25234557	0	Fibulin-1 is epigenetically down-regulated and related with bladder cancer recurrence	Fibulin-1	
Park, H. Y.	2014	24818577	0	Fucoidan inhibits the proliferation of human urinary bladder cancer T24 cells by blocking cell cycle progression and inducing apoptosis	p21 to Cdk4/6,pRB,E2Fs	caspase-9/-3
Wang, L.	2014	25187415	0	Fucoxanthin induces growth arrest and apoptosis in human bladder cancer T24 cells by up-regulation of p21 and down-regulation of mortalin	CDK-2, CDK-4, cyclin D1, and cyclin E.	
Pereira, P. M.	2014	24763311	0	Galactodendritic phthalocyanine targets carbohydrate-binding proteins enhancing photodynamic therapy	GLUT1 and galectin-1	
Ishaq, M.	2014	25218692	0	Gambogic acid induced oxidative stress dependent caspase activation regulates both apoptosis and autophagy by targeting various key molecules (NF-kappaB, Beclin-1, p62 and NBR1) in human bladder cancer cells	p62 and NBR1	
Pinto-Leite, R.	2014	24459064	0	Genomic characterization of three urinary bladder cancer cell lines: understanding genomic types of urinary bladder cancer	HRAS, BCL2L1 and PTPN1	

Table S1. The list of bladder cancer related articles either used or not used RT4.

Author	Year	PMID	using RT4 or not(0 represents No;1 represents Yes)	Title	Gene	Pathway
Lee, C.	2014	25045449	0	Growth inhibition after exposure to transforming growth factor-beta1 in human bladder cancer cell lines	TGF-beta1	
Gao, L.	2014	25356172	0	GSTP1 arrests bladder cancer T24 cells in G0/G1 phase and up-regulates p21 expression	GSTP1	p21.
Guan, Z.	2014	24316875	0	HAF drives the switch of HIF-1alpha to HIF-2alpha by activating the NF-kappaB pathway, leading to malignant behavior of T24 bladder cancer cells	HAF/ HIF-1alpha to HIF-2alpha	
Lu G et al.	2014	25001935	0	Vimentin gene transfection promotes the invasion of bladder cancer SW780 cells	MMP9	
Schempp CM et	2014	24482380	0	V-ATPase inhibition regulates anoikis resistance and metastasis of cancer cells	BIM、ERK、AKT	ERK、PI3K-AKT
Min Feng Chen	2014	25017509	0	Transforming growth factorbeta1 induces epithelialmesenchymal transition and increased expression of matrix metalloproteinase16 via miR200b downregulation in bladder cancer cells	TGFB1、MMP16、MIR200b	
Wu CT et al.	2014	24886404	0	Thrombomodulin expression regulates tumorigenesis in bladder cancer	TM、MMP9、ECA、VEGF、CD31 、Snail、bcl-2、p53、bax、Caspase3 、cleavaged、DNMT1	NF-κB、EMT
Huang YT et al.	2014	24297644	0	Therapeutic potential of sepantronium bromide YM155 in gemcitabine-resistant human urothelial carcinoma cells	cyclin A、cyclin B、cyclin D1、cyclin E、CDK2、CDK4、CDK6、p21、ki-67、bcl-2、	apoptotic、autophagic
Xu R et al.	2014	25142698	0	TAT-RhoGDI2, a novel tumor metastasis suppressor fusion protein: expression, purification and functional evaluation	RhoGDI2	
Wang W et al.	2014	25387670	0	Targeting PPM1D by lentivirus-mediated RNA interference inhibits the tumorigenicity of bladder cancer cells	PPM1D	
Zhang SN et al.	2014	26027129	0	Synergism inhibition of curcumin combined with cisplatin on T24 bladder carcinoma cells and its related mechanism	GSTP1、NQOl	
Hsu I et al.	2014	24148819	0	Suppression of ERbeta signaling via ERbeta knockout or antagonist protects against bladder cancer development	Erβ、MCM5、E2F1、	ERβ
Liao H et al.	2014	26629935	0	Suppression of Cellular Proliferation and Invasion by HMGB1 Knockdown in Bladder Urothelial Carcinoma Cells	HMGB1	
Park HS et al.	2014	24296129	0	Sulforaphane induces reactive oxygen species-mediated mitotic arrest and subsequent apoptosis in human bladder cancer 5637 cells	cyclin B1 、Cdk1、caspase-8 、caspase-9	ROS pathway
Jo GH et al.	2014	24993616	0	Sulforaphane induces apoptosis in T24 human urinary bladder cancer cells through a reactive oxygen species-mediated mitochondrial pathway: the involvement of endoplasmic reticulum stress and the Nrf2 signaling pathway	caspase-9、caspase-3、 Bcl-2、Bax、	Nrf2
Foulks JM et al.	2014	24953177	1	A small-molecule inhibitor of PIM kinases as a potential treatment for urothelial carcinomas	PIM 、PL3K、BAD、	
Guo S et al.	2014	24752098	0	SCF/c-Kit signaling promotes invasion of T24 cells via PI3K pathway	SCF/c-Kit	PI3K、SCF/c-Kit

Table S1. The list of bladder cancer related articles either used or not used RT4.

Author	Year	PMID	using RT4 or not(0 represents No;1 represents Yes)	Title	Gene	Pathway
Aparicio LA et a	2014	25012153	0	Role of the microtubule-targeting drug vinflunine on cell-cell adhesions in bladder epithelial tumour cells	VFL、E-cadherin 、N-cadherin 、cyclin D1	
Zhou C et al.	2014	24535223	0	Resveratrol induces apoptosis of bladder cancer cells via miR21 regulation of the Akt/Bcl2 signaling pathway	AKT、Bcl-2、miR-21	Akt/Bcl2
Sobolesky PM e	2014	25202904	0	Regulation of the tumor suppressor FOXO3 by the thromboxane-A2 receptors in urothelial cancer	FOXO3、ERK、p27、SIRT1、TPβ	
Chang L et al.	2014	23948182	0	Pseudomonas aeruginosa-mannose-sensitive hemagglutinin inhibits epidermal growth factor receptor signaling pathway activation and induces apoptosis in bladder cancer cells in vitro and in vivo	EGFR、AKT、ERK、caspase-9、caspase-3、caspase-8	
Jiang L et al.	2014	24904997	0	Proteomic analysis of bladder cancer indicates Prx-I as a key molecule in BI-TK/GCV treatment system	Prx-I	NF-κB
Hemdan T et al.	2014	25072257	0	The prognostic value and therapeutic target role of stathmin-1 in urinary bladder cancer	STMN1	
Braig S et al.	2014	24434509	0	Pretubulysin: a new option for the treatment of metastatic cancer	Mcl-1 、 Bcl-xL、JNK、E3、P43、BID	
Yan S et al.	2014	24853624	0	The PPARgamma agonist Troglitazone induces autophagy, apoptosis and necroptosis in bladder cancer cells	PARP、P62、LC-3、ERK	AMPK
Cirone P et al.	2014	24442130	0	Patient-derived xenografts reveal limits to PI3K/mTOR- and MEK-mediated inhibition of bladder cancer	AKT、ERK、STATA3、	PI3K/mTOR
Giacoia EG et a	2014	24464915	1	PAI-1 leads to G1-phase cell-cycle progression through cyclin D3/cdk4/6 upregulation	CDK4、cyclin D3、CDK8、cyclin E、CDK2、P21、P27	
Park SL et al.	2014	24504262	0	p27KIP1 is involved in ERK1/2-mediated MMP-9 expression via the activation of NF-kappaB binding in the IL-7-induced migration and invasion of 5637 cells	IL-7、ERK1/2、 MMP-9	
Park SL et al.	2014	24953855	0	p21WAF1 mediates the IL-15-induced migration and invasion of human bladder cancer 5637 cells via the ERK1/2/NF-kappaB/MMP-9 pathway	IL-15、MMP9、MMP2、	ERK1/2/NF-κB/MMP-9
Ye P et al.	2014	25002527	0	Nrf2- and ATF4-dependent upregulation of xCT modulates the sensitivity of T24 bladder carcinoma cells to proteasome inhibition	Nrf2、ATF4	
Ai X et al.	2014	25109409	0	Notch-1 regulates proliferation and differentiation of human bladder cancer cell lines by inhibiting expression of Kruppel-like factor 4	KLF4、Notch-1 、	
Fang Y et al.	2014	25121353	0	A new tumour suppression mechanism by p27Kip1: EGFR down-regulation mediated by JNK/c-Jun pathway inhibition	p27、EGFR、JNK、SP-1、ATF-2、NF-AT3、COX-2、	JNK/c-Jun
Du HF et al.	2014	24316392	0	A new PKCalpha/beta/TBX3/E-cadherin pathway is involved in PLCepsilon-regulated invasion and migration in human bladder cancer cells	PKCα/β、TBX3、E-cadherin	PKCalpha/beta/TBX3/E-cadherin

Table S1. The list of bladder cancer related articles either used or not used RT4.

Author	Year	PMID	using RT4 or not(0 represents No;1 represents Yes)	Title	Gene	Pathway
Yang J et al.	2014	24262005	0	N1-guanyl-1,7-diaminoheptane sensitizes bladder cancer cells to doxorubicin by preventing epithelial-mesenchymal transition through inhibition of eukaryotic translation initiation factor 5A2 activation	F5A2、E-cadherin、vimentin、Twist-1	
Knievel J et al.	2014	25387078	0	Multiple mechanisms mediate resistance to sorafenib in urothelial cancer	AKT、PI3K、Bcl-2、Mcl-1、VEGFR1 / -R2	MAPK
da Silva GN et a	2014	24652204	1	MRE11A and SKP2 genes are associated with the increased cytotoxicity induced by the synergistic effects of cisplatin and gemcitabine in bladder cancer cells	MRE11A、SKP2	
Zeng T et al.	2014	25550801	0	miR-451 inhibits invasion and proliferation of bladder cancer by regulating EMT	miR-451、E-cadherin、N-cadherin、snail、vimentin	
Chao Shang et a	2014	24443232	0	MiR-320a down-regulation mediates bladder carcinoma invasion by targeting ITGB3	miR-320a、ITGB3、	
Lei Liu et al.	2014	25367080	0	miR-200c inhibits invasion, migration and proliferation of bladder cancer cells through down-regulation of BMI-1 and E2F3	miR-200c、BMI-1 、E2F3、E-cadherin、N-cadherin、Vimentin、P14、P16,	
Hui Deng et al.	2014	25311867	0	miR-193a-3p regulates the multi-drug resistance of bladder cancer by targeting the LOXL4 gene and the oxidative stress pathway	miR-193a-3p、LOXL4、	Oxidative Stress pathway
Ye Lei et al.	2014	25287716	0	miR-150 modulates cisplatin chemosensitivity and invasiveness of muscle-invasive bladder cancer cells via targeting PDCD4 in vitro	miR-150、PDCD4、	
Kou B et al.	2014	24954107	0	miR-145 inhibits invasion of bladder cancer cells by targeting PAK1	miR-145、PAK1	
Xiao-nan Chen	2014	25414595	0	MiR-133b regulates bladder cancer cell proliferation and apoptosis by targeting Bcl-w and Akt1	MiR-133b、Bcl-w、 Akt1、	
Itesako T et al.	2014	24520312	0	The microRNA expression signature of bladder cancer by deep sequencing: the functional significance of the miR-195/497 cluster	miR - 195、miR - 497	
Wang X et al.	2014	25178497	0	MicroRNA-320c inhibits tumorous behaviors of bladder cancer by targeting Cyclin-dependent kinase 6	miR - 320c、CDK6	
Wu D et al.	2014	24944696	0	microRNA-99a inhibiting cell proliferation, migration and invasion by targeting fibroblast growth factor receptor 3 in bladder cancer	miRNA- 99a、FGFR3	
Yu G et al.	2014	25551284	0	MicroRNA-34a functions as an anti-metastatic microRNA and suppresses angiogenesis in bladder cancer by directly targeting CD44	mir- 34a、CD44、	
Zhang T et al.	2014	24714080	1	Metformin sensitizes human bladder cancer cells to TRAIL-induced apoptosis through mTOR/S6K1-mediated downregulation of c-FLIP	c - FLIP、mTOR 、S6K1	mTOR / S6K1
Pacini L et al.	2014	25482946	0	M2muscarinic receptors inhibit cell proliferation and migration in urothelial bladder cancer cells	M2	
Li Y et al.	2014	24448324	0	Loss of GATA3 in bladder cancer promotes cell migration and invasion	GATA3、MMP-2、MMP-9、N-cadherin、	

Table S1. The list of bladder cancer related articles either used or not used RT4.

Author	Year	PMID	using RT4 or not(0 represents No;1 represents Yes)	Title	Gene	Pathway
Wang X et al.	2014	24993775	0	Long non-coding RNA urothelial carcinoma associated 1 induces cell replication by inhibiting BRG1 in 5637 cells	BRG1、UCA1、p21、	
Fan Y et al.	2014	24495014	0	Long non-coding RNA UCA1 increases chemoresistance of bladder cancer cells by regulating Wnt signaling	UCA1、WNT6	Wnt signaling
Yuan X et al.	2014	24384411	0	Licochalcone B inhibits growth of bladder cancer cells by arresting cell cycle progression and inducing apoptosis	Cdc25A、 Cdc25B、CDK1、 CDK2 、Bcl-2、bax、PARP、	
Yen HK et al.	2014	25107315	1	Involvement of Seladin-1 in goniothalamin-induced apoptosis in urinary bladder cancer cells	Seladin-1	
Morais DR et al.	2014	25493074	0	The involvement of miR-100 in bladder urothelial carcinogenesis changing the expression levels of mRNA and proteins of genes related to cell proliferation, survival, apoptosis and chromosomal stability	miR - 100、FGFR3、BAZ2A、mTOR 、SMARCA5、	
Zhu J et al.	2014	24606737	0	Inhibitory effect of 2'-hydroxyflavanone on proliferation, invasion and migration of bladder cancer cells in vitro via blocking AKT/STAT3 signaling pathway	MMP- 2、MMP-9、AKT、STAT3	AKT/STAT3 signaling pathway
Gai JW et al.	2014	23628311	0	Inhibition of presenilins attenuates proliferation and invasion in bladder cancer cells through multiple pathways	PS1、PS2、CD44、VEGFR-1、VEGFR-2、PCNA、cyclin D1、cyclin B1、Bcl-2、ERK、BAD、MMP2、MMP7、MMP9	
Ojha R et al.	2014	24440234	0	Inhibition of grade dependent autophagy in urothelial carcinoma increases cell death under nutritional limiting condition and potentiates the cytotoxicity of chemotherapeutic agent	LC3、atg7、caspase-9、AMPK、mTOR、	
Kang M et al.	2014	24815071	0	Inhibition of autophagy potentiates atorvastatin-induced apoptotic cell death in human bladder cancer cells in vitro	SQSTM1、LC3、PARP、p62、	
Hamm R et al.	2014	24976507	0	Induction of cholesterol biosynthesis by archazolid B in T24 bladder cancer cells	SREBP、HMGCR、LDLR、	
Wei D et al.	2014	25022381	0	HMGN2 protein inhibits the growth of infected T24 cells in vitro	HMGN2	
Zhu, Z. et al.	2013	23403865	0	Short hairpin RNA targeting FOXQ1 inhibits invasion and metastasis via the reversal of epithelial-mesenchymal transition in bladder cancer	FOXQ1、TGF-β1、E-cadherin、Vimentin	EMT
Zhu, Y. P. et al.	2013	23599794	0	Pseudomonas aeruginosa-mannose-sensitive hemagglutinin inhibits proliferation and induces apoptosis in a caspase-dependent manner in human bladder cancer cell lines	Caspase8、Caspase9、Fas、AKT、mTOR、ERK	PI3K-AKT-mTOR
Zhu, Y. et al.	2013	23724790	0	Apigenin promotes apoptosis, inhibits invasion and induces cell cycle arrest of T24 human bladder cancer cells	caspase-3、AKT、PDK、PI3K、Bax 、PARP、Bad、Bcl-xl、Bcl-2	PI3K/Akt
Zhu, H. B. et al.	2013	23356234	0	Silencing of mutant p53 by siRNA induces cell cycle arrest and apoptosis in human bladder cancer cells	Cyclin A、Cyclin E、Cyclin D1、Cyclin B1、CDK1、CDK2、CDK4、Caspase9、Caspase3、PARP	Mutant p53
Zhou, Y.et al.	2013	23206218	0	MicroRNA-133 inhibits cell proliferation, migration and invasion by targeting epidermal growth factor receptor and its downstream effector proteins in bladder cancer	miR-133a、miR-133b	EGFR

Table S1. The list of bladder cancer related articles either used or not used RT4.

Author	Year	PMID	using RT4 or not(0 represents No;1 represents Yes)	Title	Gene	Pathway
Zhou, N.et al.	2013	23403633	1	The investigational Aurora kinase A inhibitor MLN8237 induces defects in cell viability and cell-cycle progression in malignant bladder cancer cells in vitro and in vivo	PARP、P53、P21、P73、Ki-67、TUNEL	
Zheng, Q. Y.et a	2013	23000344	0	Ursolic acid induces ER stress response to activate ASK1-JNK signaling and induce apoptosis in human bladder cancer T24 cells	ER、PERK、eIF2α、CHOP、Bim、ASK1、JNK、AMPK、ACC、TRAF2、IRE-1	ASK1–JNK
Zhao, Q.et al.	2013	23407876	0	Expression of a tumor-associated gene, LASS2, in the human bladder carcinoma cell lines BIU-87, T24, EJ and EJ-M3	LASS2、LASS1、LASS3	
Zhang, Z. et al.	2013	22192978	0	High expression of polo-like kinase 1 is associated with the metastasis and recurrence in urothelial carcinoma of bladder	Plk1	regulating the cancer cell cycle from G1/S to G2/M
Zhang, Y.et al.	2013	23484808	0	Knockdown of phospholipase C-epsilon by short-hairpin RNA-mediated gene silencing induces apoptosis in human bladder cancer cell lines	PLCe、bcl-2、bax	bcl-2、bax
Zhang, T.et al.	2013	24351837	0	The antidiabetic drug metformin inhibits the proliferation of bladder cancer cells in vitro and in vivo	cyclin D1、CDK4、E2F1、p21、AMPK、mTOR、PCNA	AMPK-mTOR
Zhang, N.et al.	2013	23645739	0	Expression of bone morphogenetic protein-10 (BMP10) in human urothelial cancer of the bladder and its effects on the aggressiveness of bladder cancer cells in vitro	BMP10、ALK1-7、BMPR2、ACTR2A、ACTR2B	
Zhang, J.et al.	2013	23722651	0	Overexpression of Rab25 contributes to metastasis of bladder cancer through induction of epithelial-mesenchymal transition and activation of Akt/GSK-3beta/Snail signaling	Rab25、N-cadherin、E-cadherin、a-catenin、Vimentin、Fibronectin、Akt、GSK-3β、Snail	Akt/GSK-3β/Snail、EMT
Zhang, J.et al.	2013	23869380	0	Single molecular dissection of the ligand binding property of epidermal growth factor receptor	EGF、TGF-α、EGFR	
Zhang, H. et al.	2013	23570371	1	5-Aza-2'-deoxycytidine enhances maspin expression and inhibits proliferation, migration, and invasion of the bladder cancer T24 cell line	Maspin、Cyclin D1，VEGF-C，VEGFR-3，MMP-2、MMP-9、caspase-3、p17、Bax、Bcl-2	Bcl-2 / Bax
Yuan, X. et al.	2013	23936805	0	Licochalcone A-induced human bladder cancer T24 cells apoptosis triggered by mitochondria dysfunction and endoplasmic reticulum stress	Bcl-2、Bax、Caspase-9、Caspase-3、PARP、GRP78、CHOP、Caspase-12	mitochondria-dependent and the ER stress-triggered signaling pathways
Yoshii, H.et al.	2013	23817592	0	Increased expression of alpha-actinin-4 is associated with unfavorable pathological features and invasiveness of bladder cancer	ACTN4、AKT、STAT3、ERK	ERK
Ying, H.et al.	2013	23612802	0	TLR4 mediates MAPK-STAT3 axis activation in bladder epithelial cells	TLR4、ERK、p38、JNK、STAT3、IL6、IL10、	MAPK–STAT3
Yao, X. et al.	2013	23703635	0	A novel role of ribonuclease inhibitor in regulation of epithelial-to-mesenchymal transition and ILK signaling pathway in bladder cancer cells	RI、AKT、GSK3、N-cadherin、E-cadherin、Vimentin、MMP2、Slug、Twist、Cyclin D1、Snail、ILK、MMP9	EMT、ILK signaling
Yang, X. et al.	2013	23591341	0	Cisplatin enhances apoptosis in bladder cancer cells via autophagy	LC3-I、LC3-II、PARP、mTOR、P70S6K	

Table S1. The list of bladder cancer related articles either used or not used RT4.

Author	Year	PMID	using RT4 or not(0 represents No;1 represents Yes)	Title	Gene	Pathway
Yang, L. et al.	2013	23969721	0	Bladder cancer cell-derived exosomes inhibit tumor cell apoptosis and induce cell proliferation in vitro	Bcl-2、Cyclin D1、Bax、caspase-3、Akt、ERK、TK1、XIAP、CD60、survivin	Akt、ERK
Yang, D. R.et al	2013	23522297	0	Higher expression of peroxisome proliferator-activated receptor gamma or its activation by agonist thiazolidinedione-rosiglitazone promotes bladder cancer cell migration and invasion	PPARγ	
Yan, L.et al.	2013	23065570	0	Berberine inhibits the migration and invasion of T24 bladder cancer cells via reducing the expression of heparanase	HPA	
Yan, L.et al.	2013	23873098	1	Target protein for Xklp2 (TPX2), a microtubule-related protein, contributes to malignant phenotype in bladder carcinoma	TPX2、cyclin D1 、 cdk2 、p21 、caspase-3	
Xue, Y.et al.	2013	23275123	0	CIP2A is a predictor of survival and a novel therapeutic target in bladder urothelial cell carcinoma	CIP2A、c-MYC	
Xu, X.et al.	2013	24180482	0	MicroRNA-124-3p inhibits cell migration and invasion in bladder cancer cells by targeting ROCK1	MicroRNA-124-3p、ROCK1，MMP2、MMP9、c-Met、P38、N-cadherin、E-cadherin、Vimentin、Fibronectin	ROCK1
Xu, F.et al.	2013	24265332	0	Effect of miR-29b-1* and miR-29c knockdown on cell growth of the bladder cancer cell line T24	miR-29b-1、miR-29c	
Wu, S. et al.	2013	23593475	0	Microvesicles derived from human umbilical cord Wharton's jelly mesenchymal stem cells attenuate bladder tumor cell growth in vitro and in vivo	Akt、p53、p21、Caspase 3	Akt
Wu, K.et al.	2013	22421353	1	Slug contributes to cadherin switch and malignant progression in muscle-invasive bladder cancer development	Snail、Slug、N-cadherin、E-cadherin、Vimentin、β-catenin、ZEB1、ZEB2、Twist1	EMT
Wu, K. et al.	2013	24012496	1	Silibinin inhibits beta-catenin/ZEB1 signaling and suppresses bladder cancer metastasis via dual-blocking epithelial-mesenchymal transition and stemness	ZEB1、MMP2、CD44、cytokeratin18, cytokeratin19, vimentin、MMP2、GSK3β	EMT
Wu, J. Y.et al.	2013	23573134	0	Anti-Bladder-Tumor Effect of Baicalein from Scutellaria baicalensis Georgi and Its Application In Vivo	Cyclin B1、Cyclin D1、GSK3β、Akt、ERK、p38、	Akt
Wu, J.et al.	2013	24299210	0	Nur77 inhibits androgen-induced bladder cancer growth	Nur77、src-1	
Wu, D.et al.	2013	23425975	0	microRNA-125b inhibits cell migration and invasion by targeting matrix metallopeptidase 13 in bladder cancer	microRNA-125b、MMP13	
Wongpaiboonw	2013	23886181	0	Oxidative stress induces hypomethylation of LINE-1 and hypermethylation of the RUNX3 promoter in a bladder cancer cell line	LINE-1、IL-1、RUNX3	
Wei, J.et al.	2013	23127333	0	The inhibition of human bladder cancer growth by calcium carbonate/CaIP6 nanocomposite particles delivering AIB1 siRNA	AIB1、PI3K、 Akt	PI3K/Akt
Wang, W.et al.	2013	23720881	0	Expression and role of miR-34a in bladder cancer	miR-34a、caspase-3	
Wang, H.et al.	2013	23376440	0	Sodium arsenite induces cyclooxygenase-2 expression in human uroepithelial cells through MAPK pathway activation and reactive oxygen species induction	COX-2、MAPK、GSH、Nrf2、JNK、ERK、p38、	MAPK
Wang, H.et al.	2013	24119899	0	Double-mutated oncolytic adenovirus combined with gemcitabine for treating an orthotopic nude mouse model of bladder cancer	CAR	

Table S1. The list of bladder cancer related articles either used or not used RT4.

Author	Year	PMID	using RT4 or not(0 represents No;1 represents Yes)	Title	Gene	Pathway
Wang, H.et al.	2013	23575075	0	M-ds-P21 induces cell apoptosis in bladder cancer T24 cells through P53 independent pathway	P53、P21、	
Wang, H.et al.	2013	23895116	0	Compound K induces apoptosis of bladder cancer T24 cells via reactive oxygen species-mediated p38 MAPK pathway	caspases-3、caspases-9、Bax、Bcl-2、p38、cytochrome c	p38 MAPK
Wang, H.et al.	2013	23102779	0	Antitumor effects of exogenous ganglioside GM3 on bladder cancer in an orthotopic cancer model	EGF-R	
Wang, F.et al.	2013	23968725	0	Arsenic induces the expressions of angiogenesis-related factors through PI3K and MAPK pathways in SV-HUC-1 human uroepithelial cells	COX-2、VEGF、HIF-1、PI3K/AKT、MAPK、ERK1/2、p38、JNK	PI3K、MAPK
Wang, C.et al.	2013	23430583	0	Apoptin induces apoptosis in nude mice allograft model of human bladder cancer by altering multiple bladder tumor-associated gene expression profiles	caspase-3、Ki67、C-erbB-2、 Rb、nm23	
Vasconcelos-Nc	2013	22169072	0	In vivo and in vitro effects of RAD001 on bladder cancer	Akt、mTOR	
Uchino, K.et al.	2013	23295946	1	Therapeutic effects of microRNA-582-5p and -3p on the inhibition of bladder cancer progression	MicroRNA-582-5p、MicroRNA-582-3p、PGGT1B、LRRK2、DIXDC1、KCNC1、RAB27A	
Tsui, K. H.et al.	2013	23762858	1	Mechanisms by which interleukin-6 attenuates cell invasion and tumorigenesis in human bladder carcinoma cells	IL6、NDRG1、KAI1、MASPIN、N-cadherin、E-cadherin、Vimentin	EMT
Telu, K. H. et al	2013	23675690	1	Alterations of histone H1 phosphorylation during bladder carcinogenesis	T146	
Tao, J.et al.	2013	24324362	0	Identification of hypermethylation in hepatocyte cell adhesion molecule gene promoter region in bladder carcinoma	hepaCAM	
Tang, K.et al.	2013	23495258	0	The role of phenethyl isothiocyanate on bladder cancer ADM resistance reversal and its molecular mechanism	Top-II、MDR1、MRP1、bcl-2、GST-π、NF-kB、Survivin、Twist、PTEN、Akt、JNK	MAPK
Tan, M. Y.et al.	2013	24008762	0	SUMO-specific protease 2 suppresses cell migration and invasion through inhibiting the expression of MMP13 in bladder cancer cells	SENP2、NF-κB、MMP13	
Tan, J.et al.	2013	24255584	0	Apoptosis of bladder transitional cell carcinoma T24 cells induced by adenovirus-mediated inducible nitric oxide synthase gene transfection	iNOS、p53、	
Sun, Y.et al.	2013	22920439	1	Inhibition of STAT signalling in bladder cancer by diindolylmethane: relevance to cell adhesion, migration and proliferation	STAT1、STAT3、STAT5、lamin、LDH、PARP、cyclin D1、survivin、cdk6、cdc25C、cdk1/cdc2、cyclin B1、Plk1、ERK、AKT	STAT
Su, G.et al.	2013	23439133	1	The effect of forced growth of cells into 3D spheres using low attachment surfaces on the acquisition of stemness properties	NANOG 、SOX2、	
Song, T.et al.	2013	23568547	0	miR-708 promotes the development of bladder carcinoma via direct repression of Caspase-2	miR-708、Caspase-2	
Shin, D. Y.et al.	2013	24084732	0	Inhibiting invasion into human bladder carcinoma 5637 cells with diallyl trisulfide by inhibiting matrix metalloproteinase activities and tightening tight junctions	MMP-2、MMP-9、TIMP-1、TIMP-2、Claudin-1、Claudin-2、Claudin-3、Claudin-4、Claudin-5	

Table S1. The list of bladder cancer related articles either used or not used RT4.

Author	Year	PMID	using RT4 or not(0 represents No;1 represents Yes)	Title	Gene	Pathway
Shimizu, T.et al	2013	23200812	0	Methylation of a panel of microRNA genes is a novel biomarker for detection of bladder cancer	miR-137、miR-124-2、miR-124-3、miR-9-3	
Shen, H. B.et al.	2013	23526079	0	CXCR4-mediated Stat3 activation is essential for CXCL12-induced cell invasion in bladder cancer	CXCL12、CXCR4、Stat3	CXCL12 / CXCR4
Shan, Y.et al.	2013	23159064	0	Epithelial-mesenchymal transition, a novel target of sulforaphane via COX-2/MMP2, 9/Snail, ZEB1 and miR-200c/ZEB1 pathways in human bladder cancer cells	COX-2、MMP-2、MMP-9、Snail、E-cadherin、ZEB1	COX-2/MMP2,9/Snail, ZEB1 and miR-200c/ZEB1
Sgnaolin, V.et a	2013	23224295	1	Functional and molecular characterization of kinin B1 and B 2 receptors in human bladder cancer: implication of the PI3Kgamma pathway	p38、MAP， PI3Kg ,AKT,ERK 1/2	PI3Kg ,AKT,ERK 1/2
Secanella-Fandc	2013	22982433	1	Connaught and Russian strains showed the highest direct antitumor effects of different Bacillus Calmette-Guerin substrains	IL6、IL8	
Santoni, M.et al	2013	23887605	0	Pazopanib and sunitinib trigger autophagic and non-autophagic death of bladder tumour cells	LC3-I、LC3-II、p62、ERK、	
Ruan, J.et al.	2013	23307254	0	Predictive value of Sox2 expression in transurethral resection specimens in patients with T1 bladder cancer	Sox2、Ki-67	
Ren, M. H.et al.	2013	23924452	0	Antitumor effects of mutant endostatin are enhanced by Bcl-2 antisense oligonucleotides in UM-UC-3 bladder cancer cell line	Bcl-2、caspase-3	
Rasul, A.et al.	2013	23348995	0	Induction of apoptosis by costunolide in bladder cancer cells is mediated through ROS generation and mitochondrial dysfunction	Bax、Bcl-2、survivin、caspase-3、PARP	Bcl-2 / Bax
Raina, K.et al.	2013	23831192	0	Role of oxidative stress in cytotoxicity of grape seed extract in human bladder cancer cells	LC3-I、LC3-II、caspase-3 、caspase-9、PARP、Mcl-1	
Qiu, Z.et al.	2013	23811531	0	In vitro antioxidant and antiproliferative effects of ellagic acid and its colonic metabolite, urolithins, on human bladder cancer T24 cells	p38-MAPK、MEKK1、c-Jun、Caspase-3、PPAR-γ、p53	p38-MAPK, c-Jun
Qi, Y.et al.	2013	23392718	0	Over-expression of LRIG3 suppresses growth and invasion of bladder cancer cells	LRIG3	
Qi, W.et al.	2013	23874968	0	Inhibition of inducible heat shock protein-70 (hsp72) enhances bortezomib-induced cell death in human bladder cancer cells	Hsp72、HSF1、HSPA1B、HSPA1A、	
Piantino, C. B.et	2013	23644847	1	Prima-1 induces apoptosis in bladder cancer cell lines by activating p53	p53、Bax、Puma、caspase 2、caspase 3、caspase 7、caspase 9、BAK1、MDM2、PMAIP1、B2M、p21、APAF1、CYC1	
Peng, C. C.et al.	2013	23738328	0	Cytotoxicity of ferulic Acid on T24 cell line differentiated by different microenvironments	Bcl-2、Bax，caspase-3、caspase-9	
Pagliarulo, V.et	2013	23705854	0	The interaction of celecoxib with MDR transporters enhances the activity of mitomycin C in a bladder cancer cell line	COX 2、P-gp、MRP1、BCRP	
Noguchi, S. et a	2013	23104321	0	Replacement treatment with microRNA-143 and -145 induces synergistic inhibition of the growth of human bladder cancer cells by regulating PI3K/Akt and MAPK signaling pathways	microRNA-143、 microRNA-145、ERK5、ILK1、Akt、c-MYC、ERK1/2、cyclin D1、 cyclin E、SIRT1、PARP-1、GSK3a/β	PI3K/Akt，MAPK

Table S1. The list of bladder cancer related articles either used or not used RT4.

Author	Year	PMID	using RT4 or not(0 represents No;1 represents Yes)	Title	Gene	Pathway
Noguchi, S.et al	2013	23392170	0	socs7, a target gene of microRNA-145, regulates interferon-beta induction through STAT3 nuclear translocation in bladder cancer cells	microRNA-145、socs7、STAT3、TLR3、PARP-1、caspase 8、JNK、Akt、ERK1/2	Akt
Munk, M.et al.	2013	24015958	1	The HER4 isoform JM-a/CYT2 relates to improved survival in bladder cancer patients but only if the estrogen receptor alpha is not expressed	HER4、ER-α、JM-a、JM-b、CYT1 、CYT2	
Morikawa, T. et	2013	23826418	0	UBE2C is a marker of unfavorable prognosis in bladder cancer after radical cystectomy	UBE2C	
Montagner, I. M	2013	22341413	1	Paclitaxel-hyaluronan hydrosoluble bioconjugate: mechanism of action in human bladder cancer cell lines	CD44、RHAMM	
Monfared, H.et	2013	24078506	0	Co-regulated expression of TGF-beta Variants and miR-21 in bladder cancer	TGF-β、miR-21	
Mo, M.et al.	2013	24137381	0	Roles of mitochondrial transcription factor A and microRNA-590-3p in the development of bladder cancer	microRNA-590-3p、TFAM、PI3K、Akt、MMP2、MMP9	
Marchbank, T.a	2013	23698120	1	Pancreatic secretory trypsin inhibitor causes autocrine-mediated migration and invasion in bladder cancer and phosphorylates the EGF receptor, Akt2 and Akt3, and ERK1 and ERK2	EGF 、Akt2、Akt3、ERK1 、 ERK2	
Malgor, R.et al.	2013	23947922	0	Correlation of Wnt5a expression with histopathological grade/stage in urothelial carcinoma of the bladder	Wnt5a	
Majumdar, S.et	2013	23814487	1	Loss of Sh3gl2/endophilin A1 is a common event in urothelial carcinoma that promotes malignant behavior	Sh3gl2 / Endophilin A1、EGFR、STAT3、SFK	
Lu, X.et al.	2013	23228961	0	beta-elemene inhibits the proliferation of T24 bladder carcinoma cells through upregulation of the expression of Smad4	Smad4	
Lorenzi, T.et al.	2013	23712470	0	HtrA1 in human urothelial bladder cancer: a secreted protein and a potential novel biomarker	HtrA1	
Liu, Z.et al.	2013	23682785	1	The effect of gartanin, a naturally occurring xanthone in mangosteen juice, on the mTOR pathway, autophagy, apoptosis, and the growth of human urinary bladder cancer cell lines	AMPK、mTOR 、AKT、4EBP1、ACC、LC-3II、Bcl-2 、 caspase-3、PARP、p53、PUMA、Bax	mTOR
Liu, X. L.et al.	2013	23835618	0	Dual role of TGFBR3 in bladder cancer	TGFBR3	
Liu, S.et al.	2013	23591579	0	Oxidative stress and MAPK involved into ATF2 expression in immortalized human urothelial cells treated by arsenic	ATF2、JNK、p38	MAPK
Liu, Q.et al.	2013	24223658	1	Effect of TRPV2 cation channels on the proliferation, migration and invasion of 5637 bladder cancer cells	TRPV2、MMP2	
Liu, L.et al.	2013	23677384	0	Decreased expression of miR-430 promotes the development of bladder cancer via the upregulation of CXCR7	miR-430、CXCR7、MMP-2、MMP-9、ERK	
Liu, D.et al.	2013	23325617	0	High FOXM1 expression was associated with bladder carcinogenesis	FOXM1	
Liu, A. G. et al.	2013	23715749	0	RNA interference targeting adrenomedullin induces apoptosis and reduces the growth of human bladder urothelial cell carcinoma	ADM	
Lin, Y. L.et al.	2013	23235385	0	Downregulation of CDH13 expression promotes invasiveness of bladder transitional cell carcinoma	CDH13、MMP2	

Table S1. The list of bladder cancer related articles either used or not used RT4.

Author	Year	PMID	using RT4 or not(0 represents No;1 represents Yes)	Title	Gene	Pathway
Lin, Y.et al.	2013	23796420	0	miR-26a inhibits proliferation and motility in bladder cancer by targeting HMGA1	miR-26a、HMGA1	
Lin, M. Y. et al.	2013	24282433	0	Cortex Moutan Induces Bladder Cancer Cell Death via Apoptosis and Retards Tumor Growth in Mouse Bladders	PARP、caspase-8、caspase-3	
Lin, C.al.	2013	23354080	0	AKT serine/threonine protein kinase modulates baicalin-triggered autophagy in human bladder cancer T24 cells	AKT、Atg5/7/12、Beclin 1、LC-3-I 、LC-3-II	AKT
Liang, W.et al.	2013	23820733	0	Knockdown BMI1 expression inhibits proliferation and invasion in human bladder cancer T24 cells	BMI1、p16、p14、caspase-3、Bcl-2 、Cdk2、Cyclin D1、TWIST、E-cadherin、Vimentin	EMT
Liang, P. Y.al.	2013	23483488	0	Overexpression of immunoglobulin G prompts cell proliferation and inhibits cell apoptosis in human urothelial carcinoma	IgG、caspase-3、PARP	caspase-3
Li, Z.et al.	2013	21872498	0	c-Jun is involved in interstitial cystitis antiproliferative factor (APF)-induced growth inhibition of human bladder cancer T24 cells	c-Jun、APF、p53、p21	
Li, X.et al.	2013	23579952	0	Connexin 26 is down-regulated by KDM5B in the progression of bladder cancer	Cx26、KDM5B、H3K4me1	
Li, W.et al.	2013	24228105	0	High expression of Notch ligand Jagged2 is associated with the metastasis and recurrence in urothelial carcinoma of bladder	Jagged2	Notch
Li, R.et al.	2013	24146910	0	Overexpression of N-Myc downstream-regulated gene 2 (NDRG2) regulates the proliferation and invasion of bladder cancer cells in vitro and in vivo	N-Myc、NDRG2、Cyclin D1、CDK4 、p21	
Li, Q. Q.et al.	2013	23564782	0	beta-Elemene promotes cisplatin-induced cell death in human bladder cancer and other carcinomas	caspase-3/7/8/9/10	caspase-3
Li, H. L.et al.	2013	23064738	0	Baicalein induces apoptosis via a mitochondrial-dependent caspase activation pathway in T24 bladder cancer cells	Akt、Bcl-2、Bax、caspase-9 、caspase-3	Bcl-2 / Bax
Lee, S. T.et al.	2013	24359437	0	Suppression of urinary bladder urothelial carcinoma cell by the ethanol extract of pomegranate fruit through cell cycle arrest and apoptosis	caspase-3/8/9、Bax / Bcl-2、Procaspase-12、CHOP、Bip、DR4、DR5、VCP、Cyclin A	
Lee, S. J.et al.	2013	23271730	0	Interleukin-20 promotes migration of bladder cancer cells through extracellular signal-regulated kinase (ERK)-mediated MMP-9 protein expression leading to nuclear factor (NF-kappaB) activation by inducing the up-regulation of p21(WAF1) protein expression	IL20、ERK、MMP-9、NF-kB、p21	MMP-9/NF-κB
Lee, S.et al.	2013	23313194	0	The role of c-FLIP in cisplatin resistance of human bladder cancer cells	c-FLIP、caspase 2/9、NF-kB、BID、c-FLIP、XIAP、cIAP1/2、Akt	
Lee, E. J.et al.	2013	23770289	0	Interleukin-5 enhances the migration and invasion of bladder cancer cells via ERK1/2-mediated MMP-9/NF-kappaB/AP-1 pathway: involvement of the p21WAF1 expression	IL5、ERK1/2、MMP-9、NF-κB、AP-1、p21、JAK1	MMP-9/NF-κB/AP-1
Kohler, C. U. et	2013	23867826	1	Analyses in human urothelial cells identify methylation of miR-152, miR-200b and miR-10a genes as candidate bladder cancer biomarkers	miR-152、miR-200b 、 miR-10a、	

Table S1. The list of bladder cancer related articles either used or not used RT4.

Author	Year	PMID	using RT4 or not(0 represents No;1 represents Yes)	Title	Gene	Pathway
Kitamura, Y.et a	2013	23791869	0	Chemically modified siRNAs and miRNAs bearing urea/thiourea-bridged aromatic compounds at their 3'-end for RNAi therapy	miR-143	
Kim, J. H.et al.	2013	23819923	1	Human beta-defensin 2 may inhibit internalisation of bacillus Calmette-Guerin (BCG) in bladder cancer cells	HBD-2	
Kim, D. H.et al.	2013	23829315	0	The E2F1 oncogene transcriptionally regulates NELL2 in cancer cells	E2F1、NELL2	
Karkoulis, P. K.	2013	23394616	1	Targeted inhibition of heat shock protein 90 disrupts multiple oncogenic signaling pathways, thus inducing cell cycle arrest and programmed cell death in human urinary bladder cancer cell lines	Hsp90、IGF-IR、c-Met、Akt，IKKα，IKKβ、Erk1 / 2、FOXOs、NF-κB、Rb、Cyclin D1、Cdk4、caspase-3/8/9、PARP、Lamin A/C、Hsp70	
Kanojia, D.et al.	2013	24349057	0	Sperm associated antigen 9 plays an important role in bladder transitional cell carcinoma	SPAG9、p16、p21、CDK4、CDK1、cyclin E、cyclin D、cyclin B	
Kanno, T.et al.	2013	23817168	0	1-[2-(2-Methoxyphenylamino)ethylamino]-3-(naphthalene-1-yloxy)propan-2-ol as a potential anticancer drug	caspase-3/4/8/9	
Kandzari, S. J.et	2013	24917743	0	In vitro regulation of cell growth and angiogenesis by inositol hexaphosphate in bladder cancer	VEGF	
Jones, R. A.et al	2013	22231926	0	The role of TG2 in ECV304-related vasculogenic mimicry	TG2、Collagen I、Collagen IV、Fibronectin、Vinculin、e(c-Glutamyl) lysine crosslink	
Johnen, G.et al.	2013	23691160	1	Cross-contamination of a URotsa stock with T24 cells--molecular comparison of different cell lines and stocks	RARB，PGR，RASSF1，CDH1，FHIT，ESR1、C1QTNF6、PTGS2、SOCS3、MGMT、LINE1	
Jing, P.et al.	2013	23313968	0	DeltaNp63 promotes UMUC3 cell invasiveness and migration through claudin1 in vitro	p63、Claudin-1	
Isono, T.et al.	2013	23560094	0	Study of global transcriptional changes of N-GlcNAc2 proteins-producing T24 bladder carcinoma cells under glucose deprivation	N-GlcNAc2、BiP、RB1	
Hu, Z.et al.	2013	23618864	0	MicroRNA-101 suppresses motility of bladder cancer cells by targeting c-Met	miR-101、c-Met、	c-Met
Hong, S. H.et al	2013	23128598	0	Bufalin prevents the migration and invasion of T24 bladder carcinoma cells through the inactivation of matrix metalloproteinases and modulation of tight junctions	claudin-1/2/3/4、E-cadherin、Snail、MMP2/9、TIMP1/2、ERK	ERK
Hoffman, K. L.e	2013	22965848	1	Raloxifene inhibits growth of RT4 urothelial carcinoma cells via estrogen receptor-dependent induction of apoptosis and inhibition of proliferation	ERα、ERβ、caspase-3、PARP、p27、SKP2、BTG2、cyclin D1	
Han, Y.et al.	2013	23153939	0	Inducing cell proliferation inhibition, apoptosis, and motility reduction by silencing long noncoding ribonucleic acid metastasis-associated lung adenocarcinoma transcript 1 in urothelial carcinoma of the bladder	MALAT1	
Han, Y. et al.	2013	22766726	0	Inducing cell proliferation inhibition and apoptosis via silencing Dicer, Drosha, and Exportin 5 in urothelial carcinoma of the bladder	Dicer、Drosha、Exportin 5	

Table S1. The list of bladder cancer related articles either used or not used RT4.

Author	Year	PMID	using RT4 or not(0 represents No;1 represents Yes)	Title	Gene	Pathway
Han, Y.et al.	2013	22961206	0	Long intergenic non-coding RNA TUG1 is overexpressed in urothelial carcinoma of the bladder	TUG1	
Han, M. H.et al.	2013	23717422	0	Apoptosis induction of human bladder cancer cells by sanguinarine through reactive oxygen species-mediated up-regulation of early growth response gene-1	Bax、Bid、XIAP、caspases-3/8/9、NAC、JNK、Egr-1	
Han, B.et al.	2013	23696028	0	Expression and biological roles of SATB1 in human bladder cancer	SATB1、caspase3、cyclin A、 cyclin D1、cyclin D3、 cyclin E	
Gupta, S.et al.	2013	24312263	1	Mammalian target of rapamycin complex 2 (mTORC2) is a critical determinant of bladder cancer invasion	Akt, Rac1,mTOR	mTOR
Guo, Y.et al.	2013	23401075	1	TSC1 involvement in bladder cancer: diverse effects and therapeutic implications	TSC1,TSC2,Akt,EGFR,ERK1/2	Mtor,Akt
Guo, P.et al.	2013	23242865	0	Preparation of a novel organoselenium compound and its anticancer effects on cervical cancer cell line HeLa	caspase-3, cleaved-PARP, bcl-2	caspase
Grau, L. et al.	2013	23308193	0	A quantitative proteomic analysis uncovers the relevance of CUL3 in bladder cancer aggressiveness	CUL3,GSN,EGFR,NPM,EZR	EGFR
Gotoh, A.et al.	2013	23394881	0	Fiber-substituted conditionally replicating adenovirus Ad5F35 induces oncolysis of human bladder cancer cells in in vitro analysis	CAR,CD46	CAR
Gomes-Giacoia,	2013	24072883	0	Targeting plasminogen activator inhibitor-1 inhibits angiogenesis and tumor growth in a human cancer xenograft model	caspase-3, cleaved-PARP, Fas, and FasL	PARP
Gai, L.et al.	2013	23483134	0	Ursolic acid induces apoptosis via Akt/NF-kappaB signaling suppression in T24 human bladder cancer cells	Akt / NF-κB	Akt / NF-κB
Felipe, K. B.et a	2013	23523795	0	Antiproliferative effects of phenylaminonaphthoquinones are increased by ascorbate and associated with the appearance of a senescent phenotype in human bladder cancer cells	p53,p21,p27	MAPK,p53
Fang, Y.et al.	2013	23723126	1	Cyclin d1 downregulation contributes to anticancer effect of isorhapontigenin on human bladder cancer cells	CCND1,NF-kB，CCNA,CCNB1,CCNE,p53,p21,p27,CDK4,CDK6	P53,cell cycle
Ewald, J. A.et a	2013	23383328	0	Expression microarray meta-analysis identifies genes associated with Ras/MAPK and related pathways in progression of muscle-invasive bladder transition cell carcinoma	COL3A1，COL5A1，COL11A1，FN1，ErbB3，MAPK10和CDC25C	Ras / MAPK
Cogoi, S.et al.	2013	23458775	0	Guanidino anthrathiophenediones as G-quadruplex binders: uptake, intracellular localization, and anti-Harvey-Ras gene activity in bladder cancer cells	HRAS	
Chung, H.et al.	2013	23374291	0	Does phosphorylation of cofilin affect the progression of human bladder cancer?	cofilin	
Chu, H.et al.	2013	23028094	0	EGFR 3'UTR 774T>C polymorphism contributes to bladder cancer risk	hsa-miR-214,EGFR	
Chen, M. C.et a	2013	23416116	0	Magnolol suppresses hypoxia-induced angiogenesis via inhibition of HIF-1alpha/VEGF signaling pathway in human bladder cancer cells	HIF-1α，VEGF，CD31	HIF-1α/ VEGF,AKT / mTOR / p70S6K / 4E-BP-1

Table S1. The list of bladder cancer related articles either used or not used RT4.

Author	Year	PMID	using RT4 or not(0 represents No;1 represents Yes)	Title	Gene	Pathway
Chang, L.et al.	2013	24314030	0	Restoration of LRIG1 suppresses bladder cancer cell growth by directly targeting EGFR activity	EGFR / LRIG1	EGFR / LRIG1,Akt,MAPK
Castiglioni, S.et	2013	23094912	0	N6-isopentenyladenosine and its analogue N6-benzyladenosine induce cell cycle arrest and apoptosis in bladder carcinoma T24 cells	caspase 3	caspase 3
Camargo, E. A.	2013	24289605	1	No relationship between the amount of DNA damage and the level of hMLH1 and RASSF1A gene expression in bladder cancer cells treated with cisplatin and gemcitabine	TP53,Hmlh1,RASSF1A	TP53,Hmlh1,RASSF1A
Burczynska, B.e	2013	24023286	0	Stable knockdown of hCGbeta mRNA expression in bladder cancer cells results in significant growth inhibition	hCGβ	hCGβ
Brassesco, M. S.	2013	23792639	1	In vitro targeting of Polo-like kinase 1 in bladder carcinoma: comparative effects of four potent inhibitors	PLK1 inhibitor	PLK1
Bolenz, C.et al.	2013	23063805	0	Assessing the invasive potential of bladder cancer: development and validation of a new preclinical assay	MMP9,EGFR	MMP9
Bian, J.et al.	2013	23970096	0	Mutation of TGF-beta receptor II facilitates human bladder cancer progression through altered TGF-beta1 signaling pathway	TGF-β 2,P15,CDC25A,CUTL1,Smad2/3	TGF-β
Bi, J. B.et al.	2013	23428845	0	The role of fascin in migration and invasion of urothelial carcinoma of the bladder	Fascin	
Begnini, K. R.et	2013	23053076	0	Auxotrophic recombinant Mycobacterium bovis BCG overexpressing Ag85B enhances cytotoxicity on superficial bladder cancer cells in vitro	Ag85B,Bax,P53	Bax,p53
Backer, C.et al.	2013	24046217	0	Triterpene glycosides from the leaves of Pittosporum angustifolium	药物 A1-巴利果醇糖苷	
Arantes-Rodrigu	2013	24369536	0	Synergistic effect between cisplatin and sunitinib malate on human urinary bladder-cancer cell lines	药物 cisplatin and sunitinib malate	
Arantes-Rodrigu	2013	23582791	0	Meloxicam in the treatment of in vitro and in vivo models of urinary bladder cancer	药物 Meloxicam	
Zoidakis, J et al.	2012	22159600	0	Profilin 1 is a potential biomarker for bladder cancer aggressiveness	Profilin 1,APN,myeloblastin	
Zheng, Q. Y et a	2012	22387548	0	Ursolic acid-induced AMP-activated protein kinase (AMPK) activation contributes to growth inhibition and apoptosis in human bladder cancer T24 cells	PARP,Caspase3,AMPK,ACC,P38,JNK, Survivin,S6K	AMPK-JNK
Zhao, P et al.	2012	22588915	1	Expression of OCT4 protein in bladder cancer and its clinicopathological implications	OCT4,	
Zhang, Y et al.	2012	22343321	0	Cancer stem-like cells contribute to cisplatin resistance and progression in bladder cancer	OCT4,Nanog,Bmi1,CD44	
Zhang, T et al.	2012	22341926	0	Roles of HIF-1alpha in a novel optical orthotopic spontaneous metastatic bladder cancer animal model	HIF-1α,MMP-1,TIMP-1	HIF-1α-MMP-1
Zhang, H. H et a	2012	23111735	0	A proteomic study of potential VEGF-C-associated proteins in bladder cancer T24 cells	VEGF-C,Serpin B5,MMP9,Keratin 5,Annexin A8	

Table S1. The list of bladder cancer related articles either used or not used RT4.

Author	Year	PMID	using RT4 or not(0 represents No;1 represents Yes)	Title	Gene	Pathway
Zhang, H. H et a	2012	21902544	0	RNA interference-mediated vascular endothelial growth factor-C reduction suppresses malignant progression and enhances mitomycin C sensitivity of bladder cancer T24 cells	VEGF-C,Bcl-2,BAX,Caspase-3,MMP-9,P38,AKT,ERK1/2	VEGF-C-AKT/ERK1/2
Zhang, H et al.	2012	21478038	0	Expression of Med19 in bladder cancer tissues and its role on bladder cancer cell growth	Med19	
Zhang, C. J et al	2012	22386417	0	Engagement of integrinbeta1 induces resistance of bladder cancer cells to mitomycin-C	integrin 1,PI3K,AKT	PI3K-Akt
Zhai, Z et al.	2012	22218302	0	Antitumor effects of bladder cancer-specific adenovirus carrying E1A-androgen receptor in bladder cancer	AR,EIA,UPII,PSCAE	
Yang, S et al.	2012	22619087	0	Arctigenin anti-tumor activity in bladder cancer T24 cell line through induction of cell-cycle arrest and apoptosis	CCND1,CDK4,CDK6,ERK1/2,P38	ERK1/2-P38
Wu, M. J et al.	2012	20207175	0	Rictor-dependent AKT activation and inhibition of urothelial carcinoma by rapamycin	AKT,VEGF-A,mTOR,S6K,ERK,CCND1,P27,Rictor,Raptor	PI3K-AKT-mTORC1
Wu, K et al.	2012	22906492	0	PI3K/Akt to GSK3beta/beta-catenin signaling cascade coordinates cell colonization for bladder cancer bone metastasis through regulating ZEB1 transcription	ZEB1,N-cadherin,MMP2,Vimentin,Akt,GSK3β	PI3K-Akt-GSK3β-β-catenin
Watson, C. J et a	2012	22076446	1	Identification of a methylation hotspot in the death receptor Fas/CD95 in bladder cancer	Fas,p53	
Wang, Z. Y et a	2012	20864366	0	Evaluation of thymosin beta4 in the regulation of epithelial-mesenchymal transformation in urothelial carcinoma	thymosin β4,ILK,E-cadherin,β-catenin	
Wang, Y et al.	2012	21993544	0	hsa-miR-96 up-regulates MAP4K1 and IRS1 and may function as a promising diagnostic marker in human bladder urothelial carcinomas	MAP4K1,IRS1,MAP4K2,MAP4K3,MAP4K4	
Wang, X et al.	2012	22844380	0	Methylation and aberrant expression of the Wnt antagonist secreted Frizzled-related protein 1 in bladder cancer	SFRP1,	
Wang, S et al.	2012	23173870	0	Reduced expression of microRNA-100 confers unfavorable prognosis in patients with bladder cancer	miR-100	
Wang, F et al.	2012	22652377	0	Sulforaphane retards the growth of UM-UC-3 xenographs, induces apoptosis, and reduces survivin in athymic mice	Caspase3	
Wang, E et al.	2012	22528227	0	Impact of 4HPR on the expression of E-Cad in human bladder transitional epithelial cancer cells T24	E-cadherin,β-catenin	
Vinall, R. L et a	2012	21702042	0	MiR-34a chemosensitizes bladder cancer cells to cisplatin treatment regardless of p53-Rb pathway status	MiR-34a,p53,CDK6,RB,E2F3,SIRT-1,BCL-2,BCL-XL	p53-Rb
Ueno, K et al.	2012	22057916	0	Tumor suppressor microRNA-493 decreases cell motility and migration ability in human bladder cancer cells by downregulating RhoC and FZD4	miR-493,RhoC,FZD4,Rock-1	
Tatokoro, M et	2012	21964864	0	Potential role of Hsp90 inhibitors in overcoming cisplatin resistance of bladder cancer-initiating cells	CD44,erB2,AKT,ERK,PARP	
Tao, J et al.	2012	21935572	0	microRNA-18a, a member of the oncogenic miR-17-92 cluster, targets Dicer and suppresses cell proliferation in bladder cancer T24 cells	microRNA-18a,DICER	

Table S1. The list of bladder cancer related articles either used or not used RT4.

Author	Year	PMID	using RT4 or not(0 represents No;1 represents Yes)	Title	Gene	Pathway
Szepeshazi, K e	2012	22824624	1	Receptor-targeted therapy of human experimental urinary bladder cancers with cytotoxic LH-RH analog AN-152	LH-RH	
Sun, Z et al.	2012	22609276	0	Beta-eleostearic acid induce apoptosis in T24 human bladder cancer cells through reactive oxygen species (ROS)-mediated pathway	Bcl-2, BaxCaspase-3, PPARγ	ROS-mediated pathway
Sun, F et al.	2012	22482362	0	Potential anticancer activity of myricetin in human T24 bladder cancer cells both in vitro and in vivo	CCNB1,CDC2,AKT,BAX,BCL-2MMP9,Caspase3,P38	MAPK
Sima, J et al.	2012	22781569	0	Expression analysis of NOTCH1/HES1/PTEN signaling pathway in invasive bladder transitional cell carcinoma	NOTCH1,HES1,PTEN	NOTCH1-HES1-PTEN
Shiota, M et al.	2012	22608749	1	Androgen receptor signaling regulates cell growth and vulnerability to doxorubicin in bladder cancer	AR,Lamin B1,CCND1	
Qin, J et al.	2012	22941057	0	Epigallocatechin-3-gallate inhibits bladder cancer cell invasion via suppression of NF-kappaBmediated matrix metalloproteinase-9 expression	NF-Kb,MMP-9,NF-κB	NF-κB
Ping, S. Y et al.	2012	20884251	0	Sunitinib can enhance BCG mediated cytotoxicity to transitional cell carcinoma through apoptosis pathway	BCL-2，BCL-XL,VEGF	
Peek, E. M et al	2012	23226620	0	Stromal modulation of bladder cancer-initiating cells in a subcutaneous tumor model	CK20,CK5,CD44,EGFR,S6K,E-cadherin,N-cadherin	
Overdevest, J. E	2012	23012401	0	CD24 expression is important in male urothelial tumorigenesis and metastasis in mice and is androgen regulated	CD24,AR	
Otto, K. B et al.	2012	22154358	1	Stress-activated kinase pathway alteration is a frequent event in bladder cancer	MKK3,MKK4,MKK6,MKK7,P38,JNK	MAPK
Miyake, M et al	2012	22928931	1	Influencing factors on the NMP-22 urine assay: an experimental model	NMP22	
McKenna, D. J	2012	23145163	1	Use of the comet-FISH assay to compare DNA damage and repair in p53 and hTERT genes following ionizing radiation	Htert,p53	
Maruyama, T et	2012	22041988	0	Apoptosis of bladder cancer by sodium butyrate and cisplatin	P21,P27,FADD,TRADD,Caspase-2.Caspase-7	
Liu, Z et al.	2012	21520297	1	Rhodiola rosea extracts and salidroside decrease the growth of bladder cancer cell lines via inhibition of the mTOR pathway and induction of autophagy	TSC2,p53,AMPK,ACC,4EBP1,S6K,eIF4E,mTOR	mTOR pathway
Liu, Y et al.	2012	23284967	0	Synthetic miRNA-mowers targeting miR-183-96-182 cluster or miR-210 inhibit growth and migration and induce apoptosis in bladder cancer cells	miR-183-96–182,miR-210,PI3K,AKT, mTOR	
Liu, S. Y et al.	2012	23217386	0	MEK1 and MEK2 differentially regulate human insulin- and insulin glargine-induced human bladder cancer T24 cell proliferation	MEK1,MEK2,ERK1/2,AKT	MEK-ERK
Lin, Y et al.	2012	22289176	0	Cyclin-dependent kinase 4 is a novel target in micoRNA-195-mediated cell cycle arrest in bladder cancer cells	miR-195,CDK4,CDK6,CCND1,E2F3,Rb	
Li, Y et al.	2012	22041818	0	Effects of YC-1 on hypoxia-inducible factor 1 alpha in hypoxic human bladder transitional carcinoma cell line T24 cells	HIF1-α,VEGF,MMP-2,BCL-2	ERK/p38-MAPK

Table S1. The list of bladder cancer related articles either used or not used RT4.

Author	Year	PMID	using RT4 or not(0 represents No;1 represents Yes)	Title	Gene	Pathway
Li, L et al.	2012	22744428	0	Chemotherapy sorting can be used to identify cancer stem cell populations	OCT-4,NANOG	
Li, J et al.	2012	22460090	0	Increased expression of DNA repair gene XPF enhances resistance to hydroxycamptothecin in bladder cancer	XPF,Caspase3,Caspase8,Caspase9	
Li, B et al.	2012	22294235	0	Chlorophyllin e4 is a novel photosensitizer against human bladder cancer cells	Chlorophyllin e4	
Lee, S. J et al.	2012	22560878	0	Interleukin-28A triggers wound healing migration of bladder cancer cells via NF-kappaB-mediated MMP-9 expression inducing the MAPK pathway	IL-28A,MMP-9,MMP-2,NF-K b,ERK1/2,p38MAPK	MAPK
Kihira, S et al.	2012	23213380	0	Membrane microdomain-associated uroplakin IIIa contributes to Src-dependent mechanisms of anti-apoptotic proliferation in human bladder carcinoma cells	SRC,MET,P145,P60,EGFR,UPIII,Y41 8,Y529,MAPK	tyrosine kinase signaling pathway
Kanno, T et al.	2012	23171836	0	A(3) adenosine receptor mediates apoptosis in 5637 human bladder cancer cells by G(q) protein/PKC-dependent AIF upregulation	AIF,AR,AMID,BAX,BID,PUMA,NOX A,BCL-2,BCL-XL,P53,MCL-1,HRK	
Kang, M. R et a	2012	22740865	0	Gene expression profiling of KBH-A42, a novel histone deacetylase inhibitor, in human leukemia and bladder cancer cell lines	HRK,TNFRSF10B,PYCARD,TNFRSF 8	
Jou, Y. C et al.	2012	22559731	0	Expression of protein kinase Calpha and the MZF-1 and elk-1 transcription factors in human bladder transitional cell carcinoma cells	MZF-1,ELK-1,PKCα	
Jiang, B et al.	2012	22554590	1	Inhibition of fatty-acid synthase suppresses P-AKT and induces apoptosis in bladder cancer	FASN,Caspase-3,Caspase-7,Caspase-8,BCL-2,CCND1,AKT	PI3K-AKT
Jayasooriya, R.	2012	21993858	0	Methanol extract of Hydroclathrus clathratus suppresses matrix metalloproteinase-9 in T24 bladder carcinoma cells by suppressing the NF-kappaB and MAPK pathways	MMP-9,TNF-α,MMP-9,NF-κB ,PI3K, Akt,P50,P65,ERK,P38,JNK	NF-κB and MAPK pathways
Izumi, K et al.	2012	22922989	0	Epidermal growth factor induces bladder cancer cell proliferation through activation of the androgen receptor	EGFR,AR,TIF2,ER,	AR and EGFR pathways
Huang, W. W et	2012	22285700	1	Bufalin induces G0/G1 phase arrest through inhibiting the levels of cyclin D, cyclin E, CDK2 and CDK4, and triggers apoptosis via mitochondrial signaling pathway in T24 human bladder cancer cells	CCND,CCNE,CDK2,CDK4,RB,Caspas e-3, Caspase-7,Caspase-9,AIF,APAF-1,GSK-3α/β,BAX,BCL-2,AKT,	mitochondria signaling pathway
Huang, W. W et	2012	22272214	0	Cucurbitacin E Induces G(2)/M Phase Arrest through STAT3/p53/p21 Signaling and Provokes Apoptosis via Fas/CD95 and Mitochondria-Dependent Pathways in Human Bladder Cancer T24 Cells	STAT3,p53,p21,FAS,CD95,CDK1,P2 1,CCNB,Caspase-3,Caspase-8,Caspase-9,Cytochrome c,Apaf-1,AIF,Fas/CD95,FASL	Fas/CD95 and Mitochondria-Dependent Pathways
Huang, H. S et ā	2012	22728270	1	TG-interacting factor-induced superoxide production from NADPH oxidase contributes to the migration/invasion of urothelial carcinoma	TGIF,P67,NOX2,NOX3,MMP2,TGIF, AKT,	PI3K/AKT pathway
Hong, S. H et al	2012	21901250	0	Bufalin induces apoptosis through activation of both the intrinsic and extrinsic pathways in human bladder cancer cells	Bax,Bcl-2,Bcl-xL,XIAP,cIAP-1,cIAP-2,Fas,FasL,DR4,DR5,TRAIL,Caspase-3,Caspase-8,Caspase-9,PARP	mitochondrial mediated-intrinsic caspase pathway and the death receptor-mediated extrinsic pathway

Table S1. The list of bladder cancer related articles either used or not used RT4.

Author	Year	PMID	using RT4 or not(0 represents No;1 represents Yes)	Title	Gene	Pathway
Hirata, T et al.	2012	22665039	1	REIC/Dkk-3-encoding adenoviral vector as a potentially effective therapeutic agent for bladder cancer	REIC,JNK,P-GP	JNK pathway
Hirata, H et al.	2012	22049531	0	MicroRNA-1826 targets VEGFC, beta-catenin (CTNNB1) and MEK1 (MAP2K1) in human bladder cancer	miR-1826,VEGFC, CTNNB1,MEK1,Survivin,	MEK-ERK and Wnt-beta-catenin signaling pathway
He, Y. F. et al.	2012	22902906	0	Upregulation of cell adhesion through delta Np63 silencing in human 5637 bladder cancer cells	Np63,	
Han, B et al.	2012	22238119	1	Estrogen receptor beta (ERbeta) is a novel prognostic marker of recurrence survival in non-muscle-invasive bladder cancer potentially by inhibiting cadherin switch	ERβ,E-cadherin,N-cadherin	
Guo, Y et al.	2012	23741253	0	miR-96 regulates FOXO1-mediated cell apoptosis in bladder cancer	FOXO1,miR-96	
Gotoh, A et al.	2012	23007551	0	Antitumor action of alpha(1)-adrenoceptor blockers on human bladder, prostate and renal cancer cells	AR	
Fokas, E et al.	2012	22452803	0	NVP-BEZ235 and NVP-BGT226, dual phosphatidylinositol 3-kinase/mammalian target of rapamycin inhibitors, enhance tumor and endothelial cell radiosensitivity	mTOR,AKT,S6K,	PI3K-Akt pathway
Fishman, A. I. e	2012	22393334	0	Additively enhanced antiproliferative effect of interferon combined with proanthocyanidin on bladder cancer cells	CDK2, CDK4, CCND1,CCNE,P27	
Fei, X et al.	2012	22265971	0	MicroRNA-195-5p suppresses glucose uptake and proliferation of human bladder cancer T24 cells by regulating GLUT3 expression	GLUT3,miR-195-5p	
Ding, G. Q et al	2012	22556170	0	Antitumor effects of human interferon-alpha 2b secreted by recombinant bacillus Calmette-Guerin vaccine on bladder cancer cells	IFNα-2b	
DeGraff, D. J et	2012	22590586	1	Loss of the urothelial differentiation marker FOXA1 is associated with high grade, late stage bladder cancer and increased tumor proliferation	FOXA1,FOXA3,UPK1A,UPK1B,UPK2,UPK3A,UPK3B,E-cadherin	
da Silva, G. N e	2012	23053941	0	Toxicogenomic activity of gemcitabine in two TP53-mutated bladder cancer cell lines: special focus on cell cycle-related genes	BRCA1, CCNE1, CDK2, CDK6, CDKN1A, CDKN2B, E2F4, GADD45A, MAD2L2, CCNH, SERTAD1, CDC1, CHEK1	
Coon, B. G et al	2012	21901746	0	Fibronectin attachment protein from bacillus Calmette-Guerin as targeting agent for bladder tumor cells	FAP,Cav1,Chc	
Chen, X et al.	2012	22895653	0	beta-elemene acts as an antitumor factor and downregulates the expression of survivin, Bcl-xL and Mta-1	survivin,Bcl-xL,Mta-1	
Chen, H et al.	2012	22266187	0	MicroRNA-449a acts as a tumor suppressor in human bladder cancer through the regulation of pocket proteins	miR-449a,CDK6,CDC25a,E2F1,Rb,P130	Rb phosphorylation pathway
Chen, D et al.	2012	22462738	0	Capsaicin induces cycle arrest by inhibiting cyclin-dependent-kinase in bladder carcinoma cells	TRPV1,CDK2,CDK4,CDK6	
Byler, T. K et al	2012	22898175	0	Valproic acid decreases urothelial cancer cell proliferation and induces thrombospondin-1 expression	HSP 90,BAX,TSP-1,HO-1	
Brassesco, M. S	2012	22901154	0	Cytostatic in vitro effects of DTCM-glutarimide on bladder carcinoma cells	PDK1,	

Table S1. The list of bladder cancer related articles either used or not used RT4.

Author	Year	PMID	using RT4 or not(0 represents No;1 represents Yes)	Title	Gene	Pathway
Bhattacharya, A	2012	22131350	0	The principal urinary metabolite of allyl isothiocyanate, N-acetyl-S-(N-allylthiocarbamoyl)cysteine, inhibits the growth and muscle invasion of bladder cancer	Caspase-3,VEGF,	
Bednarek, I et a	2012	23788901	0	The efficiency of silencing expression of the gene coding STAT3 transcriptional factor and susceptibility of bladder cancer cells to apoptosis	STAT3,	STAT3 signaling pathways
Asadzadeh, J et	2012	22903480	0	A plausible anti-apoptotic role of up-regulated OCT4B1 in bladder tumors	OCT4B1	
Al-Azayzih, A e	2012	22989755	0	TGFbeta1 induces apoptosis in invasive prostate cancer and bladder cancer cells via Akt-independent, p38 MAPK and JNK/SAPK-mediated activation of caspases	TGF β,P38,MAPK,JNK,Akt,ERK1/2,Caspase-3,Caspase-8,Caspase-9	p38MAPK and SAPK/JNK pathways
Abbaoui, B et a	2012	23038615	1	Inhibition of bladder cancer by broccoli isothiocyanates sulforaphane and erucin: characterization, metabolism, and interconversion	PARP,survivin,EGFR,HER2,	
Zhu, Y et al.	2011	21147498	1	ncRAN, a newly identified long noncoding RNA, enhances human bladder tumor growth, invasion, and survival	ncRAN	
Zheng Y et al.	2011	21613411	1	Dihydrotestosterone upregulates the expression of epidermal growth factor receptor and ERBB2 in androgen receptor-positive bladder cancer cells	EGFR 、ERBB2、ERK1/2、AKT	EGFR/ERBB2
Zhang J et al.	2011	20845808	0	Glycosyl-phosphatidylinositol-anchored interleukin-2 expressed on tumor-derived exosomes induces anti-tumor immune response	IL-2、GPI-IL-2、MAGE-1	
Zeng J et al.	2011	21220495	0	Chemopreventive and chemotherapeutic effects of intravesical silibinin against bladder cancer by acting on mitochondria	MNU	
Yuan X et al.	2011	21505989	0	Effect of silencing LRIG3 gene on the proliferation and apoptosis of bladder cancer T24 cells	LRIG3、	
Yoshitomi T et a	2011	21479368	0	Restoration of miR-517a expression induces cell apoptosis in bladder cancer cell lines	BCLAF1	
Yoshida S et al.	2011	22134243	0	Low-dose Hsp90 inhibitors tumor-selectively sensitize bladder cancer cells to chemoradiotherapy	erbB2, Akt, NF-κB	
Yoon CY et al.	2011	21344032	1	Sunitinib malate synergistically potentiates anti-tumor effect of gemcitabine in human bladder cancer cells	cyclin B1, p-Akt, t-Ak	
Yeh, C. Y. et al.	2011	21422242	1	Transcriptional activation of the Axl and PDGFR-alpha by c-Met through a ras- and Src-independent mechanism in human bladder cancer	PDGFR-alpha、c-Met、Axl PDGFR-alpha	
Yano, K.et al.	2011	21165560	1	Chetomin induces degradation of XIAP and enhances TRAIL sensitivity in urogenital cancer cells	TRAIL、XIAP	
Yang, W. et al.	2011	21422242	0	Quantitative proteomics identifies a beta-catenin network as an element of the signaling response to Frizzled-8 protein-related antiproliferative factor	APF、COX-2	
Yan K et al.	2011	21545798	0	Induction of G1 cell cycle arrest and apoptosis by berberine in bladder cancer cells	H-Ras、c-fos	

Table S1. The list of bladder cancer related articles either used or not used RT4.

Author	Year	PMID	using RT4 or not(0 represents No;1 represents Yes)	Title	Gene	Pathway
Yamamoto H et	2011	21421245	0	Requirement for FBP17 in invadopodia formation by invasive bladder tumor cells	FBP17	
Xue YJ et al.	2011	20509007	0	CD147 overexpression is a prognostic factor and a potential therapeutic target in bladder cancer	MMP-2、MMP-9、VEGF	
Xue Y et al.	2011	22110753	0	Twisted epithelial-to-mesenchymal transition promotes progression of surviving bladder cancer T24 cells with hTERT-dysfunction	hTERT	
Wu, S. Y. et al.	2011	22241963	0	Ras-related tumorigenesis is suppressed by BNIP3-mediated autophagy through inhibition of cell proliferation	BNIP3, Atg5, Raf-1，ERK，H-ras	Raf-1/ERK
Wu G et al.	2011	21778192	0	High-mobility group protein N2 (HMGN2) inhibited the internalization of Klebsiella pneumoniae into cultured bladder epithelial cells	ERK1/2，HMGN2	
Wszolek MF et a	2011	19945312	0	A MicroRNA expression profile defining the invasive bladder tumor phenotype	miR-30b, miR-31, miR-141, miR-200a, miR-200b, miR-200c, miR-205, miR-21	
Wang ZQ et al.	2011	22019956	1	Specific survivin dual fluorescence resonance energy transfer molecular beacons for detection of human bladder cancer cells	HDF-a	
Wang X et al.	2011	21053100	0	Short interfering RNA directed against Slug blocks tumor growth, metastasis formation, and vascular leakage in bladder cancer	Slug，MMP-2	
vom Dorp F et a	2011	21051874	1	Inhibition of Rho-kinase abrogates migration of human transitional cell carcinoma cells: results of an in vitro study	Rho	
Verma A et al.	2011	20735383	0	Effect of mitomycin C on concentrations of vascular endothelial growth factor and its receptors in bladder cancer cells and in bladders of rats intravesically instilled with mitomycin C	VEGF, survivin, VEGF receptor-1 (VEGFR-1)， VEGFR-2	
Vassou, D. et al.	2011	21643624	1	Biodistribution and photodynamic effects of polyvinylpyrrolidone-hypericin using multicellular spheroids composed of normal human urothelial and T24 transitional cell carcinoma cells	B2 receptor	
van der Horst, C	2011	21616583	0	Real-time cancer cell tracking by bioluminescence in a preclinical model of human bladder cancer growth and metastasis	luc2	
Vallo S et al.	2011	21822119	0	HDAC inhibition delays cell cycle progression of human bladder cancer cells in vitro	cdk1, cdk2, cdk4, and cyclins B, D1, E	
Tsang M et al.	2011	21417694	0	Effects of increasing carbon nanofiber density in polyurethane composites for inhibiting bladder cancer cell functions	VEGF	
Tolg C et al.	2011	21242958	0	Uropathogenic E. coli infection provokes epigenetic downregulation of CDKN2A (p16INK4A) in uroepithelial cells	CDH1, MLH1, DAPK1，TLR4	
Thomas, S. et al	2011	21148751	0	Src and caveolin-1 reciprocally regulate metastasis via a common downstream signaling pathway in bladder cancer	Cav-1	
Tao, J. et al.	2011	21468550	0	microRNA-21 modulates cell proliferation and sensitivity to doxorubicin in bladder cancer cells	BCL-2, AKT，pAKT	
Tanaka N et al.	2011	21970881	0	Acquired platinum resistance enhances tumour angiogenesis through angiotensin II type 1 receptor in bladder cancer	VEGF	

Table S1. The list of bladder cancer related articles either used or not used RT4.

Author	Year	PMID	using RT4 or not(0 represents No;1 represents Yes)	Title	Gene	Pathway
Szliszka E et al.	2011	24578888	0	Effect of ALA-mediated photodynamic therapy in combination with tumor necrosis factor-related apoptosis-inducing ligand (TRAIL) on bladder cancer cells	TRAIL	
Stravopodis, D.	2011	20425122	0	Thymidylate synthase inhibition induces p53-dependent and p53-independent apoptotic responses in human urinary bladder cancer cells	E2F1、Rb	RB- E2F1
Song, T. et al.	2011	21790228	1	Expression of miR-143 reduces growth and migration of human bladder carcinoma cells by targeting cyclooxygenase-2	COX-2、miR-143	
Smith NJet al.	2011	21784459	1	Toll-like receptor responses of normal human urothelial cells to bacterial flagellin and lipopolysaccharide	interleukin-6	
Shiota M et al.	2011	21083641	0	Twist1 and Y-box-binding protein-1 promote malignant potential in bladder cancer cells	YB-1、Twist1	
Shiota M et al.	2011	20726978	1	Enhanced S100 calcium-binding protein P expression sensitizes human bladder cancer cells to cisplatin	S100P	
Shimada, K. et a	2011	21592330	0	Cyclooxygenase 2-dependent and independent activation of Akt through casein kinase 2alpha contributes to human bladder cancer cell survival	COX2	
Shen KH et al.	2011	21196431	1	Isolinderanolide B, a butanolide extracted from the stems of Cinnamomum subavenium, inhibits proliferation of T24 human bladder cancer cells by blocking cell cycle progression and inducing apoptosis	p53, p21 Waf1/Cip1, Fas/APO-1 receptor, Fas	
Shanmugam, R.	2011	20669221	1	A water soluble parthenolide analog suppresses in vivo tumor growth of two tobacco-associated cancers, lung and bladder cancer, by targeting NF-kappaB and generating reactive oxygen species	TRAF-2， XIAP. DMAPT	
Schepmann, D.	2011	21550749	0	Expression of sigma receptors of human urinary bladder tumor cells (RT-4 cells) and development of a competitive receptor binding assay for the determination of ligand affinity to human sigma(2) receptors	DTG	
Schedel F et al.	2011	21240463	0	mTOR inhibitors show promising in vitro activity in bladder cancer and head and neck squamous cell carcinoma	mTOR	
Roelants, M. et a	2011	27811523	0	Use of fluorescein isothiocyanate-human serum albumin for the intravesical photodiagnosis of non-muscle-invasive bladder cancer: an in vitro study using multicellular spheroids composed of normal human urothelial and urothelial cell carcinoma cell lines	HSA	
Roelants, M. et a	2011	21103512	0	Human serum albumin as key mediator of the differential accumulation of hypericin in normal urothelial cell spheroids versus urothelial cell carcinoma spheroids	mTHPP	
Rassouli FB et a	2011	21126568	0	Investigating the enhancement of cisplatin cytotoxicity on 5637 cells by combination with mogoltacin	cisplatin	
Ramachandran,	2011	22110197	0	5-azacytidine reverses drug resistance in bladder cancer cells	TMS1	

Table S1. The list of bladder cancer related articles either used or not used RT4.

Author	Year	PMID	using RT4 or not(0 represents No;1 represents Yes)	Title	Gene	Pathway
Qu G et al.	2011	22368873	0	Effects of pseudolaric acid B on apoptosis of bladder cancer cell 5637	Pseudolaric acid B	
Plissonnier, M.	2011	22174792	0	The antidiabetic drug ciglitazone induces high grade bladder cancer cells apoptosis through the up-regulation of TRAIL	p53, p21(waf1/CIP1)，p27(Kip1)，cyclin B1	
Oliveira, J. C. et	2011	22393979	0	MicroRNA-100 acts as a tumor suppressor in human bladder carcinoma 5637 cells	miR-100 、miR-708	
Nordentoft, I. e	2011	21489314	0	Increased expression of transcription factor TFAP2alpha correlates with chemosensitivity in advanced bladder cancer	TFAP2alpha	
Noguchi S et al.	2011	21550168	0	MicroRNA-143 functions as a tumor suppressor in human bladder cancer T24 cells	ERK5 、Akt 、miR-143	
Nikpour P et al.	2011	20477901	1	The RNA binding protein Musashi1 regulates apoptosis, gene expression and stress granule formation in urothelial carcinoma cells	p21(CIP1)、p27(KIP1)	
Nakahara, T. et	2011	21205082	0	Broad spectrum and potent antitumor activities of YM155, a novel small-molecule survivin suppressant, in a wide variety of human cancer cell lines and xenograft models	YM155	
Murali A et al.	2011	21569442	1	Adenoviral infectivity of exfoliated viable cells in urine: implications for the detection of bladder cancer	Ad.Surv.	
Mugabe, C. et a	2011	21357680	0	In vivo evaluation of mucoadhesive nanoparticulate docetaxel for intravesical treatment of non-muscle-invasive bladder cancer	DTX	
Mollazadeh S et	2011	22351980	0	Feselol enhances the cytotoxicity and DNA damage induced by cisplatin in 5637 cells	Ferula	
Miyazaki, J. et a	2011	21314815	0	The liposome-incorporating cell wall skeleton of Mycobacterium bovis bacillus Calmette-Guein can directly enhance the susceptibility of cancer cells to lymphokine-activated killer cells through up-regulation of natural-killer group 2, member D ligands	UL-16-binding protein	
Miyake, M. et a	2011	21206978	0	Heme oxygenase-1 promotes angiogenesis in urothelial carcinoma of the urinary bladder	(HIF)-1alpha, HIF-2alpha, cyclooxygenase-2 (COX-2), interleukin-8 (IL-8), basic fibroblast growth factor (bFGF)	
Memon AA et a	2011	21087080	0	Calcium-induced apoptosis is delayed by HER1 receptor signalling through the Akt and PLCgamma pathways in bladder cancer cells	Akt, PLCγ and MAPK	
Membrino, A. e	2011	21931711	0	G4-DNA formation in the HRAS promoter and rational design of decoy oligonucleotides for cancer therapy	MAZ，Sp1	
Maruyama, T. e	2011	20632198	0	Double short-time exposure to pirarubicin produces higher cytotoxicity against T24 bladder cancer cells	THP	
Makhlin I et al.	2011	21050361	0	The mTOR pathway affects proliferation and chemosensitivity of urothelial carcinoma cells and is upregulated in a subset of human bladder cancers	p-mTOR	
Liu T et al.	2011	21473288	0	iASPP is important for bladder cancer cell proliferation	iASPP	

Table S1. The list of bladder cancer related articles either used or not used RT4.

Author	Year	PMID	using RT4 or not(0 represents No;1 represents Yes)	Title	Gene	Pathway
Liu, S. et al.	2011	21129803	0	High dose human insulin and insulin glargine promote T24 bladder cancer cell proliferation via PI3K-independent activation of Akt	PI3K、MEK	
Liu, H. S. et al.	2011	21757545	0	Curcumin-induced mitotic spindle defect and cell cycle arrest in human bladder cancer cells occurs partly through inhibition of aurora A	Aurora	
Li Y et al.	2011	21529900	0	Pazopanib synergizes with docetaxel in the treatment of bladder cancer cells	phospho-AKT, phospho-FAK, total AKT, total FAK	
Li X et al.	2011	21176918	0	Tetrandrine induces apoptosis and triggers caspase cascade in human bladder cancer cells	caspase-9, caspase-8, caspase-3，PARP	
Li S et al.	2011	24212635	0	Ras Isoprenylation and pAkt Inhibition by Zoledronic Acid and Fluvastatin Enhances Paclitaxel Activity in T24 Bladder Cancer Cells	Ras/Raf/MEK/ERK /PI3K/AKT	Ras/Raf/MEK/ERK，PI3K/AKT
Li, J. et al.	2011	21054790	0	Fisetin, a dietary flavonoid, induces cell cycle arrest and apoptosis through activation of p53 and inhibition of NF-kappa B pathways in bladder cancer cells	cyclin D1, cyclin A, CDK4，CDK2,	
Buraschi S et al.	2011	21567105	0	Suppression of bladder cancer cell tumorigenicity in an athymic mouse model by adenoviral vector-mediated transfer of LRIG1	EGFR	
Li F, et al.	2011	21505987	0	Evaluation of tumor formation of three bladder cancer cell lines in nude mice	PCNA	
Li F et al.	2011	21431282	0	LRIG1 combined with cisplatin enhances bladder cancer lesions via a novel pathway	LRIG1、EGFR	
Lekka M et al.	2011	21812057	1	Characterization of N-cadherin unbinding properties in non-malignant (HCV29) and malignant (T24) bladder cells	N-cadherin	
Lachmann S et a	2011	21754995	1	Regulatory domain selectivity in the cell-type specific PKN-dependence of cell migration	PKN2	
Kovala-Demertz	2011	21718655	1	Synthesis, crystal structures and spectroscopy of meclofenamic acid and its metal complexes with manganese(II), copper(II), zinc(II) and cadmium(II). Antiproliferative and superoxide dismutase activity	C-H--π	
Kim, J. et al.	2011	21136194	1	An hTERT-immortalized human urothelial cell line that responds to anti-proliferative factor	APF	
Kenney PA et al	2011	20735391	0	Novel ZEB1 expression in bladder tumorigenesis	ZEB1	
Kawashima, A.	2011	21177407	1	Excision repair cross-complementing group 1 may predict the efficacy of chemoradiation therapy for muscle-invasive bladder cancer	(ERCC1	
Kashiwagi E et a	2011	21087353	0	Enhanced expression of nuclear factor I/B in oxaliplatin-resistant human cancer cell lines	OX2	
Karam JA et al.	2011	20734393	1	Upregulation of TRAG3 gene in urothelial carcinoma of the bladder	TRAG3	
Jin, B. et al.	2011	20803103	0	Anti-tumour efficacy of mitofusin-2 in urinary bladder carcinoma	Mfn2	

Table S1. The list of bladder cancer related articles either used or not used RT4.

Author	Year	PMID	using RT4 or not(0 represents No;1 represents Yes)	Title	Gene	Pathway
Jeon, H. G. et al	2011	21944112	0	Induction of caspase mediated apoptosis and down-regulation of nuclear factor-kappaB and Akt signaling are involved in the synergistic antitumor effect of gemcitabine and the histone deacetylase inhibitor trichostatin A in human bladder cancer cells	p-Akt, Akt, p-mTOR, mTOR，PTEN，p21(WAF1/CIP1), cyclin A, B1 ，D1, p-CDC2C, CDC2C, p-CDC25C, CDC25C and pRb	
Huang, Y. C. et	2011	21741467	0	para-Phenylenediamine-induced autophagy in human uroepithelial cell line mediated mutant p53 and activation of ERK signaling pathway	ERK1/2	
Huang, Y. C.et a	2011	21167264	0	Effects of MEK and DNMT inhibitors on arsenic-treated human uroepithelial cells in relation to Cyclin-D1 and p16	Cyclin-D1	
Hu Z et al.	2011	19767220	0	Fibulin-5 is down-regulated in urothelial carcinoma of bladder and inhibits growth and invasion of human bladder cancer cell line 5637	fibulin-5	
Han Y et al.	2011	22393963	0	Aberrant FHIT expression is linked to bladder carcinogenesis and apoptosis	FHIT	
Griffith, T. S. et	2011	21754995	0	Sensitization of human bladder tumor cells to TNF-related apoptosis-inducing ligand (TRAIL)-induced apoptosis with a small molecule IAP antagonist	XIAP	
Gou X et al.	2011	22812186	1	Gene silence-induced downregulation of survivin inhibits bladder cancer cells	BAX， BAD	
Eruslanov, E. et	2011	21315786	0	Aberrant PGE(2) metabolism in bladder tumor microenvironment promotes immunosuppressive phenotype of tumor-infiltrating myeloid cells	CD11b	
Dufresne, M. et	2011	21601924	1	Pro-inflammatory type-1 and anti-inflammatory type-2 macrophages differentially modulate cell survival and invasion of human bladder carcinoma T24 cells	Mvarphi-2	
Du, P. et al.	2011	21965727	0	Metastasis suppressor-1, MTSS1, acts as a putative tumour suppressor in human bladder cancer	MTSS1	
da Silva GN et a	2011	21116856	1	Expression of genes related to apoptosis, cell cycle and signaling pathways are independent of TP53 status in urinary bladder cancer cells	TP53	
Chuang, C. H. e	2011	22346685	1	System-level biochip for impedance sensing and programmable manipulation of bladder cancer cells	DEP	
Chiong E et al.	2011	21415218	1	Effects of mTOR inhibitor everolimus (RAD001) on bladder cancer cells	RAD001	
Cheng G et al.	2011	21829151	0	Parthenolide induces apoptosis and cell cycle arrest of human 5637 bladder cancer cells in vitro	Bcl-2	
Chang X et al.	2011	21698293	0	Adenylate kinase 3 sensitizes cells to cigarette smoke condensate vapor induced cisplatin resistance	AK3	
Chang LC et al.	2011	21870460	0	Preparation, characterization and cytotoxicity evaluation of tanshinone IIA nanoemulsions	TA-NEs	
Cao, Y. et al.	2011	20857258	0	MicroRNA-dependent regulation of PTEN after arsenic trioxide treatment in bladder cancer cell line T24	PTEN、miRNA-19a	

Table S1. The list of bladder cancer related articles either used or not used RT4.

Author	Year	PMID	using RT4 or not(0 represents No;1 represents Yes)	Title	Gene	Pathway
Canetta E et al.	2011	21456875	0	Modulated Raman spectroscopy for enhanced identification of bladder tumor cells in urine samples	SRS	
Caiazzo RJ Jr et	2011	21370063	0	Native antigen fractionation protein microarrays for biomarker discovery	PBS	
Awsare NS et al	2011	21468549	0	Claudin-11 decreases the invasiveness of bladder cancer cells	Claudin-11	
Adhim, Z. et al.	2011	21750550	0	In vitro and in vivo inhibitory effect of three Cox-2 inhibitors and epithelial-to-mesenchymal transition in human bladder cancer cell lines	E-cadherin	
Aaltonen V, Pel	2010	21036713	0	PKCalpha/beta I inhibitor Go6976 induces dephosphorylation of constitutively hyperphosphorylated Rb and G1 arrest in T24 cells	Rb,cdc2	
Bai Y, Mao QQ.	2010	20028382	0	Resveratrol induces apoptosis and cell cycle arrest of human T24 bladder cancer cells in vitro and inhibits tumor growth in vivo	p38,akt,pakt,ccnd1,cdk4,rb,bcl-2,bax,bad,parp,caspase-3,caspase-9,vegf,fgf2	AKT, p38 MAPK
Caldwell JA, Di	2010	20212456	0	Development of a continuous assay for the measurement of tissue factor procoagulant activity on intact cells	TF	
Chen CL, Chan	2010	20682636	0	Elevated expression of protein kinase C delta induces cell scattering upon serum deprivation	PKCdelta, c-Jun,JNK,p38,ERK	ROS-Src-PKCd-JNK
Chen NG, Chen	2010	20596632		Allyl isothiocyanate triggers G2/M phase arrest and apoptosis in human brain malignant glioma GBM 8401 cells through a mitochondria-dependent pathway	CDK1,cyclin B,caspase-3,caspase-8,caspase-9,apaf1,cytochrome c,AIF,Endo G,cyclin B	cell cycle, apoptotic signaling
Chen RJ, Ho YS	2010	20106947	0	Long-term nicotine exposure-induced chemoresistance is mediated by activation of Stat3 and downregulation of ERK1/2 via nAChR and beta-adrenoceptors in human bladder cancer cells	Stat3,Cyclin D1,ERK 1/2,Cyclin A, Cyclin B,PCNA,phospho-cdc2, Bcl-2, Bax,PARP,nAChR,β-AR	Stat3,ERK1/2
Gao XD, Chen `	2010	20423850	0	[Effect of hTERT antisense oligodeoxynucleotide on telomerase activity in bladder cancer cells in vitro]	hTERT	
Hansel DE, Plat	2010	20395440	1	Mammalian target of rapamycin (mTOR) regulates cellular proliferation and tumor growth in urothelial carcinoma	mTOR,P-S6,PARP ,AKT,TSC1,S6K,PTEN	mTOR
He Y, Wu X, Lu	2010	20205955	0	Functional significance of the hepaCAM gene in bladder cancer	hepaCAM	
Huang YT, Lai	2010	20428771	0	BDNF mediated TrkB activation is a survival signal for transitional cell carcinoma cells	BDNF,TrkB,TrkA,TrkC,ERK2	
Jang TJ, Cha W.	2010	20582552	0	Reciprocal correlation between the expression of cyclooxygenase-2 and E-cadherin in human bladder transitional cell carcinomas	COX-2,E-cadherin,SLUG	COX-2
Ju ZH, Ying MC	2010	20450613	1	[Effects of 5-Aza-2'-deoxycytidine and trichostatin A on expression and apoptosis of ALDH1a2 gene in human bladder cancer cell lines]	ALDH1a2	
Juengel E, Beec	2010	20336301	1	Maspin modulates adhesion of bladder carcinoma cells to vascular endothelium	maspin	
Kanai K, Kikucl	2010	19824995	0	Vitamin E succinate induced apoptosis and enhanced chemosensitivity to paclitaxel in human bladder cancer cells in vitro and in vivo	NF-kappaB,Ki-67,IκBα,c-IAP1	

Table S1. The list of bladder cancer related articles either used or not used RT4.

Author	Year	PMID	using RT4 or not(0 represents No;1 represents Yes)	Title	Gene	Pathway
Karkoulis PK, S	2010	20828379	1	17-Allylamino-17-demethoxygeldanamycin induces downregulation of critical Hsp90 protein clients and results in cell cycle arrest and apoptosis of human urinary bladder cancer cells	IGF-IR, Akt, IKK-α, IKK-β, FOXO1, ERK1/2,c-Met,CDK4,cyclin D1,E2F1,Rb,caspase-3,caspase-8,caspase-9,PARP,HSP90,HSP70,CHIP,	cell cycle, NF-κB
Kim H, Kang JV	2010	20683018	0	A3 adenosine receptor antagonist, truncated Thio-Cl-IB-MECA, induces apoptosis in T24 human bladder cancer cells	cyclin A, cyclin D1, cyclin E，Rb，PARP, caspase-3, caspase-9, Bax, Bcl-2, cytochrome c, phospho-ERK1/2, phospho-SAPK/JNK,p53,p21,p27	MAPK,cell cycle
Ku JH, Seo SY,	2010	20201832	0	Cytotoxicity and apoptosis by survivin small interfering RNA in bladder cancer cells	survivin,Caspase-3	
Lee EJ, Kim WJ	2010	20564512	0	Cordycepin suppresses TNF-alpha-induced invasion, migration and matrix metalloproteinase-9 expression in human bladder cancer cells	MMP-9,MMP-2,NF-KB,AP-1	
Li Q, Wang XH	2010	20646592	1	[Induction of cell cycle arrest in bladder cancer RT4 cells by capsaicin]	TRPV1,P53, P21, CDK2	
Li Y, Yang K, M	2010	19669587	0	Inhibition of TGF-beta receptor I by siRNA suppresses the motility and invasiveness of T24 bladder cancer cells via modulation of integrins and matrix metalloproteinase	TGFBRI,MMP2,MMP9	TGF-beta
Li Y, Yang X, S	2010	20811684	1	VEGFR and EGFR inhibition increases epithelial cellular characteristics and chemotherapy sensitivity in mesenchymal bladder cancer cells	EGFR, VEGR, E-cadherin，vimentin	EMT
Liu C, Wu X, Lı	2010	20525250	0	Antisense oligonucleotide targeting Livin induces apoptosis of human bladder cancer cell via a mechanism involving caspase 3	caspase3,Livin	
Lou GG, Yao H	2010	20503476	0	Brucea javanica oil induces apoptosis in T24 bladder cancer cells via upregulation of caspase-3, caspase-9, and inhibition of NF-kappaB and COX-2 expressions	caspase-3, capase-9, NF-kappaB p65,COX-2	caspase
Lu Q, Lu C, Zh	2010	19767219	1	MicroRNA-221 silencing predisposed human bladder cancer cells to undergo apoptosis induced by TRAIL	p27,caspase 3	
Ma Y, Yu WD,	2010	20564622	0	1,25D3 enhances antitumor activity of gemcitabine and cisplatin in human bladder cancer models	p73,caspases 8, caspases9, caspases 3,caspases 10,VDR,PARP,	
Makridakis M, I	2010	20423150	0	Analysis of secreted proteins for the study of bladder cancer cell aggressiveness	SPARC	
Mao QQ, Bai Y	2010	20521268	0	Resveratrol confers resistance against taxol via induction of cell cycle arrest in human cancer cell lines	Akt,JNK,Erk,p38,Bcl-2,CDK1,CDK2,cyclin A, cyclin B1, cyclin D1, cyclin E, histone H3,Bid,PARP,Bcl-xl, Bax, p-IkB-a,NF-kB	cell cycle
Mechlin CW, Ta	2010	20488474	1	Gli2 expression and human bladder transitional carcinoma cell invasiveness	Gli2	

Table S1. The list of bladder cancer related articles either used or not used RT4.

Author	Year	PMID	using RT4 or not(0 represents No;1 represents Yes)	Title	Gene	Pathway
Metalli D, Lova	2010	20395438	0	The insulin-like growth factor receptor I promotes motility and invasion of bladder cancer cells through Akt- and mitogen-activated protein kinase-dependent activation of paxillin	IGF-IR,Akt,ERK,S6K,paxillin,P-S126,P-S178	AKT,MAPK
Mizutani Y, Kat	2010	20596678	0	Prognostic significance of second mitochondria-derived activator of caspase (Smac/DIABLO) expression in bladder cancer and target for therapy	Smac/DIABLO	
Morikawa T, M	2010	21166702	0	Ribonucleotide reductase M2 subunit is a novel diagnostic marker and a potential therapeutic target in bladder cancer	RRM2	
Ostenfeld MS, I	2010	19915607	1	miR-145 induces caspase-dependent and -independent cell death in urothelial cancer cell lines with targeting of an expression signature present in Ta bladder tumors	Bcl-2,caspase3,caspase7	
Ou L, Guo Y, L	2010	20620593	0	RNA interference suppressing PLCE1 gene expression decreases invasive power of human bladder cancer T24 cell line	PLCE1,BCL2,MMP2,MMP9	Ras
Zhang, Z. G et a	2010	21211316	0	Growth inhibition and mechanisms of human bladder cancer T24 cells by adenovirus-mediated ING4 gene in vitro	ING4,Bcl-2,BAX,p53,HIF-1 α,Caspase-3	
Zhang, J et al.	2010	20845808	0	Glycosyl-phosphatidylinositol-anchored interleukin-2 expressed on tumor-derived exosomes induces antitumor immune response in vitro	GPI-IL-2,ICAM-1,HSP70,MAGE-1	
Yang, S et al.	2010	20593288	0	Expression and clinical significance of hepaCAM and VEGF in urothelial carcinoma	hepaCAM,VEGF,	
Yang, D et al.	2010	20119625	0	Therapeutic potential of siRNA-mediated combined knockdown of the IAP genes (Livin, XIAP, and Survivin) on human bladder cancer T24 cells	Livin,XIAP,Survivin,caspase-3,caspase-7,caspase-9	
Yang, D et al.	2010	20460713	0	Suppression of livin gene expression by siRNA leads to growth inhibition and apoptosis induction in human bladder cancer T24 cells	Livin,caspase-3,caspase-7,caspase-9	
Yamada, T et al	2010	20546877	1	TRPV2 activation induces apoptotic cell death in human T24 bladder cancer cells: a potential therapeutic target for bladder cancer	TRPV1,TRPV2,TRPV3,TRPV4,	
Xue, Y et al.	2010	21042751	0	Telomerase suppression initiates PML-dependent p53 activation to inhibit bladder cancer cell growth	hTERT,PML,P53,P21,	
Xie, P et al.	2010	21223810	0	Detection, verification and significance of differentially expressed miRNAs in bladder urothelial carcinoma	miR-29b-1,miR-300,miR-923	
Wu, Z et al.	2010	21170262	0	RalBP1 is necessary for metastasis of human cancer cell lines	RalA, RalB, RalBP1,RalB-4, Sec5,PLD1,Rac1,	
Wu, J. T et al.	2010	20083299	0	Androgen receptor is a potential therapeutic target for bladder cancer	AR,CCND1,Bcl-XL,MMP-9,	
Wang, Y. B et a	2010	19748259	0	Diallyl trisulfide induces Bcl-2 and caspase-3-dependent apoptosis via downregulation of Akt phosphorylation in human T24 bladder cancer cells	PARP,Caspase-3,PDK1,AKT,BCL-2,BAX,	PI3K-Akt pathway
Wang, H et al.	2010	20940393	0	Phosphorylation of RalB is important for bladder cancer cell growth and metastasis	Ralb,	

Table S1. The list of bladder cancer related articles either used or not used RT4.

Author	Year	PMID	using RT4 or not(0 represents No;1 represents Yes)	Title	Gene	Pathway
Tseng-Rogensk	2010	20093479	1	Loss of 15-hydroxyprostaglandin dehydrogenase expression contributes to bladder cancer progression	PGDH,	
Toki, K et al.	2010	21042705	0	CpG hypermethylation of cellular retinol-binding protein 1 contributes to cell proliferation and migration in bladder cancer	CRBP1,	
Tanaka, N et al.	2010	20978160	0	Cis-dichlorodiammineplatinum upregulates angiotensin II type 1 receptors through reactive oxygen species generation and enhances VEGF production in bladder cancer	AT1R,VEGF	
Stella, J et al.	2010	19372055	1	Differential ectonucleotidase expression in human bladder cancer cell lines	NTPDase	
Shiota, M et al.	2010	21138866	0	Foxo3a suppression of urothelial cancer invasiveness through Twist1, Y-box-binding protein 1, and E-cadherin regulation	Foxo3a,TWIST1,YB-1,E-cadherin,p300	
Shimada, K et a	2010	19860843	0	Role of syndecan-1 (CD138) in cell survival of human urothelial carcinoma	CD138,FLIP,JUNB,Caspase-3,Caspase-8,p17,p43	
Shan, Y et al.	2010	20204301	0	p38 MAPK plays a distinct role in sulforaphane-induced up-regulation of ARE-dependent enzymes and down-regulation of COX-2 in human bladder cancer cells	p38 MAPK,GSTA1-1,TR-1,COX-2,NRF2,	
Shahjee, H. M e	2010	21143984	0	Antiproliferative factor decreases Akt phosphorylation and alters gene expression via CKAP4 in T24 bladder carcinoma cells	CKAP4,AKT,P53,GSK3B,B-catenin,MMP2	
See, W. A et al.	2010	19450997	0	p21 Expression by human urothelial carcinoma cells modulates the phenotypic response to BCG	P21	
Rose, A et al.	2010	20649572	1	Stimulatory effects of the multi-kinase inhibitor sorafenib on human bladder cancer cells	Raf,ERK1/2,PARP	
Qu, W et al.	2010	19181544	0	Experimental study on inhibitory effects of histone deacetylase inhibitor MS-275 and TSA on bladder cancer cells	p21,CCNA,CCNE,MS-275,bcl-2,bax	
Plissonnier, M.	2010	20099277	1	Insights on distinct pathways of thiazolidinediones (PPARgamma ligand)-promoted apoptosis in TRAIL-sensitive or -resistant malignant urothelial cells	PPAR γ,TRAIL,P53,P21,P27,CCNB,Caspase-3,Caspase-8,Caspase-9,BCL-2,BAX,BID,FABP,DR4,DR5,FLIP	
Peuhu, E et al.	2010	20920317	0	Molecular targets for the protodynamic action of cis-urocanic acid in human bladder carcinoma cells	ERK1/2,JNK	ERK and JNK signalling pathways
Pan, C et al.	2010	20628239	0	Exon 2 methylation inhibits hepaCAM expression in transitional cell carcinoma of the bladder	hepaCAM	
Ozawa, A et al.	2010	19681894	0	Inhibition of bladder tumour growth by histone deacetylase inhibitor	P21,HDAC1	
Ou, L et al.	2010	20620593	0	RNA interference suppressing PLCE1 gene expression decreases invasive power of human bladder cancer T24 cell line	PLCE1,BCL2,MMP-2,MMP-9,	
Zhu, Z et al	2009	19224167	0	Expression of transcription factor Oct4 in bladder cancer cell line T24 and its effects on the biological characteristics of the cells	43742	
Zhu Y et.al	2009	20095141	0	MONCPT exerts anti-cancer activities via inducing G2/M arrest and apoptosis in human bladder cancer.	CDK7, p-Cdc2,cyclinB1,p27,p21,PARP	
Zheng L et.al	2009	19513619	1	Selection of optimal antisense accessible sites of uroplakin II mRNA for bladder urothelium	Up II,	

Table S1. The list of bladder cancer related articles either used or not used RT4.

Author	Year	PMID	using RT4 or not(0 represents No;1 represents Yes)	Title	Gene	Pathway
Zhang S et.al	2009	19399408	0	Effects on biological behavior of bladder carcinoma T24 cells via silencing DNMT1 and/or DNMT3b with shRNA in vitro	DNMT1,DNMT3b,	
Zhang JM et.al	2009	20021824	0	Exosomes derived form bladder transitional cell carcinoma cells induce CTL cytotoxicity in vitro	HSP70、ICAM-1、CK20	
Yeh HH et.al	2009	19182994	0	Ha-ras oncogene-induced Stat3 phosphorylation enhances oncogenicity of the cell	Ha-ras, IL-6,Stat3	
Xu NR et.al	2009	19778817	0	Reversion transcriptional expression of DAPK in bladder cancer T24 cells 5-aza-2'-deoxycytidine	DAPK	
White-Gilbertso	2009	19625063	0	Oxidative stress sensitizes bladder cancer cells to TRAIL mediated apoptosis by down-regulating anti-apoptotic proteins.	TRAIL,caspase-3/7,EF2,FLIPS, XIAP, survivin.	
Wang Y et.al	2009	19322013	0	Protein kinase C is involved in arsenic trioxide-induced apoptosis and inhibition of proliferation in human bladder cancer cells	ATO,PKC,caspase-3	
Tully BT et.al	2009	19573899	1	Defects in muscarinic receptor cell signaling in bladder urothelial cancer cell lines	m1,m2,m3,m4,m5	
Tomlinson et.al	2009	19458078	1	Fibroblast growth factor receptor 1 promotes proliferation and survival via activation of the mitogen-activated protein kinase pathway in bladder cancer	FGFR1,FGFR2,FGFR3,FRS2α， PLC γ，cyclin D1、MCL1、phospho-BAD	
Tang et.al	2009	19174556	0	WIF1, a Wnt pathway inhibitor, regulates SKP2 and c-myc expression leading to G1 arrest and growth inhibition of human invasive urinary bladder cancer cells	WIF1,SKP2,c-myc,p21,p27,B-catenin,	
Szliszka et.al	2009	20430723	0	TRAIL-induced apoptosis and expression of death receptor TRAIL-R1 and TRAIL-R2 in bladder cancer cells	TRAIL,TRAIL- r1,TRAIL- r2	
Szanto et.al	2009	19331146	0	Critical role of bad phosphorylation by Akt in cytostatic resistance of human bladder cancer cells	caspase-3,Akt,Bad,GSK,PI3K	
Sonpavde et.al	2009	18534874	0	Sunitinib malate is active against human urothelial carcinoma and enhances the activity of cisplatin in a preclinical mode	VEGFR2,Kit,caspase-3	
Shan et.al	2009	19287971	0	Sulforaphane down-regulates COX-2 expression by activating p38 and inhibiting NF-kappaB-DNA-binding activity in human bladder T24 cells	COX-2,NF-kappaB,P38	
See et.al	2009	19154459	0	Bacille-Calmette Guerin induces caspase-independent cell death in urothelial carcinoma cells together with release of the necrosis-associated chemokine high molecular group box protein 1	HMGB1,NF-kappaB,TLR-2,TLR-4	
Qin et.al	2009	18617357	0	In vitro and in vivo inhibitory effect evaluation of cyclooxygenase-2 inhibitors, antisense cyclooxygenase-2 cDNA, and their combination on the growth of human bladder cancer cells.	COX-2,	
Qian et.al	2009	18825409	0	Regulation of TLR4-induced IL-6 response in bladder cancer cells by opposing actions of MAPK and PI3K signaling	TLR4,IL-6,CD14,ERK,P38,JUK,Akt	
Philips et.al	2009	19729851	1	Induction of apoptosis in human bladder cancer cells by green tea catechins	Tmepai,Wnt2,Ccl20,IL-8,Pecam1,Wisp1	
Pan et.al	2009	19624598	0	Cell adhesion to fibronectin induces mitomycin C resistance in bladder cancer cells	caspase-8,caspase-9,GSK-3beta, PI3-K,Akt	

Table S1. The list of bladder cancer related articles either used or not used RT4.

Author	Year	PMID	using RT4 or not(0 represents No;1 represents Yes)	Title	Gene	Pathway
Ning et.al	2009	19589244	0	Subpopulations of stem-like cells in side population cells from the human bladder transitional cell cancer cell line T24	ABCG2	
Nikpour et.al	2009	19706044	1	Differential effects of Nucleostemin suppression on cell cycle arrest and apoptosis in the bladder cancer cell lines 5637 and SW1710	NS,RB1,p16,p53	
Nakanishi et.al	2009	19641368	0	Effect of vascular endothelial growth factor and its receptor inhibitor on proliferation and invasion in bladder cancer	VEGF,VEGFR1,VEGFR2	
Muramaki et.al	2009	19007378	0	Chemosensitization of gemcitabine-resistant human bladder cancer cell line both in vitro and in vivo using antisense oligonucleotide targeting the anti-apoptotic gene, clusterin	scl -2	
Miyake et.al	2009	19320847	0	siRNA-mediated knockdown of the heme synthesis and degradation pathways: modulation of treatment effect of 5-aminolevulinic acid-based photodynamic therapy in urothelial cancer cell lines	HO-1,FECH	
Matsui et.al	2009	19625496	0	Intravesical combination treatment with antisense oligonucleotides targeting heat shock protein-27 and HTI-286 as a novel strategy for high-grade bladder cancer	Hsp27,Akt,MDR1,p38,Bcl-2,Bax,PARP,caspase-9	
Mariotti et.al	2009	18835089	0	Inhibition of T24 human bladder carcinoma cell migration by RNA interference suppressing the expression of HD-PTP	HD-PTP,FAK	
Makridakis et.al	2009	19105184	0	Chromosomal and proteome analysis of a new T24-based cell line model for aggressive bladder cancer	CATD	
Lovat et.al	2009	19237611	0	Proepithelin is an autocrine growth factor for bladder cancer	Proepithelin,Akt,ERK	
Liu et.al	2009	19214503	0	Recombinant bacillus Calmette-Guerin (BCG) expressing interferon-alpha 2B enhances human mononuclear cell cytotoxicity against bladder cancer cell lines in vitro	IFN-alpha,IFN-gamma,IL-2	
Liu et.al	2009	18555709	0	Livin may serve as a marker for prognosis of bladder cancer relapse and a target of bladder cancer treatment	Livinalpha,Livinbeta,Caspase 3	
Lin et.al	2009	19157460	0	MicroRNA-143 as a tumor suppressor for bladder cancer	miRNA-143,K-RAS,H-RAS	miRNA-143-RAS
Li et.al	2009	19955836	0	Menthol induces cell death via the TRPM8 channel in the human bladder cancer cell line T24	TRPM8,	
Lee et.al	2009	19733546	0	Cordycepin causes p21WAF1-mediated G2/M cell-cycle arrest by regulating c-Jun N-terminal kinase activation in human bladder cancer cells	cyclin B1, pCdc2, Cdc2, pCdc25c, Cdc25c, p21WAF1, p27, p53	
Lee et.al	2009	19048611	0	Inhibitory effects of the aqueous extract of Magnolia officinalis on the responses of human urinary bladder cancer 5637 cells in vitro and mouse urinary bladder tumors induced by N-Butyl-N-(4-hydroxybutyl) nitrosamine in vivo	Bax, Bcl-2, cytochrome c, caspase 3, MMP-2, MMP-9	
Konstantakou e	2009	19578756	1	Human bladder cancer cells undergo cisplatin-induced apoptosis that is associated with p53-dependent and p53-independent responses	p53,Caspase-8,Caspase-9,Caspase-3,PARP,Bik、Bim、Bcl-2、FAP-1、Fas、FasL、TRAIL、Puma、Caspase-10、ATP7A、ATP7B、MRP1	

Table S1. The list of bladder cancer related articles either used or not used RT4.

Author	Year	PMID	using RT4 or not(0 represents No;1 represents Yes)	Title	Gene	Pathway
Kim et.al	2009	19415719	0	Cyclin D1b variant promotes cell invasiveness independent of binding to CDK4 in human bladder cancer cells	cyclin D1a、cyclin D1b,CDK4	
Khin et.al	2009	19326429	0	BAMBI gene is epigenetically silenced in subset of high-grade bladder cancer	TGF-beta,BMP,BAMBI	
Kanda et.al	2009	18726116	0	Loss of PTEN function may account for reduced proliferation pathway sensitivity to LY294002 in human prostate and bladder cancer cells	PI3-K,PTEN,Akt,ERK	
Isono et.al	2009	19578758	0	Suppression of cell invasiveness by periostin via TAB1/TAK1	TAB1,TAK1	TAB1-TAK1
Higuchi et.al	2009	19139883	0	A possible mechanism of intravesical BCG therapy for human bladder carcinoma: involvement of innate effector cells for the inhibition of tumor growth	CD1d	
Gee et.al	2009	19846907	1	Cyclin-mediated G1 arrest by celecoxib differs in low-versus high-grade bladder cancer	COX-2,Cyclin D1,Cyclin B1, Rb	
Fukushima et.al	2009	19934319	0	Loss of DeltaNp63alpha promotes invasion of urothelial carcinomas via N-cadherin/Src homology and collagen/extracellular signal-regulated kinase pathway	ΔNp63α,N-cadherin,E-cadherin,p63,ERK,Akt,MMP-9,0	
Flaig et.al	2009	19220256	1	Dual epidermal growth factor receptor and vascular endothelial growth factor receptor inhibition with vandetanib sensitizes bladder cancer cells to cisplatin in a dose- and sequence-dependent manner	p27,p21	
Fechner et.al	2009	19081609	1	Rapamycin inhibits in vitro growth and release of angiogenetic factors in human bladder cancer	VEGF,mTOR	
Dyrskjøt et.al	2009	19487295	1	Genomic profiling of microRNAs in bladder cancer: miR-129 is associated with poor outcome and promotes cell death in vitro	miR-145,miR-129, miR-133b, miR-518c, miR-21, GALNT1 , SOX4	miR-145 - GALNT1 / SOX4
Dehnavi et.al	2009	19219652	0	The effect of TGF-beta2 on MMP-2 production and activity in highly metastatic human bladder carcinoma cell line 5637	TGF-beta,MMP-2	
Dasgupta et.al	2009	19569044	0	Forced cytochrome B gene mutation expression induces mitochondrial proliferation and prevents apoptosis in human uroepithelial SV-HUC-1 cells	CYTB,COX-I,Bax,PARP,Lamin B1	
Colquhoun et.al	2009	19237174	0	Mechanisms of action of eicosapentaenoic acid in bladder cancer cells in vitro: alterations in mitochondrial metabolism, reactive oxygen species generation and apoptosis induction	caspase-3	
Chen et.al	2009	19853870	0	MB49 murine urothelial carcinoma: molecular and phenotypic comparison to human cell lines as a model of the direct tumor response to bacillus Calmette-Guerin	NF-kappaB, AP1 , C/EBP, IL-6, p21	
Boorjian et.al	2009	18845648	0	Expression and significance of androgen receptor coactivators in urothelial carcinoma of the bladder	AR, NCOA1、NCOA2、NCOA3、CREBBP,EP300	
Boiteux et.al	2009	19115207	1	A-FABP, a candidate progression marker of human transitional cell carcinoma of the bladder, is differentially regulated by PPAR in urothelial cancer cells	A-FABP,PPAR,	
Beecken et.al	2009	19148551	1	Expression of angiogenesis inhibitors in human bladder cancer may explain rapid metastatic progression after radical cystectomy	angiostatin, endostatin , thrombospondin-1.	

Table S1. The list of bladder cancer related articles either used or not used RT4.

Author	Year	PMID	using RT4 or not(0 represents No;1 represents Yes)	Title	Gene	Pathway
Amantini et.al	2009	19502594	1	Triggering of transient receptor potential vanilloid type 1 (TRPV1) by capsaicin induces Fas/CD95-mediated apoptosis of urothelial cancer cells in an ATM-dependent manner	TRPV1,Fas/CD95, Bcl-2 , caspase families ,ATM,CHK2,p53	
Adam et.al	2009	19671845	0	miR-200 expression regulates epithelial-to-mesenchymal transition in bladder cancer cells and reverses resistance to epidermal growth factor receptor therapy	miR-200b, miR-200c,EGFR, E-cadherin，ZEB1、ZEB2、ERRFI-1,MAPK,	miR-200 - ERRFI-1 -EGFR
Abedin et.al	2009	19126649	1	Elevated NCOR1 disrupts a network of dietary-sensing nuclear receptors in bladder cancer cells	NCOR1,PPAR,VDR,PPARgamma、Farnesoid x	
Zhao et.al	2008	19007019	0	[Proliferation apoptotic influence of crocin on human bladder cancer T24 cell line]	Bcl-2、Bax、Survivin、Cyclin D1	
Yang et.al	2008	18358601	0	Up-regulation of p21WAF1/Cip1 by saRNA induces G1-phase arrest and apoptosis in T24 human bladder cancer cells	p21,Bcl-xL,caspase-3,PARP	
Wu et.al	2008	17705801	0	RNA-interference-mediated Cdc42 silencing down-regulates phosphorylation of STAT3 and suppresses growth in human bladder-cancer cells	Cdc42,STAT3	
Wang et.al	2008	18334113	0	[Effects of survivin small interfering RNA on biological behaviors of bladder cancer T24 cells]	Survivin,	
Wang et.al	2008	18563329	0	Synthetic Smac peptide enhances chemo-sensitivity of bladder cancer cells	XIAP, Caspase-3	
Wang et.al	2008	18826151	0	[Screening of anti-tumor parts from the seeds of Livistona chinensis and its anti-angiogenesis effect]	VEGF, Flk-1,	
Wang et.al	2008	18671724	0	Norsolorinic acid inhibits proliferation of T24 human bladder cancer cells by arresting the cell cycle at the G0/G1 phase and inducing a Fas/membrane-bound Fas ligand-mediated apoptotic pathway	p53, p21, Fas receptor, FasL and caspase 8	
Tseng et.al	2008	18308117	1	Loss of 15-hydroxyprostaglandin dehydrogenase expression disrupts urothelial differentiation	PGDH,E-cadherin	
Tian et.al	2008	18342436	0	Effects of curcumin on bladder cancer cells and development of urothelial tumors in a rat bladder carcinogenesis model	Bcl-2,Survivin,Bax,p53	
Teng et.al	2008	18310301	0	Roles of estrogen receptor alpha and beta in modulating urothelial cell proliferation	Eralpha, Erbeta, MEK, cyclin D1,cyclin E	
Tang et.al	2008	19138991	1	Effects of the kava chalcone flavokawain A differ in bladder cancer cells with wild-type versus mutant p53	p53,p21,p27,SKP2,CDK1	
Szarvas et.al	2008	19088043	1	Angiogenic switch of angiopietins-Tie2 system and its prognostic value in bladder cancer	VEGF,Ang-1 ,Ang-2,Tie2 ,	
Stohr et.al	2008	18593932	0	ATM mediates cytotoxicity of a mutant telomerase RNA in human cancer cells	ATM,p53,hTER,	
Singh et.al	2008	18172282	1	Oral silibinin inhibits in vivo human bladder tumor xenograft growth involving down-regulation of survivin	survivin,caspase-3,p53,	
Shi et.al	2008	18499162	0	Association of low expression of notch-1 and jagged-1 in human papillary bladder cancer and shorter survival	Notch-1,Notch-2,Notch-3, Jagged-1 , Delta-like-1	

Table S1. The list of bladder cancer related articles either used or not used RT4.

Author	Year	PMID	using RT4 or not(0 represents No;1 represents Yes)	Title	Gene	Pathway
She et.al	2008	18787416	0	Identification of side population cells from bladder cancer cells by DyeCycle Violet staining	ABCG2、MDRI、Bmi-1、Oct-4	
Pan et.al	2008	19080691	0	[Effects of double targeting gene therapy by suicide gene combined with endostatin gene in treatment of bladder cancer: an experimental study]	ES,TIE	
Okamoto et.al	2008	18242386	0	Etodolac, a selective cyclooxygenase-2 inhibitor, induces upregulation of E-cadherin and has antitumor effect on human bladder cancer cells in vitro and in vivo	COX-2,E-cadherin,	
Moussa et.al	2008	17603559	0	Inhibition of thromboxane synthase activity modulates bladder cancer cell responses to chemotherapeutic agents	TXAS,caspase-3,survivin,	
Mao et.al	2008	18725195	0	Up-regulation of E-cadherin by small activating RNA inhibits cell invasion and migration in 5637 human bladder cancer cells	p21,E-cadherin,b-catenin,MMP7,cyclin D1	
Mandeville et.al	2008	18990147	1	P-cadherin as a prognostic indicator and a modulator of migratory behaviour in bladder carcinoma cells	P-cadherin,β-catenin,	
Lv et.al	2008	18837328	0	[Influence of crocin on gene expression profile of human bladder cancer cell lines T24]	p21,cyclinD1,	
Liu et.al	2008	18756993	0	[Effects of gene Livin transfection affect on the apoptosis in bladder carcinoma cells]	Livin,	
Liu et.al	2008	18241249	0	Tumour growth inhibition by an imidazoquinoline is associated with c-Myc down-regulation in urothelial cell carcinoma	TLR7,c-Myc,MyD88,NF-kappaB,cyclin D2 , CCND2 ,CDK4,Bcl-2	
Lee et.al	2008	18801463	0	Signaling pathway for TNF-alpha-induced MMP-9 expression: mediation through p38 MAP kinase, and inhibition by anti-cancer molecule magnolol in human urinary bladder cancer 5637 cells	MMP-2,MMP-9,TNF-alpha,NF-kappaB,p38	
Lee et.al	2008	18468578	0	Magnolol elicits activation of the extracellular signal-regulated kinase pathway by inducing p27KIP1-mediated G2/M-phase cell cycle arrest in human urinary bladder cancer 5637 cells	CDKs,ERK、p38 ,MAPK, JNK,ERK,RAS,Raf,p27,cyclin D1, cyclin E, cyclin B1, CDK2 CDK4, pCdc2, Cdc2, pCdc25c, and Cdc25c	
Lampidonis et.a	2008	18824087	0	Cloning and functional characterization of the 5' regulatory region of ovine Hormone Sensitive Lipase (HSL) gene	HSL,	
Kuo et.al	2008	18381677	0	The grape and wine constituent piceatannol inhibits proliferation of human bladder cancer cells via blocking cell cycle progression and inducing Fas/membrane bound Fas ligand-mediated apoptotic pathway	p21,Fas,p53, caspase-8	
Kunze er.al	2008	18425331	0	Antisense-mediated inhibition of survivin, hTERT and VEGF in bladder cancer cells in vitro and in vivo	survivin,hTERT,VEGF,	
Kobayashi et.al	2008	19122685	0	Mechanistic analysis of resistance to REIC/Dkk-3-induced apoptosis in human bladder cancer cells	Bcl-2,Ad-REIC	
Kim et.al	2008	18296682	0	Requirement for Ras/Raf/ERK pathway in naringin-induced G1-cell-cycle arrest via p21WAF1 expression	p21WAF1,p53,JNK,p38,ERK,Ras,Raf, cyclin D1,CDK4 , cyclin E,CDK2	

Table S1. The list of bladder cancer related articles either used or not used RT4.

Author	Year	PMID	using RT4 or not(0 represents No;1 represents Yes)	Title	Gene	Pathway
Kawanishi et.al	2008	18451219	0	Secreted CXCL1 is a potential mediator and marker of the tumor invasion of bladder cancer	CXCL1,CXCL2,MMP-13,	
Herbsleb et.al	2008	18647386	0	Increased cell motility and invasion upon knockdown of lipolysis stimulated lipoprotein receptor (LSR) in SW780 bladder cancer cells	LSR,p53,	
He et.al	2008	18955789	0	Impaired delta NP63 expression is associated with poor tumor development in transitional cell carcinoma of the bladder	delta Np63,cyclin D1,	
Hatina et.al	2008	18398596	0	[Bladder carcinoma cell lines as models of the pathobiology of bladder cancer. Review of the literature and establishment of a new progression series]	E-Cadherin	
Gallagher et.al	2008	18980977	1	Recurrence of urothelial carcinoma of the bladder: a role for insulin-like growth factor-II loss of imprinting and cytoplasmic E-cadherin immunolocalization	E-cadherin,IGF-II	
Gabriel et.al	2008	19020723	0	Evaluation of cytotoxic effects induced by bcl-2 and bcl-xL antisense-oligodeoxynucleotides in normal urothelium and transitional cell carcinoma	Bcl-2	
Dhawan et.al	2008	18413803	0	Cyclooxygenase-2 dependent and independent antitumor effects induced by celecoxib in urinary bladder cancer cells	COX-2,pRB	
Chen et.al	2008	18347154	0	Antitumor effect of dsRNA-induced p21(WAF1/CIP1) gene activation in human bladder cancer cells	p21,	
Chen et.al	2008	19102933	0	[Inhibition of bladder cancer cell growth and angiogenesis by co-blockage of vascular endothelial growth factor and its receptor KDR]	VEGF,KDR,Topo II,	
Chen et.al	2008	18448488	0	Rapid activation of Stat3 and ERK1/2 by nicotine modulates cell proliferation in human bladder cancer cells	Stat3,ERK1/2,Cyclin D1,Cyclin A,Bcl-2,	
Buytaert et.al	2008	17952126	1	Molecular effectors and modulators of hypericin-mediated cell death in bladder cancer cells	MMP1, MMP10, MMP13, AKR1C1, HO-1,COX-2, AKR1C1, CHOP,Bcl-2,p38	
Chaffer, C. L.	2007	17697126	0	Aberrant fibroblast growth factor receptor signaling in bladder and other cancers	FGF、FGFR	MET
Jackson, P.	2007	17982617	0	An alternatively spliced KAI1 mRNA is expressed at low levels in human bladder cancers and bladder cancer cell lines and is not associated with invasive behaviour	KAI1	
Smith, E. B.	2007	17509356	0	Antitumor effects of imidazoquinolines in urothelial cell carcinoma of the bladder	TLR,IL-6,TNF-a	
Peng, C. C.	2007	16930895	1	Antrodia camphorata extract induces replicative senescence in superficial TCC, and inhibits the absolute migration capability in invasive bladder carcinoma cells	P-53,P-21,Igg,ppRB,Cdc2\cyclin-b1,	
Lee, S. J.	2007	17671727	0	Aqueous extract of Magnolia officinalis mediates proliferative capacity, p21WAF1 expression and TNF-alpha-induced NF-kappaB activity in human urinary bladder cancer 5637 cells; involvement of p38 MAP kinase	p38 MAP\p21WAF1\NF-κB\CDK2\CDK4\p21\p27\p53\SB203580	

Table S1. The list of bladder cancer related articles either used or not used RT4.

Author	Year	PMID	using RT4 or not(0 represents No;1 represents Yes)	Title	Gene	Pathway
Chai, C. Y.	2007	17906315	0	Arsenic salt-induced DNA damage and expression of mutant p53 and COX-2 proteins in SV-40 immortalized human uroepithelial cells	NHEKs\COX-2 \	
Chai, C. Y.	2007	17683884	0	Arsenic salts induced autophagic cell death and hypermethylation of DAPK promoter in SV-40 immortalized human uroepithelial cells	Beclin-1\DAPK\	
Zhang, G.	2007	17483019	0	Bacillus Calmette-Guerin induces p21 expression in human transitional carcinoma cell lines via an immediate early, p53 independent pathway	cyclin D1, p27, and p21,p53	
Chen, F.	2007	17870116	0	Bacillus Calmette-Guerin inhibits apoptosis in human urothelial carcinoma cell lines in response to cytotoxic injury	NF kB, AP1 and NF kB-AP1	
Wang, P.	2007	17445681	0	Bladder cancer cell invasion is enhanced by cross-talk with fibroblasts through hepatocyte growth factor	HGF,IL-1b, PDGF-Ab, basic FGF	
Sandes, E.	2007	17982689	0	Cathepsin B is involved in the apoptosis intrinsic pathway induced by Bacillus Calmette-Guerin in transitional cancer cell lines	pro-caspase 9,BID,Bax/Bak	caspase 8,caspase 9
Shen, K. H.	2007	17845507	0	Chalcone arrests cell cycle progression and induces apoptosis through induction of mitochondrial pathway and inhibition of nuclear factor kappa B signalling in human bladder cancer cells	cyclin B1, cyclin A and Cdc2,Bax and Bak,Bcl-2 and Bcl-X,caspase-9and caspase-3,p21, p27,	
Qin, J.	2007	17266926	0	A component of green tea, (-)-epigallocatechin-3-gallate, promotes apoptosis in T24 human bladder cancer cells via modulation of the PI3K/Akt pathway and Bcl-2 family proteins	Bcl-2/PARP/caspase-3	PI3K/Akt
Wang, Y.	2007	17453818	0	Downregulation of missing in metastasis gene (MIM) is associated with the progression of bladder transitional carcinomas	MIM	
Zhang, J.	2007	17430655	0	Effects of selective cyclooxygenase-2 inhibitor on proliferation and apoptosis of human bladder cancer cell line T24]	COX-2/ Bcl-2 and Bax	
Sonpavde, G.	2007	17572228	0	Efficacy of selective estrogen receptor modulators in nude mice bearing human transitional cell carcinoma	SERMs	
Wang, L.	2007	17641843	0	Expression of X-linked inhibitor of apoptosis protein and its effect on chemotherapeutic sensitivity of bladder carcinoma	XIAP	
Gazzaniga, P.	2007	17159604	0	Gemcitabine-induced apoptosis in 5637 cell line: an in-vitro model for high-risk superficial bladder cancer	caspase-3, -8 and -9	bcl-2, bcl-X, bax, survivin and fas
Baniasadi, S.	2007	17489360	0	Gene expression profiles in T24 human bladder carcinoma cells by inhibiting an L-type amino acid transporter, LAT1	LAT1	
Chakraborty, A.	2007	17572226	0	Granulocyte colony-stimulating factor/granulocyte colony-stimulating factor receptor biological axis promotes survival and growth of bladder cancer cells	G-CSF	G-CSF/G-CSFR-mediated STAT3
Nutt, J. E.	2007	17311025	0	hEGR1 is induced by EGF, inhibited by gefitinib in bladder cell lines and related to EGF receptor levels in bladder tumours	hEGR1	
Buckley, M. T.	2007	17935615	0	The histone deacetylase inhibitor belinostat (PXD101) suppresses bladder cancer cell growth in vitro and in vivo	PXD101	p21WAF1,

Table S1. The list of bladder cancer related articles either used or not used RT4.

Author	Year	PMID	using RT4 or not(0 represents No;1 represents Yes)	Title	Gene	Pathway
Yeh CC et al.	2007	18210748	0	Shikonin-induced apoptosis involves caspase-3 activity in a human bladder cancer cell line (T24)	caspase-9、caspase-3、cyclin A 、cyclin D 、cyclin E、CDK2、 CDK4 、 CDK6、 P21	
Aaltonen V et a	2007	17320279	0	PKC inhibitor Go6976 induces mitosis and enhances doxorubicin-paclitaxel cytotoxicity in urinary bladder carcinoma cells	CDC2、CDC25C、CHK1、CHK2、	
Wu Y et al.	2007	16878152	0	Neuromedin U is regulated by the metastasis suppressor RhoGDI2 and is a novel promoter of tumor formation, lung metastasis and cancer cachexia	RhoGDI2、NMU	
Challita-Eid PM	2007	17575147	0	Monoclonal antibodies to six-transmembrane epithelial antigen of the prostate-1 inhibit intercellular communication in vitro and growth of human tumor xenografts in vivo	STEAP-1	
Terao S et al.	2007	17919690	0	Midkine promoter-based conditionally replicative adenovirus for targeting midkine-expressing human bladder cancer model	MK	
Ornskov D et a	2007	17274952	1	Insulin induces a transcriptional activation of epiregulin, HB-EGF and amphiregulin, by a PI3K-dependent mechanism: identification of a specific insulin-responsive promoter element	HER1、HB - EGF、AR、EPI 、PI3K	
Munk M et al.	2007	17092291	1	Inhibition of the epidermal growth factor receptor in bladder cancer cells treated with the DNA-damaging drug etoposide markedly increases apoptosis	EGFR	
Gao XD et al.	2007	17531113	0	Inhibition of telomerase with human telomerase reverse transcriptase antisense enhances tumor necrosis factor-alpha-induced apoptosis in bladder cancer cells	hTERT	
Shimada O et al	2007	17320696	0	Human agonistic antibody to tumor necrosis factor-related apoptosis-inducing ligand receptor 2 induces cytotoxicity and apoptosis in prostate cancer and bladder cancer cells	TRAIL-R1 、TRAIL-R2、HGS-ETR2 、Caspase-3, Caspase-6, Caspase-9	
Abraham S et al	2006	16428472	1	Expression of EphA2 and Ephrin A-1 in carcinoma of the urinary bladder	EphA2.Epherin A-1,E-cadherin,EGFR,Her2	
Baierlein SA et a	2006	16896593	0	Combined effect of tumor necrosis factor-alpha and ionizing radiation on the induction of apoptosis in 5637 bladder carcinoma cells	TGF-α,CD95,TRAIL,caspase-9,APAF-9	
Chaffer CL et al	2006	17145872	0	Mesenchymal-to-epithelial transition facilitates bladder cancer metastasis: role of fibroblast growth factor receptor-2	E-cadherin, β-catenin,vimentin,FGFR3c,FGFR2	
Chakraborty A	2006	16844458	1	Granulocyte colony-stimulating receptor promotes beta1-integrin-mediated adhesion and invasion of bladder cancer cells	G-CSFR,G-CSF,β1-integrin	
Chen CL et al.	2006	16868035	1	Valproic acid inhibits invasiveness in bladder cancer but not in prostate cancer cells	HDACI,HAT,H3Ac,p21,Caspase2,Caspase3,Caspase8,Caspase9	
Cho HJ et al.	2006	16009487	0	Upregulation of Bcl-2 is associated with cisplatin-resistance via inhibition of Bax translocation in human bladder cancer cells	Bcl-2,Caspase3,Caspase6,Caspase7,Caspase8,Caspase9,Caspase10,Bax,Bcl-XL,PARP,FasL,Fas,FADD,Cytochrome c,ASK1	

Table S1. The list of bladder cancer related articles either used or not used RT4.

Author	Year	PMID	using RT4 or not(0 represents No;1 represents Yes)	Title	Gene	Pathway
Christoph F et a	2006	17133271	1	Methylation of tumour suppressor genes APAF-1 and DAPK-1 and in vitro effects of demethylating agents in bladder and kidney cancer	AFAP-1,DAPK-1,	
Earel JK et al.	2006	16397266	0	Histone deacetylase inhibitors modulate the sensitivity of tumor necrosis factor-related apoptosis-inducing ligand-resistant bladder tumor cells	TRAIL,TRAIL-R2,TNF,Acteyl H3,	
El-Zawahry A e	2006	16167063	0	In vitro efficacy of AdTRAIL gene therapy of bladder cancer is enhanced by trichostatin A-mediated restoration of CAR expression and downregulation of cFLIP and Bcl-XL	AdTRAIL,CAR,cFLIP,Bcl-XL,TRAIL,TNF,Mcl-1,XIAP,Bcl-2,HDACi,caspase-8,caspase-3,caspase-7,AdGFP,PARP,p55,p20	
Fuessel S et al.	2006	16458121	0	Chemosensitization of bladder cancer cells by survivin-directed antisense oligodeoxynucleotides and siRNA	Survivin,	
Gee J et al.	2006	16391871	0	Selective cyclooxygenase-2 inhibitors inhibit growth and induce apoptosis of bladder cancer	Cox1,Cox2,Bcl-2,	NF-κB, AP-1, Akt, PPARδ, PPAR γ,STAT1
He YF et al.	2006	17416002	0	onstruction of deltaNp63 specific small hairpin RNA expressing plasmid and its role in bladder cancer--a preliminary study	Np63	
Holterman DA (	2006	16520271	0	Overexpression of alpha-defensin is associated with bladder cancer invasiveness	α-defensin	
Hou JQ et al.	2006	17097022	0	Effect of small interfering RNA targeting survivin gene on biological behaviour of bladder cancer	IAP,Survivin,Abi,	
Inoue M et al.	2006	16310931	0	p53 protein transduction therapy: successful targeting and inhibition of the growth of the bladder cancer cells	p53,11R-p53	
Inoue R et al.	2006	17201133	1	Gefitinib-related gene signature in bladder cancer cells identified by a cDNA microarray	EGFR,YY1,E-cadherin,EGFR-TK1,RTPRH,TNFRSF1B,PGF,TOP2A,TFAP,GADD45A,TRAF5,DUSP9,ZNF161,CAS,DAP6,p38,MAPK14,PTEN,ErbB,	PI3K-AKT
Itoh M et al.	2006	16205632	0	Requirement of STAT3 activation for maximal collagenase-1 (MMP-1) induction by epidermal growth factor and malignant characteristics in T24 bladder cancer cells	STAT3,STAT1,STAT5,EGF,EGFR,MMP-1,c-JUN,AP-1,MMP10,JAK,Bcl-2,Bcl-xl,AP-1,Mcl-1	
Kassouf W et al.	2006	16813948	0	Schedule dependent efficacy of gefitinib and docetaxel for bladder cancer	EGF,EGFR	
Lee JI et al.	2006	16614704	0	beta-lapachone induces growth inhibition and apoptosis in bladder cancer cells by modulation of Bcl-2 family and activation of caspases	Bcl-2,Caspase3,Caspase9,IAP,PCG1,β-catenin,β-lapachone	mitochondrial-signaling pathway
Lin JG et al.	2006	16406939	0	Aloe-emodin induces apoptosis in T24 human bladder cancer cells through the p53 dependent apoptotic pathway	CDK1,Cyclin B1,Wee1,Cdc25c,p53,p21,Bcl-2,Bax,caspase-3,fas,cytochrome C,	
Lodillinsky C et	2006	16391825	0	Bacillus Calmette-Guerin induces the expression of peroxisome proliferator-activated receptor gamma in bladder cancer cells	PPARγ,	
Ma L et al.	2006	16720314	0	Growth inhibitory effects of quercetin on bladder cancer cell	p16,RASSF1,Erb,P53,PTEN,	

Table S1. The list of bladder cancer related articles either used or not used RT4.

Author	Year	PMID	using RT4 or not(0 represents No;1 represents Yes)	Title	Gene	Pathway
Melquist JJ et al	2006	16818192	1	Conditionally replicating adenovirus-mediated gene therapy in bladder cancer: an orthotopic in vivo model	BSP,CAR,	
Memon AA et a	2006	17101554	1	The epidermal growth factor family has a dual role in deciding the fate of cancer cells	EGF,HER1,HER2,HER3,HER4,HRG1, HRG2,HRG3,HRG4,ErbB	
Mey V et al.	2006	16868547	0	In vitro synergistic cytotoxicity of gemcitabine and pemetrexed and pharmacogenetic evaluation of response to gemcitabine in bladder cancer patients	hENT,hCNT,dCK,CDA,RRM1,dCK, 5 ’-NT, CDA, RRM1, RRM2,TS,DHFR,GARFT	
Mohammed SI	2006	16505106	1	Cyclooxygenase inhibitors in urinary bladder cancer: in vitro and in vivo effects	cox1,Cox2,	
Monami G et al.	2006	16849556	0	Proepithelin promotes migration and invasion of 5637 bladder cancer cells through the activation of ERK1/2 and the formation of a paxillin/FAK/ERK complex	IGF-I,IGF-IR,ERK1/2,p90RSK,S6,IF4E,paxillin, FAK,serine,	PI3K,MAPK,Akt
Nagasawa J et a	2006	16771730	0	Novel HER2 selective tyrosine kinase inhibitor, TAK-165, inhibits bladder, kidney and androgen-independent prostate cancer in vitro and in vivo	HER2,TAK-165,HER1,HER3,HER4,p42,p44,FGFR,PDGFR,Jak1,src,blk,	MAPK,Akt
Oka N et al.	2006	16984382	1	Role of phosphatidylinositol-3 kinase/Akt pathway in bladder cancer cell apoptosis induced by tumor necrosis factor-related apoptosis-inducing ligand	TRAIL,Bcl-2,DR4,DR5,	PI3K/Akt
Ornskov D et al	2006	17116246	1	Insulin-induced proliferation of bladder cancer cells is mediated through activation of the epidermal growth factor system	EGF,HER1,HER2, HER3 ,HER4,TGF α,AR,EPI,	
Papageorgiou A	2006	17172406	0	Combination therapy with IFN-alpha plus bortezomib induces apoptosis and inhibits angiogenesis in human bladder cancer cells	IFN-α,TRAIL,PS-341,caspase8,fas,DR3,DR4,DR5,TNFR ,RIP,L32	
Park C et al.	2006	16596191	0	Induction of G2/M arrest and inhibition of cyclooxygenase-2 activity by curcumin in human bladder cancer T24 cells	cox-1,cox-2,cdk2,cdc2,cdc25c,p21,PGE2,cyclin A,cyclinB1,cdc2,p53,p16,p27,	
Pellizzari L et al	2006	17016586	0	PAX8 expression in human bladder cancer	PAX8,WT1,PAX5, PAX2,p53,	
Peng CC et al.	2006	16455193	1	Human urinary bladder cancer T24 cells are susceptible to the Antrodia camphorata extracts	MMP9,cdc2,cyclin B1,MMP2,	
Perabo FG et al.	2006	16827155	1	Indirubin-3'-monoxime, a CDK inhibitor induces growth inhibition and apoptosis-independent up-regulation of survivin in transitional cell cancer	Cdk1,PAGE,PARP,IAP,mAB	
Phelps PT et al.	2006	16581066	0	Characterization of adenosine receptors in the human bladder carcinoma T24 cell line	AMP,adenosine A1,adenosine A2a,adenosine A2b, adenosine A3	
Pocheć E et al.	2006	16373174	0	Characterization of the oligosaccharide component of alpha3beta1 integrin from human bladder carcinoma cell line T24 and its role in adhesion and migration	PVDF,PHA-L	
Rosato A et al.	2006	16678050	1	HYTAD1-p20: a new paclitaxel-hyaluronic acid hydrosoluble bioconjugate for treatment of superficial bladder cancer	CD44 , RHAMM	
Ruifa H et al.	2006	17135787	0	Additional gene therapy with rAAV-wt-p53 enhanced the efficacy of cisplatin in human bladder cancer cells	P53,P21,bax,EGFR,	

Table S1. The list of bladder cancer related articles either used or not used RT4.

Author	Year	PMID	using RT4 or not(0 represents No;1 represents Yes)	Title	Gene	Pathway
Shan Y et al.	2006	16964384	0	Effect of sulforaphane on cell growth, G(0)/G(1) phase cell progression and apoptosis in human bladder cancer T24 cells	p27,p16,p15,CDK2,CDK4,CDK6,CKI ,cyclinD	
Shen SS et al.	2006	16700038	1	Expression of estrogen receptors-alpha and -beta in bladder cancer cell lines and human bladder tumor tissue	Erβ,Erα,Ki67,cyclinA	
Svatek RS et al.	2006	16541433	0	Soluble Fas--a promising novel urinary marker for the detection of recurrent superficial bladder cancer	NMP22,Fas	
Ting AH et al.	2006	16424002	0	Differential requirement for DNA methyltransferase 1 in maintaining human cancer cell gene promoter hypermethylation	DNMT1Cdkn2a,strp1,Gata4,DNMT3b	
Tyagi A et al.	2006	16777994	1	Silibinin activates p53-caspase 2 pathway and causes caspase-mediated cleavage of Cip1/p21 in apoptosis induction in bladder transitional-cell papilloma RT4 cells: evidence for a regulatory loop between p53 and caspase 2	JNK1/2 ,p21,ATM,caspase8,bid,caspase9,casp ase3,RARP,Chk2,H2A,cytochrome C	p53-caspase 2,ATM-Chk2
Wang H et al.	2006	16979729	0	Oncolytic viral therapy by bladder instillation using an E1A, E1B double-restricted adenovirus in an orthotopic bladder cancer model	p14ARF and mdm2	p53,pRb, p16 pathway
Wang L et al.	2006	16961280	0	Smac/DIABLO promotes mitomycin C-induced apoptosis of bladder cancer T24 cells	DIABLO	
Watanabe J et al	2006	16518417	0	Dicoumarol potentiates cisplatin-induced apoptosis mediated by c-Jun N-terminal kinase in p53 wild-type urogenital cancer cell lines	bax,c-jun,p38,ERK,Bcl-2,caspase8,caspase9,caspase3,PARP	p53/p21/JNK pathways
Yamamoto Net	2006	17062641	0	Tyrosine phosphorylation of p145met mediated by EGFR and Src is required for serum-independent survival of human bladder carcinoma cells	EGFR,Src,p145,Yes,Fyn,p42,p44,c-Met,HGF,mAb	MAPK
Yin ZF et al.	2006	17054846	1	Adriamycin and mitomycin dose-dependently downregulate X-kinked inhibitor of apoptosis protein in human bladder cancer cells	XIAP,caspase3,	
Yoshida T et al.	2006	16624424	0	Expression and cellular localization of dbpC/Contrin in germ cell tumor cell lines	c-myc,N-myc,dbpc,dbpb,	
Yuan SS et al.	2006	16154156	0	Selective cytotoxicity of squamocin on T24 bladder cancer cells at the S-phase via a Bax-, Bad-, and caspase-3-related pathways	Bad,Bax,procaspase-3,Bcl-xl,Bcl-2,PARP,p21,Chk2,	
Zou L et al.	2006	16765199	0	Mad1 suppresses bladder cancer cell proliferation by inhibiting human telomerase reverse transcriptase transcription and telomerase activity	Mad-1,c-myc,tw,hTERT	
Zou L et al.	2006	16028098	0	shRNA-targeted hTERT suppress cell proliferation of bladder cancer by inhibiting telomerase activity	hTERT,c-Myc,Max,Mad	
Canes D, Chian[illegible]	2005	15499627	0	Histone deacetylase inhibitors upregulate plakoglobin expression in bladder carcinoma cells and display antineoplastic activity in vitro and in vivo	N-cadherin，E-cadherin	
Eisenhardt A, F[illegible]	2005	15582259	0	Expression analysis and potential functional role of the CXCR4 chemokine receptor in bladder cancer	CXCR4	
Fechner G, Ded	2005	16101157	1	Hyperoxia-induced improvement of the in vitro response to gemcitabine in transitional cell carcinoma	VEGF，HIF-1alpha	

Table S1. The list of bladder cancer related articles either used or not used RT4.

Author	Year	PMID	using RT4 or not(0 represents No;1 represents Yes)	Title	Gene	Pathway
Fu WJ, Hong Bl	2005	16153224	0	Expression of a recombinant vector of a mutant human telomerase reverse transcriptase gene in human bladder cancer cell line T24, and its clinical significance	hTERT	
Girouard J, Rey	2005	15829265	0	Requirement of the extracellular cysteine at position six for CD40/CD40 dimer formation and CD40-induced IL-8 expression	CD40，IL-8	
Huygens A, Kai	2005	15947580	1	Permeation of hypericin in spheroids composed of different grade transitional cell carcinoma cell lines and normal human urothelial cells	E-cadherin	
Ko WS, Park TY	2005	16012733	0	Induction of apoptosis by Chan Su, a traditional Chinese medicine, in human bladder carcinoma T24 cells	Bcl-2,Bcl-X,Bax,caspase-3,caspase-9, COX-2, COX-1,PGE2	
Kong C, Zhu Y,	2005	15922420	1	Role of protein kinase C-alpha in superficial bladder carcinoma recurrence	PKC-a	PKC
Kuncová J, Kos	2005	15783086	0	Expression of CD44v6 correlates with cell proliferation and cellular atypia in urothelial carcinoma cell lines 5637 and HT1197	CD44	
Li C, Teng RH,	2005	15611796	0	H-Ras oncogene counteracts the growth-inhibitory effect of genistein in T24 bladder carcinoma cells	H-ras,c-fos,egr-1,VDUP-1	MEK/ERK, JNK
Li J, Yao S, Zha	2005	15989974	0	The role of c-Jun in the AP-1 activation induced by naturally occurring isothiocyanates	AP-1,c-Jun,c-fos,fra-1,JNK	
Li TM, Chen GV	2005	15868936	0	Ellagic acid induced p53/p21 expression, G1 arrest and apoptosis in human bladder cancer T24 cells	caspases-3 ,p53,p21,CDK2	
Lin SY, Yang JI	2005	16314733	0	Effect of inhibition of aloe-emodin on N-acetyltransferase activity and gene expression in human malignant melanoma cells (A375.S2)	NAT	
Liu J, Li AP, Li	2005	16124896	0	[The role of reactive oxygen species in N-[4-hydroxyphenyl] retinamide induced apoptosis in bladder cancer cell lineT24]	XRCC1，caspase-3	
Memon AA, So	2005	15701847	1	Down-regulation of S100C is associated with bladder cancer progression and poor survival	S100C	
Moussa O, Yorc	2005	16357168	1	Prognostic and functional significance of thromboxane synthase gene overexpression in invasive bladder cancer	TXAS,TXA2	
Nogawa M, Yua	2005	15761500	1	Intravesical administration of small interfering RNA targeting PLK-1 successfully prevents the growth of bladder cancer	PLK-1 ,cyclin B1	
Ogishima T, Sh	2005	16007175	0	Promoter CpG hypomethylation and transcription factor EGR1 hyperactivate heparanase expression in bladder cancer	EGR1,heparanase	
Reitmair A, Shu	2005	15729717	1	Retinoid-related molecule AGN193198 potently induces G2M arrest and apoptosis in bladder cancer cells	caspase-3，caspase-8，caspase-9	
Rocchetti R, Tal	2005	16224178	0	Antiangiogenic drugs for chemotherapy of bladder tumours	bFGF，VEGF	
Sasaki T, Yoshi	2005	15834587	0	Heme oxygenase-1 accelerates protumoral effects of nitric oxide in cancer cells	Bcl-2，VEGF，HO-1	
Shariat SF, Mats	2005	15967254	1	Urinary levels of soluble e-cadherin in the detection of transitional cell carcinoma of the urinary bladder	E-cadherin	

Table S1. The list of bladder cancer related articles either used or not used RT4.

Author	Year	PMID	using RT4 or not(0 represents No;1 represents Yes)	Title	Gene	Pathway
Smakman N, Sc	2005	16098373	0	NS-398, a selective cyclooxygenase-2 inhibitor, reduces experimental bladder carcinoma outgrowth by inhibiting tumor cell proliferation	COX-2，PGE2，Ki-67，caspase-3，CD31	
Sudarshan S, H	2005	15514684	0	In vitro efficacy of Fas ligand gene therapy for the treatment of bladder cancer	PARP,caspase-3，caspase-7，caspase-8，caspase-9	
Tang L, Zhang	2005	16093441	0	Mitochondria are the primary target in isothiocyanate-induced apoptosis in human bladder cancer cells	Bcl-2，Bak，Bcl-xl，Bax，caspase-3,caspase-9,PARP,Cytochrome C	
Xin Y, Lyness (	2005	15947683	1	Low dose suramin as a chemosensitizer of bladder cancer to mitomycin C	Ki-67	
Yamada H, Luo	2005	15711363	1	A novel expression of macrophage derived chemokine in human bladder cancer	MDC,IP-10,TNF-a, IFN-r,NF-kB	
Yamada H, Odo	2005	16094077	1	Interferon-gamma up-regulates toll-like receptor 4 and cooperates with lipopolysaccharide to produce macrophage-derived chemokine and interferon-gamma inducible protein-10 in human bladder cancer cell line RT4	MDC,IP-10,TNF-a, IFN-r,NF-kB,AP-1, ERK1/2,TLR-4,CD14	NF-kappaB,AP-1/ERK1/2
Zhu YY, Wang	2005	16008942	1	[The role of protein kinase C alpha in recurrence of superficial bladder carcinoma]	PKCalpha	
Zi X, Simoneau	2005	15833884	1	Flavokawain A, a novel chalcone from kava extract, induces apoptosis in bladder cancer cells by involvement of Bax protein-dependent and mitochondria-dependent apoptotic pathway and suppresses tumor growth in mice	Bcl-x,Bax,PARP,caspase-9,caspase-3,survivin,XIAP	Bax-dependent mitochondrial apoptotic pathway
Zou L, Zhang P	2005	15645079	0	Transcript regulation of human telomerase reverse transcriptase by c-myc and mad1	c-myc,mad1, hTERT	
Bar-Haim, E.	2004	15213716	1	MAGE-A8 overexpression in transitional cell carcinoma of the bladder: identification of two tumour-associated antigen peptides	MAGE-A8	
Bernard-Pierrot,	2004	15516981	0	Inhibition of human bladder tumour cell growth by fibroblast growth factor receptor 2b is independent of its kinase activity. Involvement of the carboxy-terminal region of the receptor	FGFR2b	
Chatterjee, S. J.	2004	15221935	0	Hyperphosphorylation of pRb: a mechanism for RB tumour suppressor pathway inactivation in bladder cancer	Rb	
Diermeier, S.	2004	15030553	1	Exposure to continuous bromodeoxyuridine (BrdU) differentially affects cell cycle progression of human breast and bladder cancer cell lines	BrdU	cell cycle
Kim, I. Y.	2004	15492256	1	Restoration of bone morphogenetic protein receptor type II expression leads to a decreased rate of tumor growth in bladder transitional cell carcinoma cell line TSU-Pr1	BMP-RII,BMP-RIA,BMP-RIB	BMP-RII
Kinjo, M.	2004	14678183	1	Detection of circulating MUC7-positive cells by reverse transcription-polymerase chain reaction in bladder cancer patients	MUC7	MUC7
Li, G.	2004	15301717	1	[Enhancement effect of interferon gamma on the sensitivity of RT4 bladder cancer cells to 5'-deoxy-5-fluorouridine,and 5-fluorouracil through up-regulation of PD-ECGF/TP]	INFgamma	INFgamma

Table S1. The list of bladder cancer related articles either used or not used RT4.

Author	Year	PMID	using RT4 or not(0 represents No;1 represents Yes)	Title	Gene	Pathway
Muramaki, M.	2004	14665947	1	Over expression of CD44V8-10 in human bladder cancer cells decreases their interaction with hyaluronic acid and potentiates their malignant progression	CD44	
Ning, S.	2004	15375557	1	siRNA-mediated down-regulation of survivin inhibits bladder cancer cell growth	Survivin	Survivin
Nutt, J. E.	2004	15083203	1	Gefitinib ('Iressa', ZD1839) inhibits the growth response of bladder tumour cell lines to epidermal growth factor and induces TIMP2	EGFR	EGFR
Oliveira-Ferrer,	2004	15604255	1	Dual role of carcinoembryonic antigen-related cell adhesion molecule 1 in angiogenesis and invasion of human urinary bladder cancer	CEACAM1	CEACAM1
Perabo, F. G.	2004	15517890	1	Carboxyamido-triazole (CAI), a signal transduction inhibitor induces growth inhibition and apoptosis in bladder cancer cells by modulation of Bcl-2	bcl-2	bcl-2
Sachs, M. D	2004	15118762	1	Histone deacetylase inhibitors upregulate expression of the coxsackie adenovirus receptor (CAR) preferentially in bladder cancer cells	HDACI	HDACI
Schaaf, A.	2004	15271313	1	Cytotoxicity of cisplatin in bladder cancer is significantly enhanced by application of bcl-2 antisense oligonucleotides	bcl-2	bcl-2
Sugimoto, S.	2004	14732229	1	Expression and regulation of tumor suppressor gene maspin in human bladder cancer	Maspin	Maspin
Abdel-Mageed .	2003	14574533	0	NF-kappaB-dependent gene expression of proinflammatory cytokines in T24 cells: possible role in interstitial cystitis	NF-kappaB/TNF-α/IL-1β/IL-6/IL-8	
BILIM V	2003	12455050	1	Role of XIAP in the malignant phenotype of transitional cell cancer (TCC) and therapeutic activity of XIAP antisense oligonucleotides against multidrug-resistant TCC in vitro	XIAP/Bcl-2/NF-κB/Casepase3	
Champelovier P	2003	14555531	1	Is interferon gamma one key of metastatic potential increase in human bladder carcinoma?	IFN- γ/TNF-α/PAI1和PAI2/IL-1α/β，IL-2，IL-5，IL-6，IL-8，IL-10/CSF-1	
Chan MW 1，C	2003	12594816	0	Frequent hypermethylation of promoter region of RASSF1A in tumor tissues and voided urine of urinary bladder cancer patients	RASSF1A	RAS
Chen, F.Langen	2003	14532843	0	Androgen dependent regulation of bacillus Calmette-Guerin induced interleukin-6 expression in human transitional carcinoma cell lines	NF-κB/ IL-6 NF-κB	
Fechner, G.Pera	2003	12597983	1	Preclinical evaluation of a radiosensitizing effect of gemcitabine in p53 mutant and p53 wild type bladder cancer cells	p53	
Gupta AK 1，C	2003	12788194	0	Radiation sensitization of human cancer cells in vivo by inhibiting the activity of PI3K using LY294002	PI3K/Akt/Ras/EGFR/PTEN	Ras-PI3K pathway
He D 1，Nan X	2003	14527374	0	Overexpression of the promyelocytic leukemia gene suppresses growth of human bladder cancer cells by inducing G1 cell cycle arrest and apoptosis	PML	

Table S1. The list of bladder cancer related articles either used or not used RT4.

Author	Year	PMID	using RT4 or not(0 represents No;1 represents Yes)	Title	Gene	Pathway
Hinata, N.Shiral	2003	12955365	1	Radiation induces p53-dependent cell apoptosis in bladder cancer cells with wild-type- p53 but not in p53-mutated bladder cancer cells	P53/Bax/p21	
Karashima T 1，	2003	12855659	1	Nuclear factor-kappaB mediates angiogenesis and metastasis of human bladder cancer through the regulation of interleukin-8	NF-κB/IL-8/IkappaB-α	NF-κB
Kyker KD 1，C	2003	12954494	1	A model for 3-dimensional growth of bladder cancers to investigate cell-matrix interactions	E-cadherin /	cadherin-B-catenin-cytoskeleton
Lou, T. F.Gray,	2003	15000822	0	The reduction of Raf-1 protein by phosphorothioate ODNs and siRNAs targeted to the same two mRNA sequences	Raf-1/Bcl-2/	
McKenna DJ 1，	2003	12893084	1	Modification of the alkaline Comet assay to allow simultaneous evaluation of mitomycin C-induced DNA cross-link damage and repair of specific DNA sequences in RT4 cells	TP53	
McKenna DJ1,	2003	12492368	1	Use of the comet-FISH assay to demonstrate repair of the TP53 gene region in two human bladder carcinoma cell lines	TP53	
Morita, T.Matsu	2003	14657601	0	Forced expression of cytidine deaminase confers sensitivity to capecitabine	CDD2/TP/DPD/TS	
Muramaki, M.M	2003	14613994	0	Introduction of midkine gene into human bladder cancer cells enhances their malignant phenotype but increases their sensitivity to antiangiogenic therapy	MK	
Nutt, J. E.Durka	2003	12614260	1	Matrix metalloproteinases (MMPs) in bladder cancer: the induction of MMP9 by epidermal growth factor and its detection in urine	MMPs/EGF/MMP2/MMP9/MMP1	
Pagliaro, L. C.K	2003	14693272	0	Adenoviral p53 gene transfer in human bladder cancer cell lines: cytotoxicity and synergy with cisplatin	p53/p21/BAX	
Paredes N 1，X	2003	12542493	0	The effects of chemotherapeutic agents on the regulation of thrombin on cell surfaces	TF	
Su CC 1，Chen	2003	12680237	0	Luteolin induces N-acetylation and DNA adduct of 2-aminofluorene accompanying N-acetyltransferase activity and gene expression in human bladder cancer T24 cell line	NAT1	
Tanaka M 1，G	2003	12923562	0	In vivo gene therapy of human bladder cancer with PTEN suppresses tumor growth, downregulates phosphorylated Akt, and increases sensitivity to doxorubicin	PTEN/ P-Akt	
Tyagi, A. K.Aga	2003	14651997	1	Silibinin down-regulates survivin protein and mRNA expression and causes caspases activation and apoptosis in human bladder transitional-cell papilloma RT4 cells	Survivin/PARP/caspase-3,9	
Watson, L. M.C	2003	12621026	0	Overexpression of the 78-kDa glucose-regulated protein/immunoglobulin-binding protein (GRP78/BiP) inhibits tissue factor procoagulant activity	GRP78/BiP	
Wu, W.Shu, X.	2003	12776187	1	VEGF receptor expression and signaling in human bladder tumors	VEGF / Flk-1 /GAP/Ras/PKC/SPK/ERK1 / 2	VEGF pathway

Table S1. The list of bladder cancer related articles either used or not used RT4.

Author	Year	PMID	using RT4 or not(0 represents No;1 represents Yes)	Title	Gene	Pathway
Yang CC 1，Ch	2003	12787261	0	Paclitaxel (taxol) inhibits the arylamine N-acetyltransferase activity and gene expression (mRNA NAT1) and 2-aminofluorene-DNA adduct formation in human bladder carcinoma cells (T24 and TSGH 8301)	NAT1	
Yuan SS 1，Ch	2003	12697268	0	Annonacin, a mono-tetrahydrofuran acetogenin, arrests cancer cells at the G1 phase and causes cytotoxicity in a Bax- and caspase-3-related pathway	Bax- / caspase-3/p53/BAD/BAX/P21/Chk1/Chk2	
Zhang Z1, Shira	2003	14533194	0	Combination with CD/5-FC gene therapy enhances killing of human bladder-cancer cells by radiation	CD/5-FC/p53 /CD	
Mihaela Velices	2002	11956100	0	Cell Division Is Required for de Novo Methylation of CpG Islands in Bladder Cancer Cells	p16、DNMT	
Jane Zhang	2002	12097284	1	Identification of human uroplakin II promoter and its use in the construction of CG8840, a urothelium-specific adenovirus variant that eliminates established bladder tumors in combination with docetaxel	hUPII、	
Yuan SY et al.	2002	12389115	0	Involvement of mitochondrial pathway in Taxol-induced apoptosis of human T24 bladder cancer cells	Bax、Bcl-2、Bcl-XL、Fas、FasL、caspase-3	
Ibtissam Youlyo	2002	12467527	0	Expression of a splice variant of the platelet-activating factor receptor transcript 2 in various human cancer cell lines	PAF-R	
Guangbin Xia	2002	11992927	0	Positive expression of HIF-2α/EPAS1 in invasive bladder cancer	HIF-2α/EPAS1	
Vecchione A et	2002	1867220	0	FEZ1/LZTS1 is down-regulated in high-grade bladder cancer, and its restoration suppresses tumorigenicity in transitional cell carcinoma cells	FEZ1/LZTS1 GFP cdc2	
van der Poel, H.	2002	12050554	0	Epidermal growth factor receptor targeting of replication competent adenovirus enhances cytotoxicity in bladder cancer	EGF-R	
Stoehr, R. et al.	2002	11956582	1	No evidence for involvement of beta-catenin and APC in urothelial carcinomas	APC CTNNB1	
Shu, X. et al.	2002	12391145	0	Sphingosine kinase mediates vascular endothelial growth factor-induced activation of ras and mitogen-activated protein kinases	PKC、SPK、Ras、Raf、ERK1 、ERK2	PKC-Ras
Sanchez-Carbay	2002	12460915	1	Molecular profiling of bladder cancer using cDNA microarrays: defining histogenesis and biological phenotypes	TP53、pRB、INK4A / p16	
Przybylo, M. et	2002	12234377	0	Different glycosylation of cadherins from human bladder non-malignant and cancer cell lines	GNA、MAA、SNA、PHA-L、DSA、AAA	
Nguyen, C. T. e	2002	12438235	0	Histone H3-lysine 9 methylation is associated with aberrant gene silencing in cancer cells and is rapidly reversed by 5-aza-2'-deoxycytidine	p14 promoter、p16 promoter、p14/p16 exon 2、GAPDH	
Luan, F. L.	2002	12042641	0	Rapamycin blocks tumor progression: unlinking immunosuppression from antitumor efficacy	p27Kip1、Cyclin D1、	
James, N. J.	2002	12023854	0	Adhesion properties of human bladder cell lines with extracellular matrix components: the role of integrins and glycosylation	CD29、GPIIa、P1B5、P1A6	

Table S1. The list of bladder cancer related articles either used or not used RT4.

Author	Year	PMID	using RT4 or not(0 represents No;1 represents Yes)	Title	Gene	Pathway
Liang, G.	2002	11861364	0	Analysis of gene induction in human fibroblasts and bladder cancer cells exposed to the methylation inhibitor 5-aza-2'-deoxycytidine	KRT8、KRT17、H19、TIMP3、STAT1、GAPDH、SAA1	
Lang, K.	2002	12115500	0	Signal processing in migrating T24 human bladder carcinoma cells: role of the autocrine interleukin-8 loop	interleukin-8	interleukin-8 loop
Kim, D. K.	2002	12225859	0	Characterization of the system L amino acid transporter in T24 human bladder carcinoma cells	LAT1、LAT2	
Kaasinen, E. S.	2002	11956480	0	Inhibition of natural, interleukin-2 stimulated and bacillus Calmette-Guerin enhanced cytotoxicity with anti-CD16 antibodies	IL-2	
Hong, J. H.	2002	12081782	0	Antisense Bcl2 oligonucleotide in cisplatin-resistant bladder cancer cell lines	Bcl2、	
Hayes, G. M.	2002	11857030	0	Alternative splicing as a novel of means of regulating the expression of therapeutic genes	CD44R1、CD44R2、CD44H、ALP、pCEP4	
Greco, O.	2002	12365006	0	Novel chimeric gene promoters responsive to hypoxia and ionizing radiation	EGFP、PGK1、E9	
Greco, O.	2002	12244574	0	Mechanisms of cytotoxicity induced by horseradish peroxidase/indole-3-acetic acid gene therapy	HRP、IAA	HRP/IAA-GDEPT
Feleszko, W.	2002	12066226	0	Synergistic interaction between highly specific cyclooxygenase-2 inhibitor, MF-tricyclic and lovastatin in murine colorectal cancer cell lines	COX-2、	
Fauconnet, S.	2002	11980898	1	Differential regulation of vascular endothelial growth factor expression by peroxisome proliferator-activated receptors in bladder cancer cells	VEGF、β-actin	p38 MAP kinase、PI 3-kinase
Benimetskaya, I	2002	11906253	0	Protamine-fragment peptides fused to an SV40 nuclear localization signal deliver oligonucleotides that produce antisense effects in prostate and bladder carcinoma cells	PKC-α、G3139、bcl-2、SV40	
Yang, C. C. and	2001	11828994	0	Sulindac inhibited gene expression and activity of arylamine N-acetyltransferase and DNA-2-aminofluorene adduct formation in T24 human bladder tumor cells	NAT	
Waters, S. B. an	2001	11738937	0	A new assay to quantify in vivo repair of G:T mispairs by base excision repair.	supF	
Thykjaer, T., et	2001	11506498	1	Functional analysis of the mismatch repair system in bladder cancer	MSH2, MSH3, MSH6, PMS1, PMS2 and MLH1	
Tanaka, M. and	2001	11779406	0	Connexin 26 gene therapy of human bladder cancer: induction of growth suppression, apoptosis, and synergy with Cisplatin.	Connexin 26 cyclinB Bcl-2	
Tanaka, M., et a	2001	11313783	0	Connexin 26 enhances the bystander effect in HSVtk/GCV gene therapy for human bladder cancer by adenovirus/PLL/DNA gene delivery	Connexin 26 HSVtk	
Sun, H. Z., et al	2001	11453542		Blockage of IGF-1R signaling sensitizes urinary bladder cancer cells to mitomycin-mediated cytotoxicity	IGF-1 IGF-2	IGF-1R
Sun, H. Z., et al	2001	11749868	0	Knockdown of IGF-IR by Antisense Oligodeoxynucleotide auguments the sensitivity of bladder cancer cells to mitomycin	IGF-1 IGF-3	IGF-2R

Table S1. The list of bladder cancer related articles either used or not used RT4.

Author	Year	PMID	using RT4 or not(0 represents No;1 represents Yes)	Title	Gene	Pathway
Suda, K., et al.	2001	11669284	0	Phenotypic characterization of human umbilical vein endothelial (ECV304) and urinary carcinoma (T24) cells: endothelial versus epithelial features	vWF vimentin VE-cadherin PECAM-1 cytokeratin 8 cytokeratin 18 E-cadherin desmoglein	
Shichinohe, T.,	2001	11773978	0	Development of lentiviral vectors for antiangiogenic gene delivery.	angiostatin and endostatin	
Seidl, J., et al.	2001	11340570	1	Optimization of differential photodynamic effectiveness between normal and tumor urothelial cells using 5-aminolevulinic acid-induced protoporphyrin IX as sensitizer	5-ALA	
Reiher, F. K., et	2001	11371931	1	The role of hypoxia and p53 in the regulation of angiogenesis in bladder cancer.	TSP-1 VEGF p53	
Pervaiz, S., et al	2001	11593437	0	Activation of the RacGTPase inhibits apoptosis in human tumor cells.	Rac1	Rac /氧化酶/ O 2
Perabo, F. G., et	2001	11418323	1	Soluble Fas and Fas-ligand in bladder cancer in vitro and in vivo.	sFas	
Perabo, F. G., et	2001	11355943	1	Bladder cancer cells acquire competent mechanisms to escape Fas-mediated apoptosis and immune surveillance in the course of malignant transformation	Fas	
Ono, Y., et al.	2001	11689148	0	Loss of p73 induction in a cisplatin-resistant bladder cancer cell line.	P73	
Okegawa, T., et	2001	11522659	1	The mechanism of the growth-inhibitory effect of coxsackie and adenovirus receptor (CAR) on human bladder cancer: a functional analysis of car protein structure	CAR	
Oikawa, M., et a	2001	11708773	0	Hypoxia induces transcription factor ETS-1 via the activity of hypoxia-inducible factor-1	HIF-1 ETS-1	
Nakashiro, K. I.	2001	11485917	1	Role of peroxisome proliferator-activated receptor gamma and its ligands in non-neoplastic and neoplastic human urothelial cells	PPARγ	
Mizutani, Y., et	2001	11125422	0	Enhanced sensitivity of bladder cancer cells to tumor necrosis factor related apoptosis inducing ligand mediated apoptosis by cisplatin and carboplatin.	TRAIL	
McHowat, J., et	2001	11371929	1	Stimulation of protease activated receptors on RT4 cells mediates arachidonic acid release via Ca2+ independent phospholipase A2	PLA2	
McGarvey, T. W	2001	11418322	1	Growth inhibitory effect of p21 and p53 containing adenoviruses on transitional cell carcinoma cell lines in vitro and in vivo.	p21 p53	
Matin, S. F., et a	2001	11280796	0	Impaired alpha-interferon signaling in transitional cell carcinoma: lack of p48 expression in 5637 cells	IFN-α	
Macville, M. V.	2001	11455032	0	Spectral imaging of multi-color chromogenic dyes in pathological specimens	Ki-67 TP53	
Liu, J., et al	2001	11245599	0	Caveolin-1 expression sensitizes fibroblastic and epithelial cells to apoptotic stimulation	caveolin-1	
Lee, C. T., et al	2001	11205911	0	Differential effects of adenovirus-p16 on bladder cancer cell lines can be overcome by the addition of butyrate	p16	
Lebedeva, I., et	2001	11458048	0	Chemosensitization of bladder carcinoma cells by bcl-xL antisense oligonucleotides	bcl-xL	

Table S1. The list of bladder cancer related articles either used or not used RT4.

Author	Year	PMID	using RT4 or not(0 represents No;1 represents Yes)	Title	Gene	Pathway
Langzam, L., et	2001	11554166	1	Patterns of protein kinase C isoenzyme expression in transitional cell carcinoma of bladder. Relation to degree of malignancy	pkc	
Kawakami, S., e	2001	11299737	0	Inhibitory effect of N-acetylcysteine on invasion and MMP-9 production of T24 human bladder cancer cells	mmp9	
Jones, A., et al.	2001	11350893	1	Relation of vascular endothelial growth factor production to expression and regulation of hypoxia-inducible factor-1 alpha and hypoxia-inducible factor-2 alpha in human bladder tumors and cell lines.	VEGF	
Duggan, B. J., e	2001	11490306	1	The effect of antisense Bcl-2 oligonucleotides on Bcl-2 protein expression and apoptosis in human bladder transitional cell carcinoma.	bcl2	
Duggan, B. J., e	2001	11805418	1	Antisense Bcl-2 oligonucleotide uptake in human transitional cell carcinoma	bcl2	
Chabannes, E.,	2001	11483411	1	Protein kinase C signalling pathway is involved in the regulation of vascular endothelial growth factor expression in human bladder transitional carcinoma cells	VEGF	
Bostrom, P. J., e	2001	11310210	0	Interferon-alpha inhibits cyclooxygenase-1 and stimulates cyclooxygenase-2 expression in bladder cancer cells in vitro	cox-1 cox-2	
Bostrom, P. J., e	2001	11827414	1	Expression of cyclooxygenase-1 and -2 in urinary bladder carcinomas in vivo and in vitro and prostaglandin E2 synthesis in cultured bladder cancer cells	cox-1 cox-2	
Bindels, E. M.,	2001	11710643	0	Influence of the microenvironment on invasiveness of human bladder carcinoma cell lines	E-cadherin	
Benimetskaya, I	2001	11723237	0	Inhibition of potentially anti-apoptotic proteins by antisense protein kinase C-alpha (Isis 3521) and antisense bcl-2 (G3139) phosphorothioate oligodeoxynucleotides: relationship to the decreased viability of T24 bladder and PC3 prostate cancer cells	Isis 3521 G3139	
Zlotta, A. R. et a	2000	10956396	1	What are the immunologically active components of bacille Calmette-Guerin in therapy of superficial bladder cancer?	FN-gamma, IL-12, IL-2, IL-6	
Zhou, Q. et al.	2000	10623623	1	Contortrostatin, a homodimeric disintegrin, binds to integrin alphavbeta5	alphavbeta5	
Wu, H. C. et al.	2000	11011961	0	Inhibition by vitamin C of DNA adduct formation and arylamine N-acetyltransferase activity in human bladder tumor cells	NAT	
Watanabe, T. et	2000	10995035	1	Significance of the Grb2 and son of sevenless (Sos) proteins in human bladder cancer cell lines	EGF	
Tanaka, M. et a	2000	11103942	1	MMAC1/PTEN inhibits cell growth and induces chemosensitivity to doxorubicin in human bladder cancer cells	MMAC1、PTEN	MMAC1/PTEN
Sun, H. et al.	2000	11778548	0	Effect of IGF1 receptor gene antisense oligodeoxynucleotide on T24 urinary bladder cancer cells	IGF1、IGF1R	
Seraj, M. J. et al	2000	11592309	1	The relationship of BRMS1 and RhoGDI2 gene expression to metastatic potential in lineage related human bladder cancer cell lines	BRMS1 、 RhoGDI2	

Table S1. The list of bladder cancer related articles either used or not used RT4.

Author	Year	PMID	using RT4 or not(0 represents No;1 represents Yes)	Title	Gene	Pathway
Richon, V. M. e	2000	10954755	0	Histone deacetylase inhibitor selectively induces p21WAF1 expression and gene-associated histone acetylation	p21WAF1	
Price, M. E. et a	2000	10825754	0	Induction and rejoining of DNA double-strand breaks in bladder tumor cells	DSBs	
Mialhe, A. et al.	2000	10953163	0	Expression of E-, P-, n-cadherins and catenins in human bladder carcinoma cell lines	E-, P-, n-cadherins , catenins	
Ma, M.et al.	2000	15640448	0	Intracellular mRNA cleavage induced through activation of RNase P by nuclease-resistant external guide sequences	EGS、PKC-alpha	
Lu, Y. et al.	2000	10854143	0	Transcriptionally regulated adenoviruses for prostate-specific gene therapy	PSA、lacZ	
Laidler, P. et al.	2000	11996105	0	Expression of beta1-integrins and N-cadherin in bladder cancer and melanoma cell lines	beta1-integrins 、 N-cadherin	
Krieg, R. C. et a	2000	10946577	0	Cell-type specific protoporphyrin IX metabolism in human bladder cancer in vitro	PPIX、PBGD	
Kamuhabwa, A.	2000	10953329	0	Photodynamic activity of hypericin in human urinary bladder carcinoma cells	hypericin	
Hop, C. et al.	2000	10894814	0	Assembly of multimeric von Willebrand factor directs sorting of P-selectin	vWF	kinase C
He, T. et al.	2000	10774927	0	Intracellular sequestration of anti-tumor drugs by metallothionein	HPLC	
Hall, M. C. et al	2000	10688044	1	The growth inhibitory effect of p21 adenovirus on human bladder cancer cells	p21	
Gupta, A. K. et	2000	10856967	0	RAS-Mediated radiation resistance is not linked to MAP kinase activation in two bladder carcinoma cell lines	MAPK	RAF-MAP2K-MAP kinase
Gildea, J. J. et a	2000	10679914	1	Genetic and phenotypic changes associated with the acquisition of tumorigenicity in human bladder cancer	LOH	
Fujii, Y. et al.	2000	10859542	0	Significance of carbohydrate antigen sialyl-Lewis X, sialyl-Lewis A, and possible unknown ligands to adhesion of human urothelial cancer cells to activated endothelium	E-selectin	
Freund, C. T. et	2000	11062688	1	Adenovirus-mediated suicide gene therapy for bladder cancer: comparison of the cytomegalovirus- and Rous sarcoma virus-promoter	ADV/CMV-tk	
Cos, J. et al.	2000	10834027	0	Comparative study of sequential combinations of paclitaxel and methotrexate on a human bladder cancer cell line	Taxol, TX	
Cohen-Jonathan	2000	10931682	1	Farnesyltransferase inhibitors potentiate the antitumor effect of radiation on a human tumor xenograft expressing activated HRAS	RAS	
Brown, J. et al.	2000	10653001	1	Critical evaluation of ECV304 as a human endothelial cell model defined by genetic analysis and functional responses: a comparison with the human bladder cancer derived epithelial cell line T24/83	P2Y2	
Bostrom, P. J.et	2000	11054671	1	Expression of collagenase-3 (matrix metalloproteinase-13) in transitional-cell carcinoma of the urinary bladder	MMP-13、TNF-alpha、p38	

Table S1. The list of bladder cancer related articles either used or not used RT4.

Author	Year	PMID	using RT4 or not(0 represents No;1 represents Yes)	Title	Gene	Pathway
Bonnal, C. et al.	2000	10688097	0	Absence of microsatellite instability in transitional cell carcinoma of the bladder	BAT26, TGFbetaRII, BAX	
Bogliolo, M. et	2000	11126365	0	Reduced ligation during DNA base excision repair supported by BRCA2 mutant cells	BRCA1、BRCA2	
Bindels, E. M. e	2000	10646871	0	E-cadherin promotes intraepithelial expansion of bladder carcinoma cells in an in vitro model of carcinoma in situ	E-cadherin	
Bilim, V. et al.	2000	11277327	0	Adriamycin induced G2/M cell cycle arrest in transitional cell cancer cells with wt p53 and p21(WAF1/CIP1) genes	Hematuria	
Bilim, V. et al.	2000	10822135	0	Caspase involved synergistic cytotoxicity of bcl-2 antisense oligonucleotides and adriamycin on transitional cell cancer cells	Bcl-2、Caspases	
Baffa, R. et al.	2000	10666370	1	Loss of FHIT expression in transitional cell carcinoma of the urinary bladder	FHIT	
Arai, Y. et al.	2000	10807969	1	Limitations of urinary telomerase activity measurement in urothelial cancer	telomerase	
Beck TP et al.	1999	10475376	1	In vitro evaluation of calphostin C as a novel agent for photodynamic therapy of bladder cancer	p21,p53,pRb	
Davies G et al.	1999	10226596	0	Cell-cell adhesion molecules and their associated proteins in bladder cancer cells and their role in mitogen induced cell-cell dissociation and invasion	E-cadherin,N-cadherin,α-catenin,β-catenin,γ-catenin,desmoglein,c-MET,HGF/SF	
Elkin M et al.	1999	10473075	0	Inhibition of matrix metalloproteinase-2 expression and bladder carcinoma metastasis by halofuginone	ECMs,MMP2,TGF-β1，	
Guan YF et al.	1999	10935488	1	Expression of peroxisome proliferator-activated receptor gamma (PPARgamma) in human transitional bladder cancer and its role in inducing cell death	PPARγ,p21,a-FABP,RXRα,cyclin D1,p16,15-PGDH,keratin13,EGF	
Haley JL et al.	1999	10233685	1	Enhancing the immunotherapeutic potential of mycobacteria by transfection with tumour necrosis factor-alpha	TNF-α,IL-1,IL-6,IL-8,IL-2,IL-5,IL-10,IL-12,IFN-γ,HLA-DR,MHC,ICAM-1,CR3/43,GM-CSF,RANTES,TGF-β 1,CD3,CD4,CD8,CD25	
Hudson MA et a	1999	10599442	1	Urokinase (u-PA) and the u-PA receptor. Modulation of in vitro invasiveness of human bladder cancer cell lines	u-PA,u-PAR,	
Hurst RE et al.	1999	10599447	1	Complexity, retinoid-responsive gene networks, and bladder carcinogenesis	hRARα,hRARβ,hRXRα,hRXRβ,hRXR γ,CRBP1,CRABP 1,CRABP 2,EGFR,ICAM1,aTRA,4-HPR,RARE,4-HPR,p53,bcl-2,AP-1,RARE,hAR,hER,β-Actin,MK,TG,EGFR,ICAM-1	
Imao T et al.	1999	9915485	1	Dominant role of E-cadherin in the progression of bladder cancer	E-cadherin,uPA,TIMP2,MMP1,MMP2,MMP9,c-ets-1,uPAR,PAI-1,PAI-2,MT1-MMP, Integrin-α2,Integrin-α 3,Integrin-α5,TFR	

Table S1. The list of bladder cancer related articles either used or not used RT4.

Author	Year	PMID	using RT4 or not(0 represents No;1 represents Yes)	Title	Gene	Pathway
Kawamata H et	1999	10493946	1	Balance between activated-STAT and MAP kinase regulates the growth of human bladder cell lines after treatment with epidermal growth factor	EGF,STAT1,STAT3,p21,EGFR,CDK, ERK1,ERK2,Ha-ras,	MAPK
Lawrence DS et	1999	10585203	0	Structure-activity studies of cerulenin analogues as protein palmitoylation inhibitors	p21,H-ras,N-ras,K-ras,	
Li SM et al.	1999	10458411	1	Detection of circulating uroplakin-positive cells in patients with transitional cell carcinoma of the bladder	UP1a,UP1b,UP2,UP3,	
Mack PC et al.	1999	10499638	0	RB status as a determinant of response to UCN-01 in non-small cell lung carcinoma	RB,p53,p16,PKC,	
Mizutani Y et al	1999	10425290	0	Chemoimmunosensitization of the T24 human bladder cancer line to Fas-mediated cytotoxicity and apoptosis by cisplatin and 5-fluorouracil	Fas	
Mizutani Y et al	1999	11810505	0	Effect of anticancer agents on Fas-mediated cytotoxicity against bladder cancer cells	Fas,TNF-α,TNF-β, CD40, CD27, CD30	
Mizutani Y et al	1999	10499639	0	Synergistic cytotoxicity and apoptosis by Apo-2 ligand and adriamycin against bladder cancer cells	Apo-2L,Fas,TNF-α,	
Ricol D et al.	1999	10602477	1	Tumour suppressive properties of fibroblast growth factor receptor 2-IIIb in human bladder cancer	FGFR1,FGFR2,FGFR3,FGFR4,FGF7,α PTyr,	
Takano H et al.	1999	10405635	0	Structural and functional analysis of the control region of the human DNA topoisomerase II alpha gene in drug-resistant cells	topo 2α	
Yamashita A et	1999	10490811	0	Suppression of anchorage-independent growth of human cancer cell lines by the drs gene	drs,EF-1,c-jun,c-myc,cyclinA,cyclinD,CyclinE,Cdk4,Cdk2,p21,p27,Rb,Tcf-4,h-drs,	APC-β-catenin,Ras-MAKP
Yeh CC et al.	1999	10547619	0	Effects of aspirin on arylamine N -acetyltransferase activity and DNA adducts in human bladder tumour cells	NAT,NAT2	
Zhang Y et al.	1999	10022737	1	Effects of bacillus Calmette-Guerin and interferon alpha-2B on cytokine production in human bladder cancer cell lines	IL-1β,IL-6,IL-8,GM-CSF,TGF-α,TGF-β2,IL-2,IFN-γ,TNF-α,IL-10,IL-20,INF-α,INF-γ	
Schul, W., et al.	1998	9457051	0	A subset of poly(A) polymerase is concentrated at sites of RNA synthesis and is associated with domains enriched in splicing factors and poly(A) RNA	CstF，CPSF PABII	
Retz, M., et al.	1998	9865718	1	Differential mucin MUC7 gene expression in invasive bladder carcinoma in contrast to uniform MUC1 and MUC2 gene expression in both normal urothelium and bladder carcinoma.	MUC1 MUC2 MUC7	
Nutt, J. E., et al	1998	9683296	1	Matrix metalloproteinase-1 is induced by epidermal growth factor in human bladder tumour cell lines and is detectable in urine of patients with bladder tumours	EGF MMP1 MMP2	
Mizutani, Y., et	1998	9679929	0	Sensitization of human bladder cancer cells to Fas-mediated cytotoxicity by cis-diamminedichloroplatinum (II)	Fas FasR	
Hamasaki, T., et	1998	9879815	0	Tumor progression and expression of matrix metalloproteinase-2 (MMP-2) mRNA by human urinary bladder cancer cells.	mmp2	

Table S1. The list of bladder cancer related articles either used or not used RT4.

Author	Year	PMID	using RT4 or not(0 represents No;1 represents Yes)	Title	Gene	Pathway
Furukawa, M., (	1998	9553115	0	The role of an inverted CCAAT element in transcriptional activation of the human DNA topoisomerase IIalpha gene by heat shock	topoIIalpha	
Bender, C. M., (	1998	9426064	0	Inhibition of DNA methylation by 5-aza-2'-deoxycytidine suppresses the growth of human tumor cell lines.	p16（CDKN2 / MTS1）	
Amann, B., et a	1998	9698673	1	Urinary levels of monocyte chemo-attractant protein-1 correlate with tumour stage and grade in patients with bladder cancer	MCP-1	
Bilim, V. N.	1997	14646556	1	Adriamycin (ADM) induced apoptosis in transitional cell cancer (TCC) cell lines accompanied by p21 WAF1/CIP1 induction	p53,p21	p53,p21, Bcl2,Bax
Bindels, E. M	1997	9299164	1	In vitro modulation of implantation and intraepithelial expansion of bladder tumor cells by epidermal growth factor	EGF	EGF
Bue, P	1997	9406687	1	The potential of radiolabeled EGF-dextran conjugates in the treatment of urinary bladder carcinoma	EGFr	
Dangles, V.	1997	9269996	0	Two- and three-dimensional cell structures govern epidermal growth factor survival function in human bladder carcinoma cell lines	EGF，P21	
Hudson, M. A	1997	9168186	1	Urokinase and the urokinase receptor: association with in vitro invasiveness of human bladder cancer cell lines	urokinase receptor	urokinase
Maack, S.	1997	21528226	1	Detection of differentially expressed genes in human bladder cancer cells using arbitrarily primed PCR of RNA	EGF	EGF
Okamoto, M	1997	9212236	1	Interleukin-6 functions as an autocrine growth factor in human bladder carcinoma cell lines in vitro	IL-6	IL-6
Tanabe, K	1997	9443649	1	Retroviral transduction of intercellular adhesion molecule-1 enhances endothelial attachment of bladder cancer	ICAM-1	ICAM-1
Tetzke, T. A.	1997	9344046	1	Effect of fibroblast growth factor saporin mitotoxins on human bladder cell lines	FGF2-SAP	FGF2-SAP
Zhang, Y.	1997	9180156	1	Effects of bacillus Calmette-Guerin and interferon-alpha-2B on human bladder cancer in vitro	IFN-α-2b	IFN-α-2b
Battelli MG1, P(	1996	8621232		Toxicity of ribosome-inactivating proteins-containing immunotoxins to a human bladder carcinoma cell line.	RIP，	
Brockhoff G1, I	1996	8844105	1	Options of flow cytometric three-colour DNA measurements to quantitate EGFR in subpopulations of human bladder cancer	EGFR	
Chresta CM Ma:	1996	8620501	1	Hypersensitivity of human testicular tumors to etoposide-induced apoptosis is associated with functional p53 and a high Bax:Bcl-2 ratio	p53,Bcl-2,Bax	
Cooper MJ1, Fi:	1996	8618347	0	Developmentally imprinted genes as markers for bladder tumor progression	H19, H19, IGF-II, IGF-I	
Danesi R 1，Na	1996	8649357	0	Phenylacetate inhibits protein isoprenylation and growth of the androgen-independent LNCaP prostate cancer cells transfected with the T24 Ha-ras oncogene	ras,p21,MAPK/ERK2	

Table S1. The list of bladder cancer related articles either used or not used RT4.

Author	Year	PMID	using RT4 or not(0 represents No;1 represents Yes)	Title	Gene	Pathway
Eklov, S.	1996	8712706	1	Estramustine-binding protein (EMBP) content in four different cell lines and its correlation to estramustine induced metaphase arrest	MAP	
Getzenberg RH	1996	8603421	0	Bladder Cancer-associated Nuclear Matrix Proteins	NMP	
Jin, F.	1996	9388337	0	The experimental study of expression of IL-2 cDNA in human bladder tumor cell and its biological behaviors	IL-2	
Kawasaki T1, T	1996	8945622	1	Abrogation of apoptosis induced by DNA-damaging agents in human bladder-cancer cell lines with p21/WAF1/CIP1 and/or p53 gene alterations	p21/WAF1/CIP1/p53	
Knuechel R1, S	1996	8863679	1	Connexin expression and intercellular communication in two- and three-dimensional in vitro cultures of human bladder carcinoma.	connexins Cx26, Cx32, and Cx43	
Konur, A.	1996		1	Human monocytes induce a carcinoma cell line to secrete high amounts of nitric oxide	NOS/ TNF-alpha/IL-1β	
Konur, A.	1996	8647627	0	Three-dimensional co-culture of human monocytes and macrophages with tumor cells: analysis of macrophage differentiation and activation	IL-1β/IL-6/MAX.3/CPM/TNF-alpha/ CD105	
Koss, A.	1996	8724544	0	Granulocyte-colony stimulating factor, granulocyte-macrophage colony stimulating factor and interleukin 4 induce differentiation in the U-937 human monocytic leukemia cell line	CD14 /CD11c/IL- 4	
Li, M.	1996	9275677	0	Inhibitory effects of malignant phenotype of human bladder cancer cell line by c-Ha-ras, c-myc antisense oligodeoxynucleotide	c-myc/ASO-R，ASO-M/rasp21，mycp62	
Margolis, E. J.	1996	8620437	0	Specific sequences of fibronectin activate the protein kinase C signal transduction pathway in invasive bladder cancer	PKC	
McConkey, D. J	1996	8895515	0	The human retinoblastoma gene product suppresses ceramide-induced apoptosis in human bladder tumor cells	RB	
Pu, Y. S.	1996	21541624	0	Expression of MDR-1 gene in transitional cell carcinoma and its correlation with chemotherapy response	MDR-1	
Saito, T.	1996	8562338	1	Correlation between integrin alpha 5 expression and the malignant phenotype of transitional cell carcinoma	integrin alpha 5 /integrin alpha 1/integrin alpha 2/integrin alpha3/integrin alpha 4/integrin alpha 1beta	
Schnier, J. B.	1996	8650198	0	G1 arrest and down-regulation of cyclin E/cyclin-dependent kinase 2 by the protein kinase inhibitor staurosporine are dependent on the retinoblastoma protein in the bladder carcinoma cell line 5637	p21，p27/cyclin E/cyclin-dependent kinase 2/CDK4/p21（Waf1 / Cip1）和 p27（Kip1）/cyclin D1	
Wakatsuki, S.	1996	8616803	1	Loss of human E-cadherin (ECD) correlated with invasiveness of transitional cell cancer in the renal pelvis, ureter and urinary bladder	ECD/α-catenin /HECD-1	
Wu Q1, Possati	1996	8631601	1	Growth arrest and suppression of tumorigenicity of bladder-carcinoma cell lines induced by the P16/CDKN2 (p16INK4A, MTS1) gene and other loci on human chromosome 9	P16/CDKN2 (p16INK4A, MTS1)	

Table S1. The list of bladder cancer related articles either used or not used RT4.

Author	Year	PMID	using RT4 or not(0 represents No;1 represents Yes)	Title	Gene	Pathway
Sion-Vardy, N.	1995	8538164	1	Antiproliferative effects of tyrosine kinase inhibitors (tyrphostins) on human bladder and renal carcinoma cells	EGF-r,c-erbB2,RG14620,AG555	
Yokomizo, A.	1995	7671238		Cellular levels of thioredoxin associated with drug sensitivity to cisplatin, mitomycin C, doxorubicin, and etoposide	TRX	
Fiorentini, P.	1995	7575504	0	Characterization of a distal 5'-flanking region (-2010/-630) of human GM-CSF	GM-CSF	
Alexandroff, A.	1995	8562166	1	Cytokine modulation of epidermal growth factor receptor expression on bladder cancer cells is not a major contributor to the antitumour activity of cytokines	IL-1\TNF\EGFR\	
Suzuki, S.	1995	7669578	0	ytotoxicity of anti-c-erbB-2 immunoliposomes containing doxorubicin on human cancer cells	c-erbB-2	
Kestler, D. P.	1995	8541546	0	Detection and analysis of an alternatively spliced isoform of interleukin-6 mRNA in peripheral blood mononuclear cells	lL-6	
Bailly, J. D.	1995	7564516	0	Effect of 5637-conditioned medium and recombinant cytokines on P-glycoprotein expression in a human GM-CSF-dependent leukemic myeloid cell line	GM-CSF, G-CSF, IL-1 beta, IL-6, stem cell factor, LIF, erythropoietin, and IL-3	
Nouri, A. M.	1995	7544146	0	Epidermal growth factor-induced protection of tumour cell susceptibility to cytolysis	EGF and EGFR	
Hasegawa, S.	1995	7734314	0	Expression of multidrug resistance-associated protein (MRP), MDR1 and DNA topoisomerase II in human multidrug-resistant bladder cancer cell lines	MRP and MDR1	
Pagliaro LC et a	1995	7669721	0	Recombinant human retinoblastoma protein inhibits cancer cell growth	RB1	
Cheng YT et al.	1995	7619220	0	Overexpression of MDM-2 mRNA and mutation of the p53 tumor suppressor gene in bladder carcinoma cell lines	MDM-2	
Penco S et al.	1995	7744878	0	Lactoferrin down-modulates the activity of the granulocyte macrophage colony-stimulating factor promoter in interleukin-1 beta-stimulated cells	GM-CSF、IL- 1、	
Kageyama Y et	1995	7474403	0	Infrequent mutations of the WT1 gene in primary cancers of the adult urinary tract	WT1	
Colquhoun A et	1995	8589632	0	Human and rat tumour cells possess mitochondrial carnitine palmitoyltransferase I and II: effects of insulin	CPT I、CPT II	
Zhou, Y.	1994	8183886	0	Further characterization of retinoblastoma gene-mediated cell growth and tumor suppression in human cancer cells	SV40、CMV、RB33、RB35	
Zarbo, R. J.	1994	7513944	0	Rapid (one-shot) staining method for two-color multiparametric DNA flow cytometric analysis of carcinomas using staining for cytokeratin and leukocyte common antigen	CD45	
Watanabe, N.	1994	7893968	0	Selective release of a processed form of interleukin 1 alpha	IL-1α	
Uchibayashi, T.	1994	7994794	0	Studies of effects of anticancer agents in combination with/without hyperthermia on metastasized human bladder cancer cells in chick embryos using the polymerase chain reaction technique	β-globin	

Table S1. The list of bladder cancer related articles either used or not used RT4.

Author	Year	PMID	using RT4 or not(0 represents No;1 represents Yes)	Title	Gene	Pathway
Mizutani, Y.	1994	7923012	0	Enhancement of sensitivity of urinary bladder tumor cells to cisplatin by c-myc antisense oligonucleotide	c-myc	
Lee, S. S.	1994	8012943	0	Intravesical gene therapy: in vivo gene transfer using recombinant vaccinia virus vectors	H1、NP	
Kotoh, S.	1994	8205547	0	Increased expression of DNA topoisomerase I gene and collateral sensitivity to camptothecin in human cisplatin-resistant bladder cancer cells	Topo I、Topo II、GST-π	
Kanbe, T	1994	8069262	0	Phenotypic reversion induced by anthracyclines in ras oncogene-expressed cells; structure-activity relationships	K-ras、H-ras	
Kamei, H.	1994	7954871	0	Relationship of nuclear invaginations to perinuclear rings composed of intermediate filaments in MIA PaCa-2 and some other cells	AC36、AC19、ZSV5、MAK-5、CKT1、CKT2、BSA	
Hayashi, O.	1994	8308999	0	Detection of interleukin-1 activity in human bladder cancer cell lines	IL-1	
Cooper, M. J.	1994	7787250	1	p53 mutations in bladder carcinoma cell lines	p53	
Campbell, S. C.	1994	7908992	0	Intercellular adhesion molecule-1 expression by bladder cancer cells: functional effects	ICAM-1	
Brockhoff, G.	1994	8001460	1	Flow cytometric detection and quantitation of the epidermal growth factor receptor in comparison to Scatchard analysis in human bladder carcinoma cell lines	EGFR1	
Ali AA, Harvey	1993	8343169	0	Retinoblastoma gene product-associated proteins in human colon cancer cell lines	RB1,RAP	
Allen LE, Mahe	1993	8387074	1	Expression of basic fibroblast growth factor and its receptor in an invasive bladder carcinoma cell line	b-FGF	
Gray GD, Herna	1993	8425190	0	Antisense DNA inhibition of tumor growth induced by c-Ha-ras oncogene in nude mice	c-Ha-ras,p21	
Kameyama S, K	1993	8353837	1	A new in vivo model for studying invasion and metastasis of rat and human bladder carcinomas.	hGH	
Kitamura M, Sh	1993	8434639	0	A retinoid responsive cytokine gene, MK, is preferentially expressed in the proximal tubules of the kidney and human tumor cell lines	MK	
Penning LC, Ke	1993	8503851	0	Ca(2+)-mediated prostaglandin E2 induction reduces haematoporphyrin-derivative-induced cytotoxicity of T24 human bladder transitional carcinoma cells in vitro	PGE2，TXB2	
Tomita Y, Wata	1993	8100398	0	Expression of intercellular adhesion molecule-1 on transitional cell cancer. Possible significance in immunity against tumor cells	ICAM-1	
Weismanová E,	1993	7903794	0	c-Ha-ras BamHI RFLP in human urothelial tumors and point mutations in hot codons	c-Ha-ras	

Table S1. The list of bladder cancer related articles either used or not used RT4.

Author	Year	PMID	using RT4 or not(0 represents No;1 represents Yes)	Title	Gene	Pathway
Tanaka, H. and	1992	1625212	0	Pharmacokinetic and pharmacodynamic comparisons between human granulocyte colony-stimulating factor purified from human bladder carcinoma cell line 5637 culture medium and recombinant human granulocyte colony-stimulating factor produced in Escherichia coli.	hG-CSF	
Mizutani, Y., et	1992	1433570	0	Effect of PSK and its subfractions on peripheral blood lymphocytes mediated cytotoxicity against urinary bladder tumor cells."	PSK	
Jackson, A. M.,	1992	1353063	1	Role of adhesion molecules in lymphokine-activated killer cell killing of bladder cancer cells: further evidence for a third ligand for leucocyte function-associated antigen-1	ICAM-1 ICAM-2	
Jackson, A. M.,	1992	1433572	1	Expression of adhesion molecules by bladder cancer cells: modulation by interferon-gamma and tumour necrosis factor-alpha.	interferon-gamma tumour necrosis factor-alpha. adhesion molecules1 adhesion molecules2	
Czerniak, B et a	1992	1427748	0	Concurrent mutations of coding and regulatory sequences of the Ha-ras gene in urinary bladder carcinoma	Ha-ras、ras p21	
de Harven, E. et	1992	1591726		Antibody drug carrier for immunotherapy of superficial bladder cancer: ultrastructural studies	48-127mAb	
Fu, X. et al.	1992	1639544	1	Human RT-4 bladder carcinoma is highly metastatic in nude mice and comparable to ras-H-transformed RT-4 when orthotopically onplanted as histologically intact tissue	Hras	
Lebeau J, Le Ch	1991	1861862	0	Constitutive overexpression of a 89 kDa heat shock protein gene in the HBL100 human mammary cell line converted to a tumorigenic phenotype by the EJ/T24 Harvey-ras oncogene	ras,hsp89	
Mizutani Y, Yo:	1991	2016797	0	Activation by the protein-bound polysaccharide PSK (krestin) of cytotoxic lymphocytes that act on fresh autologous tumor cells and T24 human urinary bladder transitional carcinoma cell line in patients with urinary bladder cancer	PSK,IFN-a,IFN-r,IL-2	
Otsuka T, Hum	1991	1717496	0	Continuous activation of primitive hematopoietic cells in long-term human marrow cultures containing irradiated tumor cells	IL-1,IL-6	
Saison-Behmoa	1991	1850694	0	Short modified antisense oligonucleotides directed against Ha-ras point mutation induce selective cleavage of the mRNA and inhibit T24 cells proliferation	ras	
Sorg R, Enczma	1991	1893964	0	Rapid and sensitive mRNA phenotyping for interleukins (IL-1 to IL-6) and colony-stimulating factors (G-CSF, M-CSF, and GM-CSF) by reverse transcription and subsequent polymerase chain reaction	IL-1,IL-2,IL-3,IL-4,IL-5,IL-6,G-CSF, M-CSF, GM-CSF	
Zucker et.al	1990	2202421	0	Purification and characterisation of soluble tumour haemolytic factor isolated from oncogene transformed fibroblasts	THF	
Weiss et.al	1990	2201799	1	Mechanisms of human bladder tumor invasion: role of protease cathepsin B	cathepsin B	

Table S1. The list of bladder cancer related articles either used or not used RT4.

Author	Year	PMID	using RT4 or not(0 represents No;1 represents Yes)	Title	Gene	Pathway
Theodorescu et.	1990	2247480	1	Overexpression of normal and mutated forms of HRAS induces orthotopic bladder invasion in a human transitional cell carcinoma	HRAS	
Stacey et.al	1990	1698381	0	Deregulation in trans or c-myc expression in immortalized human urothelial cells and in T24 bladder carcinoma cells	c-myc,	
Schwartz et.al	1990	2231770	1	Inhibition of invasion of invasive human bladder carcinoma cells by protein kinase C inhibitor staurosporine	PKC,	
Oez et.al	1990	2209765	0	A highly sensitive quantitative bioassay for human granulocyte-macrophage colony-stimulating factor	GM- csf	
Morioka et.al	1990	1699255	0	Purification of a granulocyte colony-stimulating factor from the conditioned medium of a subclone of human bladder carcinoma cell line 5637, HTB9	CSF	
Li et.al	1990	2196114	0	Thermal response of oncogene-transfected rat cells	c-myc , c-Ha-ras	
Kobayashi et.al	1990	2115174	0	Identification of calcium-activated neutral protease as a processing enzyme of human interleukin 1 alpha	CANP,IL-1,	
Hermann et.al	1990	1707414	0	Reduced LAK cytotoxicity of peripheral blood mononuclear cells in patients with bladder cancer: decreased LAK cytotoxicity caused by a low incidence of CD56+ and CD57+ mononuclear blood cells	IL-2,CD56 ,CD57	
Harris et.al	1990	2408158	0	Some ras-transformed cells have increased radiosensitivity and decreased repair of sublethal radiation damage	H-ras,K-ras,	
Grups et.al	1990	2140214	1	Interferon receptors on the surface of interferon-sensitive and interferon-resistant urothelial carcinomas	IFN,	
Gauthier et.al	1990	2403838	0	Growth-regulated surface glycoproteins of human bladder cancer	p21,	
Mizutani, Y.	1989	2509333	0	Enhancement by X-ray irradiation of target cell susceptibility to natural killer cells	IFN	
McNiece, I. K.	1989	2645951	0	Studies on the myeloid synergistic factor from 5637: comparison with interleukin-1 alpha	IL-1	
Longin, A.	1989	2645951	0	A monoclonal antibody (BL2-10D1) reacting with a bladder-cancer-associated antigen	IL-1	
Hurlin, P. J.	1989	2643097	0	Malignant transformation of human fibroblasts caused by expression of a transfected T24 HRAS oncogene	T24-HRAS、HRAS	
Hsiao, W. L.	1989	2474757	0	Cells that overproduce protein kinase C are more susceptible to transformation by an activated H-ras oncogene	PKC、**cH-ras**	
Donato, N. J.	1989	2555361	0	Tumor necrosis factor modulates epidermal growth factor receptor phosphorylation and kinase activity in human tumor cells. Correlation with cytotoxicity	EGF	
Soma, G.	1988	2851034	0	Biological activities of novel recombinant tumor necrosis factor having N-terminal amino acid sequences derived from cytotoxic factors produced by THP-1 cells	cH-ras	
Senger, D. R.	1988	3293049	0	T24 human bladder carcinoma cells with activated Ha-ras protooncogene: nontumorigenic cells susceptible to malignant transformation with carcinogen	cH-ras	

Table S1. The list of bladder cancer related articles either used or not used RT4.

Author	Year	PMID	using RT4 or not(0 represents No;1 represents Yes)	Title	Gene	Pathway
Reznikoff, C. A	1988	2841047	0	Neoplastic transformation of SV40-immortalized human urinary tract epithelial cells by in vitro exposure to 3-methylcholanthrene	ras、p21	
Moll, R.	1988	2456018	1	Cytokeratins in normal and malignant transitional epithelium. Maintenance of expression of urothelial differentiation features in transitional cell carcinomas and bladder carcinoma cell culture lines	simple-epithelium-type cytokeratins	
Hsieh, J. T.	1988	3355171	0	Involvement of protein kinase C in the transcriptional regulation of ornithine decarboxylase gene expression by 12-O-tetradecanoylphorbol-13-acetate in T24 human bladder carcinoma cells	ODC	
Hill, S. A.	1988	3285012	0	Clonal heterogeneity, experimental metastatic ability, and p21 expression in H-ras-transformed NIH 3T3 cells	H-ras、p21	
Fraser, J. K.	1988	2825843	0	Expression and modulation of specific, high affinity binding sites for erythropoietin on the human erythroleukemic cell line K562	Epo、IL-3、EPA	
Craig, A. M.	1988	3058125	0	Identification of the major phosphoprotein secreted by many rodent cell lines as 2ar/osteopontin: enhanced expression in H-ras-transformed 3T3 cells	H-ras	
Berzins, T.	1988	2975040	0	Characterization of human CD4+ T-cell clones that secrete helper factor(s) for B-cell proliferation and maturation	CD45R、CDw29	
Horan Hand, P.	1987	3814600	0	Absolute values of ras p21 defined by direct binding liquid competition radioimmunoassays	ras	
Hader, M.	1987	3316242	0	Epidermal growth factor receptor expression, proliferation, and colony stimulating activity production in the urinary bladder carcinoma cell line 5637	EGF	
Flatow U et al.	1987	3301693	0	Tumorigenicity of T24 urinary bladder carcinoma cell sublines	HRAS、P21	
Bondy, G. P.	1985	4063960	0	Experimental metastatic ability of H-ras-transformed NIH3T3 cells	H-ras	
Chang, E. H.	1985	3842394	0	Pathogenicity of retroviruses containing either the normal human c-Ha-ras1 gene or its mutated form derived from the bladder carcinoma EJ/T24 cell line	c-Ha-ras1	
Greig, R. G.	1985	3858844	0	Tumorigenic and metastatic properties of "normal" and ras-transfected NIH/3T3 cells	c-Ha-ras1	
Gross, M.	1985	3923330	0	Purification and characterization of human H-ras proteins expressed in Escherichia coli	H-ras	
Hubbell, H. R.	1985	3986788	1	Antiproliferative and immunomodulatory actions of beta-interferon and double-stranded RNA, individually and in combination, on human bladder tumor xenografts in nude mice	IFN-β	
Samid, D	1985	3970704	0	Development of transformed phenotype induced by a human ras oncogene is inhibited by interferon	ras	
Samid, D	1985	2417251	0	Interferon-induced modulation of human ras oncogene expression	ha-ras	
Tabin, C. J	1985	2981345	0	Analysis of viral and somatic activations of the cHa-ras gene	cHa-ras	

Table S1. The list of bladder cancer related articles either used or not used RT4.

Author	Year	PMID	using RT4 or not(0 represents No;1 represents Yes)	Title	Gene	Pathway
Borden, E. C	1984	6206246	1	Antiproliferative activities of interferons against human bladder carcinoma cell lines in vitro	Interferon alpha 54, 76, 61, 6L	Interferon
Czerniak, B.	1984	6432310	0	Expression of Ca antigen in relation to cell cycle in cultured human tumor cells	Ca antigen	Ca antigen
Groveman, D. S	1984	6498814	1	Augmented antiproliferative effects of interferons at elevated temperatures against human bladder carcinoma cell lines	IFN-alpha and IFN-beta	IFN-alpha and IFN-beta
Ramaekers, F. C	1984	6203768	0	Flow-cytometric analysis of mixed cell populations using intermediate filament antibodies	cytokeratin	cytokeratin
Rousset, M.	1984	6418376	1	Growth-related enzymatic control of glycogen metabolism in cultured human tumor cells	glycogen synthase and phosphorylase	glycogen accumulation
Soslau, G.	1984	6424675	1	Phosphoproteins altered by antiproliferative doses of human interferon- beta in a human bladder carcinoma cell line	IFN-beta	
Trejdosiewicz, I	1984	6198551	1	Species cross-reactive membrane-associated urothelial differentiation antigen	urothelium membrane antigen	
Bevan HJ et al.	1983	6351893	0	Detection of anti-intercellular cement substance and anti-basement membrane zone antibodies by radioimmunoassay using SCaBER tumour cell line as substrate	ICS,BMZ	
et al.	1983	6298635	0	Complete nucleotide sequences of the T24 human bladder carcinoma oncogene and its normal homologue	c-Ha-ras-1,c-Ha-ras-2,v-Ha-ras,v-Ki-ras,p21,c-Ha-ras,Ha-ras,	
Fasano O et al.	1983	6308118	0	Sequence and structure of the coding region of the human H-ras-1 gene from T24 bladder carcinoma cells	H-ras-1,ras	
Grossman HB et	1983	6887389	1	Hybridoma antibodies reactive with human bladder carcinoma cell surface antigens	NS-1,	
O'Toole CM et	1983	6823318	1	Identity of some human bladder cancer cell lines	HLA,HLA-A1,HLA-A2,HLA-A25,HLA-B7,HLA-B18,HLA-Aw32,HLA-Bw51,HLA-Bw44,HLA-Cw5,HLA-A3,HLA-B18,HLA-Aw32,HLA-B18,HLA-B5,PGM1,PGM3,GOT2,ESD,AK1,ADA,ACP1,PGD,G6PD,PEPA,PEPC,GLO,FUC,PGP,GDH	
Pincus MR et a	1983	6577419	0	Prediction of the three-dimensional structure of the transforming region of the EJ/T24 human bladder oncogene product and its normal cellular homologue	p21,c-Ha ras,c-onc	
Reddy EP et al.	1983	6844927	0	Nucleotide sequence analysis of the T24 human bladder carcinoma oncogene	v-has,v-bas	
Santos E et al.	1983	6308640	0	Spontaneous activation of a human proto-oncogene	p21,c-has,c-bas	
Steele JG et al.	1983	6871191	1	Identification of exposed surface glycoproteins of four human bladder carcinoma cell lines	CP-175,GP-155,GP-145,GP-130,GP-110,GP-200,GP-175,NaB4,NaIO4	
Wierenga RK e	1983	6843652	0	Predicted nucleotide-binding properties of p21 protein and its cancer-associated variant	p21,	

Table S1. The list of bladder cancer related articles either used or not used RT4.

Author	Year	PMID	using RT4 or not(0 represents No;1 represents Yes)	Title	Gene	Pathway
Yuasa Y et al.	1983	6866079	0	Acquisition of transforming properties by alternative point mutations within c-bas/has human proto-oncogene	c-bas,c-has	
Lajzerowicz M	1982	6960193	0	A study of the immune response to the organ-specific neoantigen of human bladder cancer	PGE2	
McBride OW et	1982	7177197	0	Localization of the normal allele of T24 human bladder carcinoma oncogene to chromosome 11	LDH-A, EsA4,mos,fes,myb,abl	
Reddy EP et al.	1982	7133135	0	A point mutation is responsible for the acquisition of transforming properties by the T24 human bladder carcinoma oncogene	v-has,v-bas,c-has,c-bas-1	
Santos E et al.	1982	6283384	0	T24 human bladder carcinoma oncogene is an activated form of the normal human homologue of BALB- and Harvey-MSV transforming genes	v-bas,v-myc,c-myc,v-myb,v-src	
Taparowsky E e	1982	7177195	0	Activation of the T24 bladder carcinoma transforming gene is linked to a single amino acid change	H-ras-1,v-H-ras,p21,ras	
Zhao, X et al.	2018	30410328	0	Photothermal exposure of polydopamine-coated branched Au-Ag nanoparticles induces cell cycle arrest, apoptosis, and autophagy in human bladder cancer cells		AKT/ERK
Zeng LP1, Hu Z	2016	26800397	0	miR-222 attenuates cisplatin-induced cell death by targeting the PPP2R2A/Akt/mTOR Axis in bladder cancer cells		PPP2R2A/Akt/mTOR axis
Wu TF1, Hsu L'	2016	26955879	0	Clarification of the molecular pathway of Taiwan local pomegranate fruit juice underlying the inhibition of urinary bladder urothelial carcinoma cell by proteomics strategy		AKT/mTOR signaling pathway
Wang YY1, Wu	2016	27631965	0	LINC00312 inhibits the migration and invasion of bladder cancer cells by targeting miR-197-3p		LINC00312/miR-197-3p
Tao L#1,2, Qiu	2016	27196763	0	Infiltrating T Cells Promote Bladder Cancer Progression via Increasing IL1-->Androgen Receptor-->HIF1alpha-->VEGFa Signals		IL1→AR→HIF1α→VEGFa signaling
Kawahara T1, I	2016	27330033	0	Enzalutamide inhibits androgen receptor-positive bladder cancer cell growth		AR pathway
Shin, D. Y.	2014	24309133	0	Diallyl trisulfide-induced apoptosis of bladder cancer cells is caspase-dependent and regulated by PI3K/Akt and JNK pathways		PI3K/Akt and JNK/p38 MAPK/Bcl-2 and Bcl-Xl/
Xiong, D.	2014	24768914	0	Down-regulating ribonuclease inhibitor enhances metastasis of bladder cancer cells through regulating epithelial-mesenchymal transition and ILK signaling pathway		ILK
Pinto-Leite R et	2014	24035472	0	Temsirolimus improves cytotoxic efficacy of cisplatin and gemcitabine against urinary bladder cancer cell lines		AKT-mTOR
Zhang S et al.	2014	24687305	0	Maslinic acid induced apoptosis in bladder cancer cells through activating p38 MAPK signaling pathway		p38 MAPK
Du, H.et al.	2013	23368424	0	Cytotoxicity and oxidative damage induced by halobenzoquinones to T24 bladder cancer cells		ROS pathway
Azuma, H.	2003	12771800	0	Induction of apoptosis in human bladder cancer cells in vitro and in vivo caused by FTY720 treatment		p42 / p44 pathway

Table S1. The list of bladder cancer related articles either used or not used RT4.

Author	Year	PMID	using RT4 or not(0 represents No;1 represents Yes)	Title	Gene	Pathway
van Bokhoven,	2001	11522622	0	TSU-Pr1 and JCA-1 cells are derivatives of T24 bladder carcinoma cells and are not of prostatic origin		p53 and Ha- ras

Table S2. The list of genes in FGFR3 pathway and P53

genes in FGFR3 pathway	genes in P53 pathway
FGFR3	PTEN
FGF	APAF1
EGF	CDK1
GF	CDK4
FGF2	CDK6
miR-10	BAX
SHC	BCL2
Crk	CASP3
Cb1	CDK2
Grb2	CHEK1
Ras	SERPINE1
miR-23	TNFRSF6
SHP2	CASP8
C3G	CASP9
Epsin	GADD45
SOS	TP53
miR-103	CCND1
PLCγ	SIAH1
CIN85	BCL2L1
miR101	ADGRB1
ERK	BID
RafB	ATM
EPS15	IGF1
Raf	CCNB1
Erk1	KAI1
Erk2	CDKN2A
MEK	CDKN1A
c-Myc	CCNE
endophilin	ATR
PI3K	CHEK2
Akt	MDM2
Akt/P	SFN
	TSC2
	CYC
	MDM4
	RPRM
	GTSE1
	LRDD
	PMAIP1
	BBC3
	TP53I3
	EI24
	SCOTIN
	PERP
	ZMAT3
	IGFBP3
	SERPINB5
	DDB2
	SESN1_3
	STEAP3

Table S2. The list of genes in FGFR3 pathway and P53

genes in FGFR3 pathway	genes in P53 pathway
	RFWD2
	RCHY1
	CCNG1
	CCNG2
	PPM1D
	TP73
	CCND2
	CCND3
	RRM2
	TP53AIP1
	THBS1
	GORAB
	SESN2
	CCNB2
	SIVA1
	AIFM2

Table S3. The numbers of using cell line RT4 for research on that particular target gene.

gene	all	RT4
FGFR3	81	6
FGF	39	3
EGF	72	21
FGF2	32	2
miR-10	0	0
SHC	2	0
Crk	6	0
Cb1	3	1
Grb2	4	0
Ras	276	11
miR-23	0	0
SHP2	3	0
C3G	0	0
Epsin	0	0
SOS	1	1
miR-103	0	0
PLCγ	8	1
CIN85	0	0
miR101	3	0
ERK	122	9
RafB	0	0
EPS15	0	0
Raf	27	3
Erk1	62	7
Erk2	4	1
MEK	24	4
c-Myc	104	7
endophilin	1	1
PI3K	128	10
Akt	305	35
PTEN	63	4
APAF1	3	1
CDK1	13	3
CDK4	42	1
CDK6	24	1
BAX	130	13
BCL2	38	4
CASP3	259	28
CDK2	29	4
CHEK1	7	0
SERPINE1	3	0
TNFRSF6	1	0
CASP8	15	4
CASP9	13	1
GADD45	1	0
TP53	62	17
CCND1	39	0
SIAH1	1	0
BCL2L1	34	0
ADGRB1	0	0
BID	17	3
ATM	19	3
IGF1	3	0
CCNB1	40	3

Table S3. The numbers of using cell line RT4 for research on that particular target gene.

gene	all	RT4
KAI1	10	0
CDKN2A	54	2
CDKN1A	45	9
CCNE	40	1
ATR	9	0
CHEK2	9	1
MDM2	24	0
SFN	6	0
TSC2	3	0
CYC	0	0
MDM4	2	0
RPRM	1	0
GTSE1	1	0
LRDD	0	0
PMAIP1	0	0
BBC3	1	1
TP53I3	0	0
EI24	0	0
SCOTIN	0	0
PERP	1	0
ZMAT3	0	0
IGFBP3	6	0
SERPINB5	1	0
DDB2	0	0
SESN1_3	0	0
STEAP3	1	0
RFWD2	0	0
RCHY1	0	0
CCNG1	2	0
CCNG2	1	1
PPM1D	2	1
TP73	3	0
CCND2	2	0
CCND3	0	0
RRM2	6	0
TP53AIP1	0	0
THBS1	5	3
GORAB	0	0
SESN2	1	0
CCNB2	2	0
SIVA1	0	0
AIFM2	1	0

Table S4. Clinical characteristics of the patients included in the study.

ID	Age (yr)	Gender	Symptom	Tumor
1214785	55	Male	Gross hematuria	NMIBC
1260287	57	Male	Gross hematuria	MIBC
